# Isolation, total synthesis, and biological evaluation of dearomatized isoprenylated acylphloroglucinols from *Hypericum przewalskii*

**DOI:** 10.1007/s13659-026-00626-y

**Published:** 2026-04-01

**Authors:** Yong Li, Fei-Fei Xiong, Xiao-Yang Sun, Xing-Ren Li, Dao-Feng Chen, Gang Xu, Yin Nian, Li-Dong Shao

**Affiliations:** 1 Yunnan Key Laboratory of Southern Medicinal Utilization, School of Chinese Materia Medica, Yunnan University of Chinese Medicine, Kunming, 650500 China; 2 Key Laboratory of Phytochemistry and Natural Medicines, Kunming Institute of Botany, Chinese Academy of Sciences, Kunming, 650201 China; 3 University of Chinese Academy of Sciences, Beijing, 100049 China; 4 School of Pharmacy, Institutes of Integrative Medicine, Fudan University, Shanghai, 201203 China

**Keywords:** *Hypericum przewalskii*, Dearomatized isoprenylated acylphloroglucinols (DIAPs), Total synthesis, T-type calcium channels Ca_v_3.2

## Abstract

New dearomatized isoprenylated acylphloroglucinols hyperprzewones A (**1**) and B (**2**) were isolated and characterized from the dried aerial parts of *Hypericum przewalskii*. The structures of these compounds were confirmed by extensive spectroscopic experiments. A facile synthetic route to **1** and **2** was developed via Friedel-Crafts acylation, alkylation, dearomatization, and oxidative [4 + 2] cyclization, giving a 17% overall yield. Moreover, the synthetic derivative **8** exhibited moderate inhibition on T-type calcium channels Ca_v_3.2.

**Graphical Abstract:**

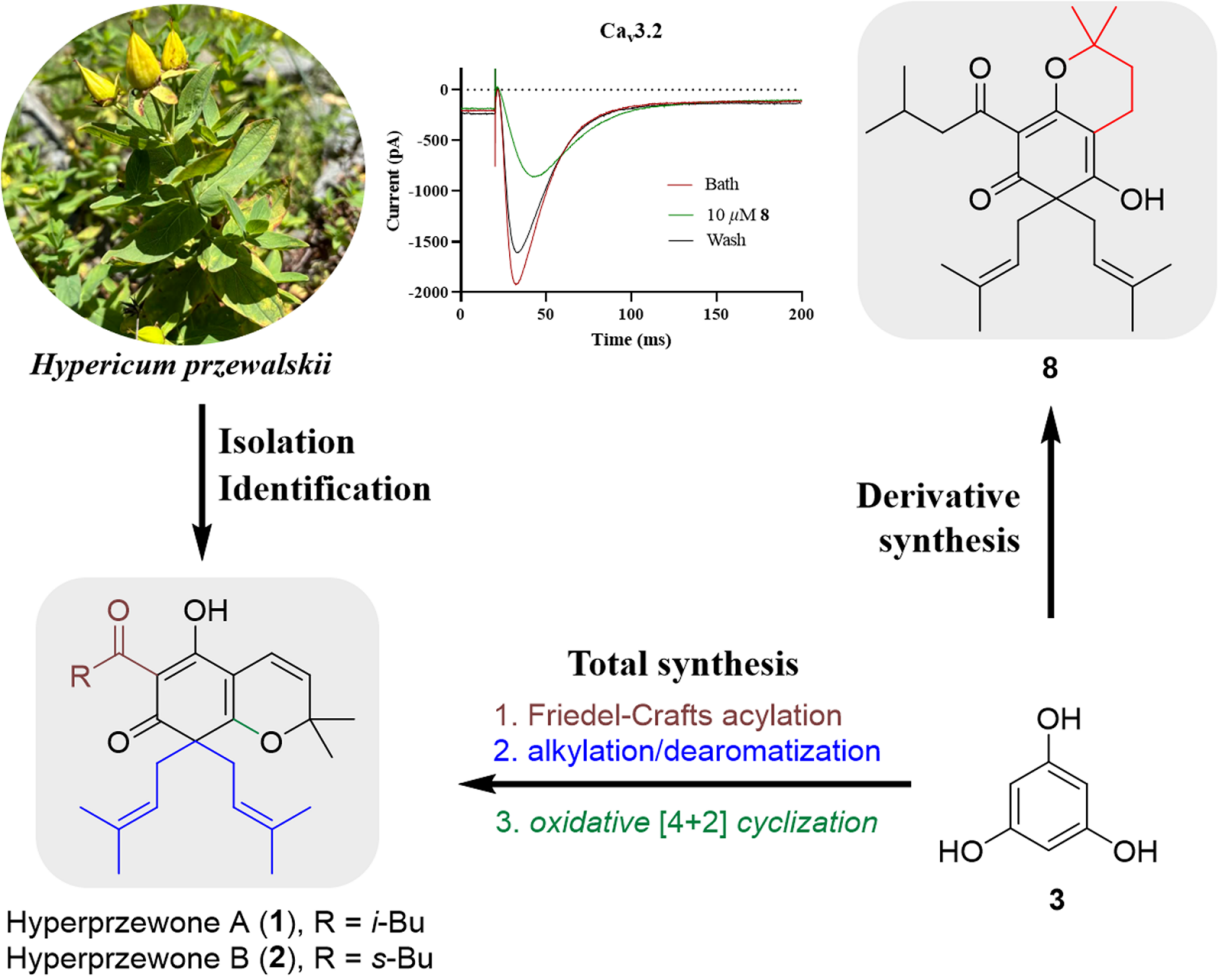

**Supplementary Information:**

The online version contains supplementary material available at 10.1007/s13659-026-00626-y.

## Introduction

Natural phloroglucinols are widely distributed in plants of families such as Myrtaceae, Euphorbiaceae, Clusiaceae, Dryopteridaceae, Asteraceae, Fabaceae, and Rutaceae, as well as in marine and microbial sources [1]. Among them, prenylated phloroglucinols are a special type of hybrid natural products derived from polyketide combined with isoprenylation biosynthetic pathways, and mainly reported from plants of the genera *Hypericum* and *Garcinia* in the family of Guttiferae [2–4]. We have isolated and identified two new dearomatized isoprenylated acylphloroglucinols hyperprzewones A and B (**1** and** 2**) from the *Hypericum przewalskii*. However, there are relatively few reports on the synthesis of such natural products. Guan [5]first achieved the asymmetric total synthesis of hyperbeanol A through a bioinspired alkylation dearomatization reaction. We adopted a concise and effective synthetic route to synthesize two new dearomatized isoprenylated natural acylphloroglucinols, and synthesize a series of derivatives based on **1** and** 2**. They provide a reference for subsequent synthesis research and development of DIAPs-type natural products [6, 7].

**Fig. 1:**
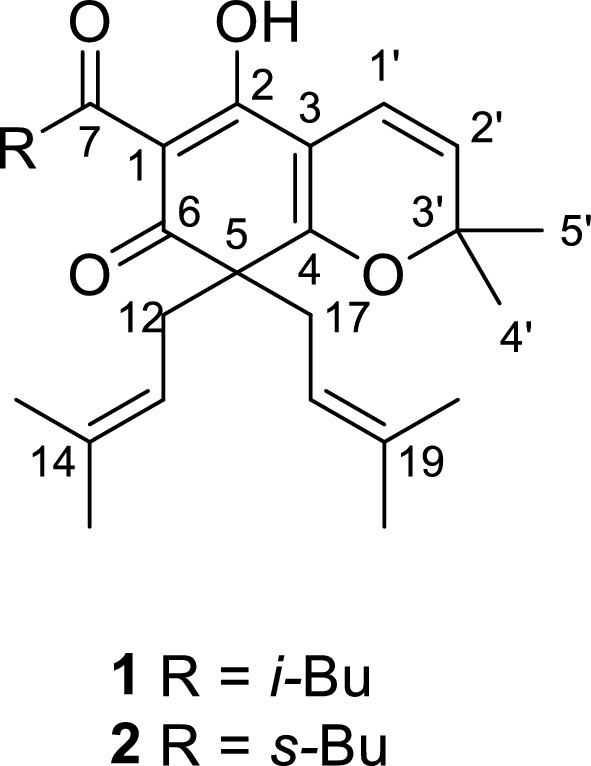
Structures of hyperprzewones A (**1**) and B (**2**)

T-type calcium channels (TTCCs), as a key subtype of voltage-gated calcium channels (VGCCs), are renowned for their unique electrophysiological characteristics, including low activation threshold, brief opening, and rapid inactivation [8, 9]. Its functional abnormalities are closely related to various diseases, including neuropathic pain, epilepsy, hypertension, and cardiovascular diseases, making it an important direction for the development of new targeted drugs [10, 11]. In this study, we targeted Ca_v_3.2 channel heterologously expressed in HEK-293 T cells, and screened two natural products **1** and** 2**, as well as a series of their synthetic derivatives, using the whole-cell patch-clamp recording method [12, 13]. The results showed that derivatives **8**, **10**, and **14** exhibit inhibitory effects on T-type calcium channels Ca_v_3.2. Fig. 1 structures of hyperprzewones A (**1**) and B (**2**).

## Results and discussion

**Fig. 2:**
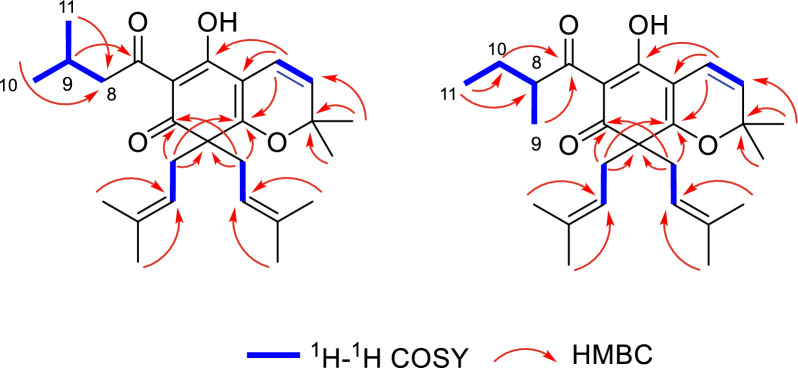
Key HMBC and ^1^H–^1^H COSY correlations of **1** and **2**

Compound **1** was acquired as a brown oil. Combining the negative-ion HRESIMS peak at *m*/*z* 411.2549 [M–H]^−^ and ^13^C NMR revealed a molecular formula of C_26_H_36_O_4_. The IR spectrum implied absorption bands for hydroxyl (3433 cm^−1^) and carbonyl (1634 cm^−1^) functionalities. The ^1^H NMR data (Table 1) illustrated four olefinic protons (*δ*_H_ 4.8, 4.8, 5.5, 6.4) and an isobutyl group (*δ*_H_ 0.95, d, *J* = 6.8 Hz; 2.07, dq, *J* = 13.4, 6.4 Hz; 2.91, d, *J* = 7.0 Hz). The ^13^C NMR (Table 1) and DEPT spectra showed 26 carbons including eight methyls, five methylenes, three methines, and 10 quaternary carbons. particularly, obvious signals for a typical dearomatized phloroglucinol core (*δ*_C_ 107.6, C–1; 187.5, C–2; 109.2, C–3; 173.4, C–4; 58.2, C–5; 197.3, C–6). Two isoprenyls and an isopropyl of DIAPs-type metabolites can be distinguished [14]. In the HMBC spectrum, the key correlations (Fig. 2) from H_2_–12/H_2_–17 to C–4, C–5 and C–6 confirmed that two isoprenyls were both attached to C–5, the HMBC correlation from H–1' to C–2, C–3 and C–4 indicates that C–1' is connected to the benzene ring's C–3 (Fig. 2). The ether linkage of C–4 and C–3' was evidenced by indices of hydrogen deficiency, the downfield chemical shift of C–3' (*δ*_C_ 82.8) (Fig. 2). Based on the key ^1^H–^1^H COSY correlations of H–1' and H–2', as well as the HMBC correlations between Me–5' and C–3', C–2', combined with the ^13^C NMR signals (*δ*_C_ 114.9, C–1'; 125.1, C–2'), the C_10_ unit was inferred. (Fig. 2). Additionally, the remained isobutyryl can only be attached at C–1.

**Table 1:** ^1^H (600 MHz) and ^13^C (150 MHz) NMR data of compounds **1 **and** 2** (*δ* in ppm and *J* in Hz)

	1		2	
position	^1^H ( *J*, Hz)	^13^C	^1^H (*J*, Hz)	^13^C
1		107.6		107.5
2		187.5		187.5
3		109.2		108.7
4		173.4		173.4
5		58.2		58.2
6		197.3		197.2
7		203.5		208.1
8	2.91 d (7.0)	49.4	3.87 m	43.4
9	2.07 dq (13.4, 6.4)	27.3	1.09 d (6.9)	16.9
10	0.94 s	22.9	1.74 m 1.34 overlap	27.7
11	0.95 s	23.1	0.90 m	12.3
1'	6.44 d (10.1)	114.9	6.44 d (10.1)	115.0
2'	5.45 d (10.1)	125.1	5.48 d (10.2)	125.1
3'		82.8		83.0
4'	1.45 s	28.9	1.46 s	29.1
5'	1.42 s	28.9	1.46 s	29.1
12	2.51 dd (13.9, 7.3)	38.7	2.51 dd (13.9, 7.4)	38.5
13	4.75 t (7.2)	119.2	4.78 t (7.2)	119.2
14		136.0		136.0
15	1.57 s	18.3	1.58 s	18.3
16	1.56 s	25.9	1.56 s	26.0
17	2.65 dd (13.8, 7.8)	38.7	2.67 dd (14.3, 7.4)	38.8
18	4.80 t (7.2)	119.2	4.78 t (7.2)	119.2
19		136.0		136.0
20	1.57 s	18.3	1.58 s	18.3
21	1.56 s	25.9	1.56 s	26.0

**2** was acquired as a pale brown oil. Combining the positive-ion HRESIMS peak at *m/z* 413.2690 [M + H]^+^ and ^13^C NMR revealed a molecular formula of C_26_H_36_O_4_. The IR spectrum implied absorption bands for hydroxyl (3440 cm^−1^) and carbonyl (1634 cm^−1^) functionalities. NMR spectra of **2** showed a close resemblance to those of **1** except for the replacements of the iso-butyl group at C–7 in **1** by a sce–butyl in **2**, respectively. The substituent at C–1 included a methine (*δ* 3.87, H–8), a methylene (*δ* 1.34, 1.74, H_2_–10), a methyl triplet (*δ* 0.90, H_3_–11), and a methyl doublet (*δ* 1.09, H_3_–9) (Table 1). In the COSY spectrum, the H_2_–10 was correlated with H_3_–11, and H–8 was correlated with H_3_–9. The HMBC spectrum showed that H_3_–11 was correlated with C–8, C–10, and H_3_–9 is correlated with C–7, C–8, which confirmed that the 2–methylbutyryl side chain was connected to C–1 (Fig. 2).

**Scheme 1:**
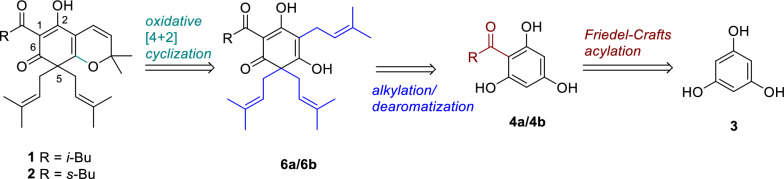
Retrosynthetic analysis of hyperprzewones A (**1**) and B (**2**)

As outlined in the retrosynthetic analysis (Scheme 1), we envisioned oxidizing triisoprenyl acylphloroglucinol via selectively oxidative [4 + 2] cyclization of intermediates **6a**/**6b**, thereby enabling the effective synthesis of the bicyclic natural products **1** and **2**. At the same time, we envisioned synthesizing intermediates **6a/6b** by first undergoing Friedel–Crafts alkylation and followed by dearomatization via intermediates **4a/4b**, or by simultaneously undergoing Friedel–Crafts alkylation and dearomatization using a certain method. The synthesis of intermediates **4a/4b** only requires phloroglucinol as the raw material, and can be carried out under conventional Friedel–Crafts acylation reaction conditions. This proposed route is more efficient, concise, and low-cost for synthesizing natural products **1** and **2** (Scheme 1).

**Scheme 2:**
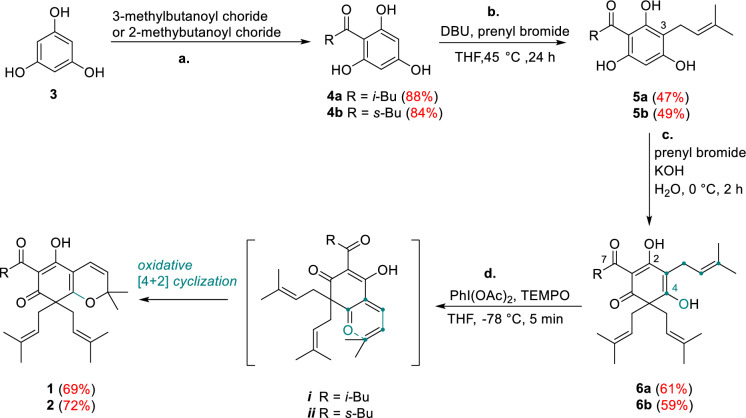
Total syntheses of **1** and** 2**. *Reagents and conditions*: **a**. 3-methylbutanoyl chloride or 2-methylbutanoyl chloride (1.2 eq.), AlCl_3_ (4.0 eq.), PhNO_2_, 65 °C, 34 h, **4a** (88%), **4b** (84%); **b**. prenyl bromide (2.0 eq.), DBU (2.0 eq.), THF, 45 °C, 24 h, **5a** (47%), **5b** (49%); **c**. prenyl bromide (2.0 eq.), KOH (2.0 eq.), H_2_O, 0 °C, 2 h, **6a** (61%), **6b** (59%); **d**. PhI(OAc)_2_ (2.0 eq.), TEMPO (1.2 eq.), THF, -78 °C, 5 min, **1** (69%), **2** (72%)

Our synthesis began with the Friedel–Crafts acylation of ordinary phloroglucinol **3** under the conditions of acyl chloride/AlCl_3_ in nitrobenzene to give the intermediate **4a** (88% yield) and **4b** (84% yield), respectively [15]. It was found that intermediate **6a** or **6b** can be simultaneously synthesized in one step from intermediate **4a** or **4b** through an alkylation/dearomatization cascade [16]. However, this method gave **6a**/**6b** in dissatisfactory yields (< 15%) in this work due to the instable and tautomeric nature of **4a**/**4b**. Therefore, we adopted stepwise alkylation and C-5 dearomatization as the ideal conditions to improve the yields of **6a**/**6b**. Intermediates **4a**/**4b** underwent an alkylation reaction with prenyl bromide using DBU as the base at 45 °C to synthesize intermediate **5a** (47% yield) and **5b** (49% yield), respectively [17]. Based on Lee’s protocol, attempt to extend the reaction time to 2 h in KOH aqueous solution at 0 °C gave **6a** (61% yield) and **6b** (59% yield) [16] (Scheme 2). Subsequently, oxidative [4 + 2] cyclization of **6a**/**6b** in the presence of PhI(OAc)_2_/TEMPO furnished natural products **1** in 69% yield and **2** in 72% yield, respectively. This transformation might be resulted from the *o*-quinone methide intermediates ***i*** and ***ii*** (Scheme 2), which formed by selective hydrogen abstraction of in situ generated TEMPO cation [18]. It is worth noting that the 4–phenol selectivity of this oxidative [4 + 2] cyclization could be well controlled due to the hydrogen bonding interactions between 2–phenol group with C–7 carbonyl group giving **1** or **2** as the single product. However, both products **1** and **2** were prone to enol-ketone tautomeric mixture (3:1), which were proved in ^1^H and ^13^C NMR spectral data as their natural samples. Finally, the syntheses of compounds **1** and **2** were completed in four steps, in overall yields of 17% (Scheme 2).

**Scheme3.**
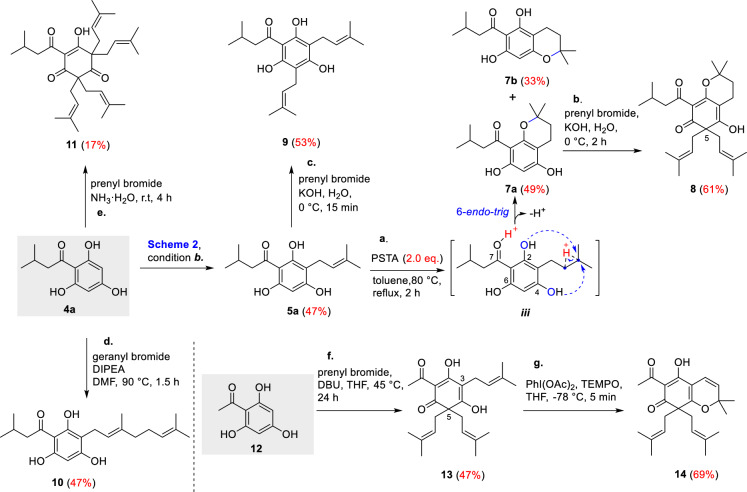
Syntheses of **1** and **2** derivatives. *Reagents and conditions*: **a**. PSTA (2.0 eq.), toluene, 80 °C, reflux, 2 h, **7a** (49%), **7b** (33%); **b**. prenyl bromide (2.0 eq.), KOH (2.0 eq.), H_2_O, 0 °C, 2 h, **8** (61%); **c**. prenyl bromide (2.0 eq.), KOH (2.0 eq.), H_2_O, 0 °C, 15 min, **9** (53%); **d**. geranyl bromide (2.0 eq.), DIPEA (2.0 eq.), DMF, 90 °C, 1.5 h, **10** (47%). **e**. prenyl bromide (2.0 eq.), NH_3_·H_2_O, r.t, 4 h, **11** (17%). **f**. prenyl bromide (2.0 eq.), DBU (2.0 eq.), THF, 45 °C, 24 h, **13** (47%); **g**. PhI(OAc)_2_ (2.0 eq.), TEMPO (1.2 eq.), THF, −78 °C, 5 min, **14** (69%)

Next, we prepared a series of derivatives based on **1** and **2**. Initially, treatment **5a** with PSTA (2.0 eq.) gave bicyclic compounds **7a** (49% yield) and **7b** (33% yield) through 6-*endo*-*trig* cyclization, respectively [19]. We believed that stoichiometric PTSA destroyed the hydrogen bonding interactions between 2– and 6–phenols with C–7 carbonyl enabling the corresponding cyclization through the proposed intermediate ***iii*** (Scheme 3), which promoted weaker nucleophilic 2-phenol to cyclization to **7a** as a major product [20]. A similar C–5 alkylation/dearomatization cascade of **7a** using prenyl bromide/KOH (aq.) generated **8** in 61% yield. It was found that C–4 phenol group ensured the dissolubility of such bicyclic compounds in KOH aqueous solution, as **7a** but **7b** was dissolvable. These observations provided a valuable reference for the synthesis of subsequent DIAPs natural products. Furthermore, we also transformed **5a** to **9** (53% yieid) by shortening the reaction time to 15 min in KOH aqueous solution (Scheme 3). Subsequently, **4a** underwent an alkylation reaction with geranyl bromide using DIPEA as the base at 90 °C to furnish derivative **10** in 47% yield [21]. Moreover, we synthesized tetraisopentenyl acylphloroglucinol derivative **11** albeit in 17% yield through C–3/C–5 alkylation/dearomatization cascade of **4a** under the conditions of prenyl bromide in an aqueous ammonia solution [22] (Scheme 3). Finally, a similar two-step synthesis of acylated derivative **14** (32% yield over two steps) was carried out using prenyl bromide/DBU and PhI(OAc)_2_/TEMPO from substrate **12**. Interestingly, **12** seemed more effective than **4a**/**4b** to generate the C–3 prenylated and C–5 di–prenylated product like **13** in 47% yield due to its less steric hindrance.

**Fig. 3:**
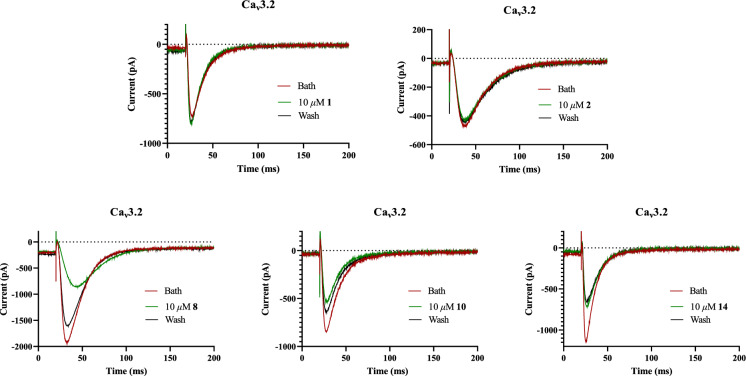
Ca_v_3.2 channel inhibitory activities of **1**, **2**, **8**, **10**, and** 14**

With all synthetic **1**/**2**-derivatives in hands, their Ca_v_3.2 channel inhibition were tested. As a result, natural products **1** and **2** exhibited no inhibitory effects on the Ca_v_3.2 channel at a concentration of 10 μM. However, their derivatives **8**, **10**, and **14** exhibited a good inhibitory effect on the Ca_v_3.2 channel (Fig. 3). At a concentration of 10 µM, the inhibitory rates of these derivatives were 55.29% (**8**), 36.55% (**10**), and 37.80% (**14**), respectively. Among them, derivative **8** showed the highest inhibitory activity, suggesting tautomeric enol-ketone moiety might be unfavorable for Ca_v_3.2 inhibition.

## Conclusions

In summary, two new natural products hyperprzewones A (**1**) and B (**2**) were isolated from *Hypericum przewalskii*. Structurally, these compounds were characterized by a dearomatized isoprenylated acylphloroglucinol core combined a functionalized cyclohexene skeleton. In addition, we have successfully achieved the total syntheses of **1** and **2** through Friedel–Crafts acylation, alkylation, dearomatization, and oxidative [4 + 2] cyclization in four steps. Biological evaluation of synthetic **1**/**2**-derivatives showed that **8**, **10**, and **14** exhibit inhibitory effects on the Ca_v_3.2 channel. Our finding provided a new direction for the development of Ca_v_3.2 channel drugs for the treatment of diseases such as neuropathic pain and epilepsy.

## Experimental section

### General experimental procedures

Optical rotations were measured on a Jasco P-1020 polarimeter. UV spectra were detected on a Shmadzu UV-2401PC spectrometer. IR spectra were determined on a Bruker FT-IR Tensor-27 infrared spectrophotometer with KBr disks. All 1D and 2D NMR spectra were recorded on Bruker AVANCE III 400 MHz and Bruker DRX–600 MHz spectrometers using TMS as an internal standard. Unless otherwise specified, chemical shifts (*δ*) were expressed in ppm with reference to the solvent signals. ESIMS and HRESIMS analysis were carried out on Waters Xevo TQS and Aglient G6230 TOF mass spectrometers, respectively. MCI gel (7–150 μm, Mitsubishi Chemical Corporation, Tokyo, Japan) were used for column chromatography. Synthetic field unless otherwise mentioned, all reactions were carried out under an argon atmosphere under anhydrous conditions, and all reagents were purchased from commercial suppliers without further purification. Silica gel (100–200, 200–300 mesh, Qingdao Marine Chemical Co., Ltd., People’s Republic of China). Fractions were monitored by TLC (GF 254, Qingdao Marine Chemical Co., Ltd.), and spots were visualized by heating silica gel plates sprayed with 10% H_2_SO_4_ in EtOH.

### Plant materials

The dried aerial parts of *Hypericum przewalskii* were collected at Aba Tibetan and Qiang Autonomous Prefecture, Sichuan Province, China, in September 2024. The plant was identified by Dr. Ruizhu Bai, Kunming Institute of Botany, Kunming, P. R. China. A voucher specimen was deposited with Kunming Institute of Botany with identification number (2024H01).

### Extraction and isolation

The air-dried aerial parts of *Hypericum przewalskii* (10.0 kg) were powered and extracted with MeOH (4 × 50 L) at room temperature for 24 h and filtered. After evaporating in vacuo, the crude extract (1.8 kg) was subjected to silica gel column chromatography and eluted with CHCl_3_ to afford a fraction (0.18 kg). This fraction was separated by MCI column chromatography (MeOH–H_2_O, 75:25 to 100:0) to provide seven sub-fractions (Fr. A1–A7). Fr. A3 (18.5 g) was separated into eight sub-fractions (Fr. A3.1–3.8) on silica gel column chromatography using a gradient of petroleum ether‒EtOAc (500:0 to 0:1). The obtained sub-fraction Fr. A3.1.5 (154.5 mg) was further fractionated by using an ODS column eluted with CH_3_OH–H_2_O (90:10, v/v) to give **1** (9.8 mg, t_R_ 15.7 min, CH_3_OH–H_2_O, 90%) and **2** (11.3 mg, t_R_ 14.3 min, CH_3_OH–H_2_O, 90%).

### Syntheses

***3-methyl-1-(2,4,6-trihydroxy-3-(3-methylbut-2-en-1-yl)phenyl)butan-1-one (5a)***

***2-methyl-1-(2,4,6-trihydroxy-3-(3-methylbut-2-en-1-yl)phenyl)butan-1-one (5b)***

To a solution of **4a**/**4b** (200 mg, 0.95 mmol, 1.0 eq.) and prenyl bromide (196 μL, 1.9 mmol, 2.0 eq.) in dry THF (3 mL) was added DBU (284 μL, 1.9 mmol, 2.0 eq.). The resultant suspension was heated at 45 °C for 24 h. The reaction mixture allowed to cool to room temperature, then acidified with 1 N HCl solution (1 mL) and extracted with EtOAc (3 × 10 mL). The combined extracts were washed with brine (3 × 10 mL), dried over Na_2_SO_4_, filtered and concentrated under reduced pressure. The residue was purified by flash chromatography on silica gel to give **5a** (124 mg, 47%) and **5b** (129 mg, 49%) as a yellow oil. Date for **5a**: R_f_ 0.6 (petrol/EtOAc, 1:1); ^1^H NMR (400 MHz, CD_3_OD) *δ* 5.89 (s, 1H), 5.21–5.11 (m, 1H), 3.18 (d, *J* = 7.1 Hz, 2H), 2.91 (d, *J* = 6.8 Hz, 2H), 2.21 (dt, *J* = 13.5, 6.8 Hz, 1H), 1.74 (s, 3H), 1.67–1.62 (m, 3H), 0.95 (d, *J* = 6.7 Hz, 6H). ^13^C NMR (150 MHz, CD_3_OD) *δ* 205.67, 163.65, 162.17, 159.96, 129.65, 123.17, 106.62, 104.06, 93.45, 52.40, 25.44, 24.59, 21.82, 20.78, 16.48; HRMS (C_16_H_22_O_4_, ESI): calculated [M-H]^−^ 277.1445, found 277.1446. Date for **5b**: R_f_ 0.6 (petrol/EtOAc, 1:1); ^1^H NMR (400 MHz, CD_3_OD) *δ* 5.88 (s, 1H), 5.15 (s, 1H), 3.87 (h, *J* = 6.7 Hz, 1H), 3.17 (d, *J* = 7.2 Hz, 2H), 1.86–1.76 (m, 1H), 1.76–1.72 (m, 3H), 1.67–1.62 (m, 3H), 1.36 (dt, *J* = 13.8, 7.1 Hz, 1H), 1.11 (d, *J* = 6.7 Hz, 3H), 0.90 (t, *J* = 7.4 Hz, 3H). ^13^C NMR (150 MHz, CD_3_OD) *δ* 210.10, 163.85, 162.01, 159.70, 129.62, 123.19, 106.70, 103.70, 93.54, 45.29, 26.81, 24.57, 20.81, 16.45, 15.87, 10.99; HRMS (C_16_H_22_O_4_, ESI): calculated [M-H]^−^ 277.1445, found 277.1446.

***3,5-dihydroxy-4,6,6-tris(3-methylbut-2-en-1-yl)-2-(3-methylbutanoyl)cyclohexa-2,4-dien-1-one (6a)***

***3,5-dihydroxy-4,6,6-tris(3-methylbut-2-en-1-yl)-2-(2-methylbutanoyl)cyclohexa-2,4-dien-1-one (6b)***

To a solution of **5a**/**5b** (100 mg, 0.36 mmol, 1.0 eq.) in H_2_O (2 mL) under a nitrogen atmosphere at 0 °C was added KOH (40 mg, 0.72 mmol, 2.0 eq.) in one portion, then prenyl bromide (70 μL, 0.72 mmol, 2.0 eq.) was added dropwise over 20 min. The reaction mixture was stirred at 0 °C for a further 2 h, during which time a thick orange precipitate was formed. The reaction mixture was then acidified with 1 N HCl solution (1 mL) and then extracted with EtOAc (3 × 10 mL). The combined extracts were washed with brine (3 × 10 mL), dried over Na_2_SO_4_, filtered and concentrated under reduced pressure. The residue was purified by flash chromatography on silica gel (petrol/EtOAc, 60:1 → 30:1) to give **6a** (90 mg, 61%) as a pale yellow oil and **6b** (88 mg, 59%) as a white oil. Date for **6a**: R_f_ 0.5 (petrol/EtOAc, 2:1); ^1^H NMR (400 MHz, CD_3_OD) *δ* 5.25–5.09 (m, 1H), 4.85–4.73 (m, 2H), 3.20 (d, *J* = 7.3 Hz, 2H), 2.92 (d, *J* = 7.0 Hz, 2H), 2.67–2.61 (m, 2H), 2.51 (dd, *J* = 13.8, 7.8 Hz, 2H), 2.19–2.09 (m, 1H), 1.79 (d, *J* = 5.0 Hz, 6H), 1.56 (s, 12H), 0.98–0.95 (m, 6H). ^13^C NMR (150 MHz, CD_3_OD) *δ* 203.97, 198.19, 191.00, 174.44, 135.67, 132.59, 123.22, 119.21, 112.39, 109.36, 58.7, 50.10, 38.77, 27.05, 26.03, 23.11, 21.61, 17.94; HRMS (C_26_H_38_O_4_, ESI): calculated [M-H]^−^ 413.2697, found 413.2703. Date for **6b**: R_f_ 0.5 (petrol/EtOAc, 2:1); ^1^H NMR (400 MHz, CD_3_OD) *δ* 5.01 (d, *J* = 7.2 Hz, 1H), 4.77 (d, *J* = 7.4 Hz, 2H), 3.89 (q, *J* = 7.0 Hz, 1H), 3.10 (d, *J* = 7.0 Hz, 2H), 2.66–2.51 (m, 4H), 1.73 (d, *J* = 5.3 Hz, 4H), 1.67 (s, 3H), 1.58–1.53 (m, 12H), 1.38–1.33 (m, 1H), 1.08 (d, *J* = 6.7 Hz, 3H), 0.90 (d, *J* = 7.4 Hz, 3H). ^13^C NMR (150 MHz, CD_3_OD) *δ* 208.30, 198.02, 191.21, 174.23, 135.63, 132.56, 123.24, 119.20, 112.35, 108.82, 58.73, 43.72, 39.05, 38.78, 27.7, 26.05, 21.65, 18.10, 16.99, 12.36; HRMS (C_26_H_38_O_4_, ESI): calculated [M-H]^−^ 413.2697, found 413.2707.

***hyperprzewone A (1) and hyperprzewone B (2)***

To a solution of **6a**/**6b** (50 mg, 0.12 mmol, 1.0 eq.) in dry THF (2 mL) at −78 °C under a nitrogen atmosphere was added TEMPO (38 mg, 0.24 mmol, 2.0 eq.) followed by PhI(OAc)_2_ (45 mg, 0.14 mmol, 1.2 eq.). The reaction mixture was stirred at −78 °C for 5 min, then allowed to warm to room temperature over 30 min. The reaction mixture was quenched with H_2_O (1 mL), then extracted with EtOAc (3 × 10 mL). The combined organics were dried over Na_2_SO_4_, filtered and concentrated under reduced pressure. The residue was then purified by flash chromatography on silica gel (petrol/EtOAc, 100:1 → 40:1) to give **1** (33 mg, 69%) and **2** (36 mg, 72%) as a brown oil. Date for **1**: R_f_ 0.6 (petrol/EtOAc, 10:1); ^1^H NMR (400 MHz, CD_3_OD) *δ* 6.44 (d, *J* = 10.1 Hz, 1H), 5.45 (dd, *J* = 22.8, 10.1 Hz, 1H), 4.80–4.68 (m, 2H), 2.91 (d, *J* = 7.0 Hz, 2H), 2.65 (dd, *J* = 13.8, 7.8 Hz, 2H), 2.51 (dd, *J* = 13.9, 7.3 Hz, 2H), 2.07 (dq, *J* = 13.4, 6.4 Hz, 1H), 1.57 (d, *J* = 8.8 Hz, 12H), 1.44 (d, *J* = 11.8 Hz, 6H), 0.95 (d, *J* = 6.8, , 6H). ^13^C NMR (150 MHz, CD_3_OD) *δ* 203.52, 197.30, 187.49, 173.38, 136.00, 125.06, 119.16, 118.66, 114.98, 109.27, 107.61, 82.85, 58.24, 49.39, 38.66, 37.35, 29.08, 27.27, 25.98, 22.96, 18.31; HRMS (C_26_H_36_O_4_, ESI): calculated [M-H]^−^ 411.2541, found 411.2549; [α] −1.17 (*c* 0.12, MeOH); UV (MeOH)*λ*_max_ (log *ε*) 195 (4.34), 226 (3.80), 267 (3.97), 326 (3.59), 337 (3.60) nm; IR (KBr)*v*_max_ 3433, 2962, 2928, 2871, 1654, 1634, 1527, 1466, 1381. Date for **2**: R_f_ 0.6 (petrol/EtOAc, 10:1); ^1^H NMR (400 MHz, CD_3_OD) *δ* 6.44 (d, *J* = 10.1 Hz, 1H), 5.48 (d, *J* = 10.2 Hz, 1H), 4.82–4.77 (m, 2H), 3.95–3.79 (m, 1H), 2.67 (dt, *J* = 14.3, 7.4 Hz, 2H), 2.51 (dd, *J* = 13.9, 7.4 Hz, 2H), 1.76–1.70 (m, 1H), 1.57 (d, *J* = 9.0 Hz, 12H), 1.46 (s, 6H), 1.34 (s, 1H), 1.09 (d, *J* = 6.9 Hz, 3H), 0.90 (d, *J* = 7.5 Hz, 3H). ^13^C NMR (150 MHz, CD_3_OD) *δ* 208.09, 197.18, 187.54, 173.34, 136.00, 125.07, 119.24, 114.99, 108.70, 107.49, 82.95, 58.43, 43.42, 38.84, 38.51, 29.07, 27.71, 26.01, 18.29, 16.94, 12.33; HRMS (C_26_H_36_O_4_, ESI): calculated [M+H]^+^ 413.2686, found 413.2690; [α] −0.83 (c 0.17, MeOH); UV (MeOH)*λ*_max_ (log *ε*) 195 (4.01), 228 (3.36), 267 (3.57), 327 (3.04), 351 (3.08) nm; IR (KBr)*v*_max_ 3440, 2969, 2928, 2876, 1654, 1634, 1526, 1465, 1380.

***1-(5,7-dihydroxy-2,2-dimethylchroman-8-yl)-3-methylbutan-1-one (7a)***

***1-(5,7-dihydroxy-2,2-dimethylchroman-6-yl)-3-methylbutan-1-one (7b)***

To a solution of **5a** (100 mg, 0.36 mmol, 1.0 eq.) in toluene (3 mL) at room temperature was added PSTA (124 mg, 0.72 mmol, 2.0 eq.). The reaction mixture was stirred at room temperature for 2 h. The reaction mixture was quenched with H_2_O (1 mL), then extracted with EtOAc (3 × 10 mL). The combined organics were dried over Na_2_SO_4_, filtered and concentrated under reduced pressure. The residue was then purified by flash chromatography on silica gel (petrol/EtOAc, 40:1 → 15:1) to give **7a** (49 mg 49%) as a white oil and on silica gel (petrol/EtOAc, 40:1 → 20:1) to give **7b** (33 mg 33%) as a yellow oil. Date for **7a**: R_f_ 0.4 (petrol/EtOAc, 4:1); ^1^H NMR (400 MHz, CDCl_3_) *δ* 13.92 (s, 1H), 5.93 (s, 1H), 2.90 (d, *J* = 7.0 Hz, 2H), 2.59 (t, *J* = 6.8 Hz, 2H), 2.21 (dq, *J* = 13.4, 6.7 Hz, 1H), 1.80 (t, *J* = 6.8 Hz, 2H), 1.40 (s, 6H), 0.97 (d, *J* = 6.6 Hz, 6H). ^13^C NMR (600 MHz, CDCl_3_) *δ* 206.01, 165.07, 159.94, 157.14, 106.32, 99.36, 95.34, 76.04, 53.40, 31.51, 26.77, 25.39, 22.77, 16.35; HRMS (C_16_H_22_O_4_, ESI): calculated [M-H]^−^ 277.1445, found 277.1442. Date for **7b**: R_f_ 0.5 (petrol/EtOAc, 4:1); ^1^H NMR (400 MHz, CDCl_3_) *δ* 13.57 (s, 1H), 6.43 (s, 1H), 5.72 (s, 1H), 2.93 (d, *J* = 6.8 Hz, 2H), 2.58 (t, *J* = 6.8 Hz, 2H), 2.26 (dp, *J* = 13.4, 6.7 Hz, 1H), 1.78 (t, *J* = 6.8 Hz, 2H), 1.32 (s, 6H), 0.97 (d, *J* = 6.7 Hz, 6H). ^13^C NMR (150 MHz, CDCl_3_) *δ* 205.87, 164.00, 160.40, 158.01, 104.46, 101.74, 95.72, 76.13, 52.91, 32.31, 26.89, 25.62, 23.04, 16.30; HRMS (C_16_H_22_O_4_, ESI): calculated [M-H]^−^ 277.1445, found 277.1452.

***5-hydroxy-2,2-dimethyl-6,6-bis(3-methylbut-2-en-1-yl)-8-(3-methylbutanoyl)-2,3,4,6-tetrahydro-7H-chromen-7-one (8)***

To a solution of **7a** (40 mg, 0.14 mmol, 1.0 eq.) in H_2_O (1.5 mL) under a nitrogen atmosphere at 0 °C was added KOH (16 mg, 0.28 mmol, 2.0 eq.) in one portion, then prenyl bromide (27 μL, 0.28 mmol, 2.0 eq.) was added dropwise over 20 min. The resultant suspension was stirred at 0 °C for a further 2 h, then reaction mixture was acidified with 1 N HCl solution (1 mL) and extracted with EtOAc (3 × 10 mL). The combined extracts were washed with brine (3 × 10), dried over Na_2_SO_4_, filtered and concentrated under reduced pressure. The residue was purified by flash chromatography on silica gel (petrol/EtOAC, 60:1 → 35:1) to give **8** (34 mg, 61%) as a pale yellow oil. Date for **8**: R_f_ 0.6 (petrol/EtOAc, 4:1); ^1^H NMR (400 MHz, CD_3_OD) *δ* 4.78–4.70 (m, 2H), 2.78 (d, *J* = 7.3 Hz, 2H), 2.69–2.55 (m, 4H), 2.34 (t, *J* = 6.8 Hz, 2H), 2.10 (dt, *J* = 13.6, 6.8 Hz, 1H), 1.78 (t, *J* = 6.8 Hz, 2H), 1.60–1.46 (m, 12H), 1.40 (s, 6H), 0.96 (d, *J* = 6.7 Hz, 6H). ^13^C NMR (150 MHz, CD_3_OD) *δ* 201.38, 198.09, 196.61, 166.61, 136.01, 119.17, 108.78, 107.39, 80.25, 61.01, 49.43, 39.67, 32.52, 28.58, 26.66, 26.03, 22.87, 17.98, 17.32; HRMS (C_26_H_38_O_4_, ESI): calculated [M-H]^−^ 413.2770, found 413.2697.

***(E)-1-(3-(3,7-dimethylocta-2,6-dien-1-yl)-2,4,6-trihydroxyphenyl)-3-methylbutan-1-one (10)***

To a mixture solution of **4a** (100 mg, 0.48 mmol, 1.0 eq.) and geranyl bromide (190 μL, 0.96 mmol, 2.0 eq.) in DMF (2 mL) was added DIPEA (171 μL, 0.96 mmol, 2.0 eq.). The resultant suspension was heated at 80 °C for 3 h, then gradually warmed to room temperature. The reaction mixture was quenched with H_2_O (1 mL) and extracted with EtOAc (3 × 10 mL). The combined organic extracts were dried over Na_2_SO_4_, filtered and concentrated under reduced pressure. The residue was then purified by flash chromatography on silica gel (petrol/EtOAc, 20:1 → 5:1) to give **10** (78 mg 47%) as a yellow oil. Date for **10**: R_f_ 0.4 (petrol/EtOAc, 2:1); ^1^H NMR (400 MHz, CD_3_OD) *δ* 5.88 (s, 1H), 5.17 (dt, *J* = 6.1, 4.3 Hz, 1H), 5.05 (dt, *J* = 7.3, 1.5 Hz, 1H), 3.18 (d, *J* = 7.1 Hz, 2H), 2.91 (d, *J* = 6.8 Hz, 2H), 2.21 (dt, *J* = 13.1, 6.6 Hz, 1H), 2.04 (q, *J* = 7.4 Hz, 2H), 1.93 (t, *J* = 7.5 Hz, 2H), 1.75–1.72 (m, 3H), 1.63– 1.56 (m, 3H), 1.55 (s, 3H), 0.95 (d, *J* = 6.7 Hz, 6H). ^13^C NMR (150 MHz, CD_3_OD) *δ* 207.03, 165.37, 163.87, 161.63, 134.93, 130.94, 125.80, 124.94, 108.35, 105.75, 95.12, 53.80, 40.93, 28.01, 27.13, 26.13, 23.50, 22.09, 17.71, 16.48; HRMS (C_21_H_30_O_4_, ESI): calculated [M + H]^+^ 347.2217, found 347.2217.

***5-hydroxy-2,2,6,6tetrakis(3-methylbut-2-en-1-yl)-4-(3-methylbutanoyl)cyclohex-4-ene-1,3-dione (11)***

To a solution of **4a** (40 mg, 0.19 mmol, 1.0 eq.) and prenyl bromide (45 μL, 0.38 mmol, 2.0 eq.) in NH_3_·H_2_O (1.5 mL). The resultant suspension was stirred for 4 h at room temperature, then acidified with 1 N HCl solution (1.5 mL) and extracted with EtOAc (3 × 10 mL). The combined extracts were washed with brine (3 × 10 mL), dried over Na_2_SO_4_, filtered and concentrated under reduced pressure. The residue was purified by flash chromatography on silica gel (petrol/EtOAc, 100:1 → 60:1) to give **11** (13 mg, 17%) as a white oil. Data for **11**: R_f_ 0.4 (petrol/EtOAc, 20:1); ^1^H NMR (400 MHz, CDCl_3_) *δ* 4.90 (s, 4H), 2.81 (s, 2H), 2.65 (dd, *J* = 13.8, 8.0 Hz, 2H), 2.53 (dt, *J* = 14.9, 7.9 Hz, 4H), 2.30 (dd, *J* = 14.6, 6.1 Hz, 2H), 2.23–2.14 (m, 1H), 1.63–1.54 (m, 24H), 0.99 (d, *J* = 6.7 Hz, 6H); ^13^C NMR (150 MHz, CDCl_3_) *δ* 207.55, 203.65, 197.41, 194.73, 136.16, 134.60, 118.89, 118.26, 113.57, 65.53, 60.94, 47.65, 36.82, 33.85, 25.96, 25.85, 25.77, 22.69, 17.88, 17.84; HRMS (C_31_H_46_O_4_, ESI): calculated [M + H]^+^ 483.3469, found 483.3472.

*6-acetyl-5-hydroxy-2,2-dimethyl-8,8-bis(3-methylbut-2-en-1-yl)-2,8-dihydro-7H-chromen-7-one (14)* To a solution of **13** (200 mg, 0.54 mmol, 1.0 eq.) in dry THF (4 mL) at −78 °C under a nitrogen atmosphere was added TEMPO (169 mg, 1.08 mmol, 2.0 eq.) followed by PhI(OAc)_2_ (209 mg, 0.65 mmol, 1.2 eq.). The reaction mixture was stirred at −78 °C for 5 min, then allowed to warm to room temperature over 30 min. The reaction mixture was quenched with H_2_O (2 mL), then extracted with EtOAc (3 × 30 mL). The combined organics were dried over Na_2_SO_4_, filtered and concentrated under reduced pressure. The residue was then purified by flash chromatography on silica gel (petrol/EtOAc, 80:1 → 30:1) to give **14** (110 mg, 64%) as a yellow oil. Date for **14**: R_f_ 0.4 (petrol/EtOAc, 10:1); ^1^H NMR (400 MHz, CDCl_3_) *δ* 6.48 (dd, *J* = 29.2, 10.0 Hz, 1H), 5.32 (dd, *J* = 14.7, 10.0 Hz, 1H), 4.77 (t, *J* = 7.1 Hz, 2H), 2.70 (d, *J* = 8.3 Hz, 2H), 2.58 (s, 3H), 2.48 (dd, *J* = 13.9, 7.6 Hz, 2H), 1.62 (d, *J* = 1.0 Hz, 12H), 1.41 (d, *J* = 13.9 Hz, 6H). ^13^C NMR (150 MHz, CDCl_3_) *δ* 199.48, 195.47, 185.85, 172.21, 134.60, 123.10, 117.90, 114.31, 108.20, 105.70, 81.03, 56.80, 37.34, 28.50, 25.50, 17.91; HRMS (C_23_H_30_O_4_, ESI): calculated [M-H]^−^ 369.2071, found 369.2075.

### Cell preparation and expression

Human embryonic kidney (HEK) 293 T cells were grown in DMEM (cytiva) plus 10% newborn calf serum (Gibco) and penicillin (100 U/mL)/streptomycin (0.1 mg/mL) (Biological Industries). HEK 293 T cells were transiently co-transfected with pCDNA3.1-Ca_v_3.2 and EGFP plasmids together using Lipofectamine™3000 (invitrogen) and used in 48 h.

## Supplementary Information

Additional file1 (PDF 6092 KB)

## Data Availability

The datasets used or analyzed during the current study are available from the corresponding author on reasonable request.

## References

[1] Singh IP, Bharate SB. Phloroglucinol compounds of natural origin. Nat Prod Rep. 2006;23:558–91. 16874390 10.1039/b600518g

[2] Ciochina R, Grossman RB. Polycyclic polyprenylated acylphloroglucinols. Chem Rev. 2006;106:3963–86. 16967926 10.1021/cr0500582

[3] Yang XW, Grossman RB, Xu G. Research progress of polycyclic polyprenylated acylphloroglucinols. Chem Rev. 2018;118:3508–58. 29461053 10.1021/acs.chemrev.7b00551

[4] Wu SB, Long C, Kennelly EJ. Structural diversity and bioactivities of natural benzophenones. Nat Prod Rep. 2014;31:1158–74. 24972079 10.1039/c4np00027g

[5] Guan X, Wang H, Zhang W, Xie Z. Asymmetric total synthesis of (+)-hyperbeanol A. Org Lett. 2025;27:8–13. 39696798 10.1021/acs.orglett.4c02930

[6] Jiang NN, Gar-Lee YG, Li P, Ye YS, Gomes AJ, Hin-Fai KF, et al. Discovery of dearomatized isoprenylated acylphloroglucinols with colon tumor suppressive activities in mice via inhibiting NFκB-FAT1-PDCD4 signaling activation. Eur J Med Chem. 2022;239:114532. 35749988 10.1016/j.ejmech.2022.114532

[7] Tao Y, Reisenauer K, Taube JH, Romo D. Total synthesis and anticancer activity of (+)-hypercalin C and congeners. Angew Chem Int Ed Engl. 2019;58:2734–8. 30600887 10.1002/anie.201812909 PMC6438696

[8] Cheong E, Shin HS. T-Type Ca^2+^ channels in normal and abnormal brain functions. Physiol Rev. 2013;93:961–92. 23899559 10.1152/physrev.00010.2012

[9] Kim D, Song I, Keum S, Lee T, Jeong MJ, Kim SS, et al. Lack of the burst firing of thalamocortical relay neurons and resistance to absence seizures in mice lacking α_1G_ T-type Ca^2+^ channels. Neuron. 2001;31:35–45. 11498049 10.1016/s0896-6273(01)00343-9

[10] Zamponi GW, Striessnig J, Koschak A, Dolphin AC. The physiology, pathology, and pharmacology of voltage-gated calcium channels and their future therapeutic potential. Pharmacol Rev. 2015;67:821–70. 26362469 10.1124/pr.114.009654 PMC4630564

[11] Tadayonnejad R, Anderson D, Molineux ML, Mehaffey WH, Jayasuriya K, Turner RW. Rebound discharge in deep cerebellar nuclear neurons in vitro. Cerebellum. 2010;9:352–74. 20396983 10.1007/s12311-010-0168-7 PMC2949560

[12] Ye YS, Liu R, Jiang NN, Li SY, Nian Y, Xu G. Caged polycyclic polyprenylated acylphloroglucinols as Cav3.2 low voltage-gated Ca^2+^ channel inhibitors from *Hypericum curvisepalum*. Chem Commun. 2022;58:13135–8. 10.1039/d2cc05396a 36349731

[13] Hu YL, Dong D, Zhao JJ, Hu K, Kong LM, Hu YX, et al. 5-Methylated polyprenylated acylphloroglucinol derivatives as low-voltage-gated Ca^2+^ channel inhibitors. Org Chem Front. 2025;12:2375–81.

[14] Ye YS, Wu M, Jiang NN, Lao YZ, Fu WW, Liu X, et al. Dearomatized isoprenylated acylphloroglucinol derivatives with potential antitumor activities from *Hypericum henryi*. Nat Prod Bioprospect. 2020;10:1–11. 32016770 10.1007/s13659-019-00229-w PMC7046846

[15] Chauthe SK, Bharate SB, Sabde S, Mitra D, Bhutani KK, Singh IP. Biomimetic synthesis and anti-HIV activity of dimeric phloroglucinols. Bioorg Med Chem. 2010;18:2029–36. 20137956 10.1016/j.bmc.2010.01.023

[16] Nakagawa G, Wu PC, Bastow KF, Yang SC, Yu SL, Chen HY, Lin JC, Goto M, Morris-Natschke SL, Yang PC, Lee KH. Antitumor agents 283. Further elaboration of desmosdumot in C analogs as potent antitumor agents: activation of spindle assembly checkpoint as possible mode of action. Bioorg Med Chem. 2011;19:1816–22. 21296579 10.1016/j.bmc.2011.01.001 PMC3064560

[17] Mzozoyana V, Van H, Fanie R. Synthesis of 3-geranyl- and 3-prenyl-2,4,6-trihydroxybenzophenone. Synth Commun. 2017;47:599–603.

[18] George JH, Hesse MD, Baldwin JE, Adlington RM. Biomimetic synthesis of polycyclic polyprenylated acylphloroglucinol natural products isolated from *Hypericum papuanum*. Org Lett. 2010;12:3532–5. 20590087 10.1021/ol101380a

[19] Kuhnke J, Bohlmann F. Synthesis of naturally occurring phloroglucinol derivatives. Tetrahedron. 1985;26:3955–8.

[20] Yuan ST, Peng YP, Xia D, Jiang J, Li k, Li DS, et al. De novo syntheses of coumaronochromones: application in total synthesis of cristatone II. J Org Chem. 2025;90:7877–87. 40440713 10.1021/acs.joc.5c00817

[21] Wang X, Lee YR. Efficient synthesis of polycycles bearing prenylated, geranylated, and farnesylated citrans: application to 3′-prenylrubranine and petiolin D regioisomer. Tetrahedron. 2011;67:9179–84.

[22] Elizabeth T, Roland A, Matt T, Kay C, Grisha P, Dharahana R, et al. The synthesis and anticancer effects of a range of natural and unnatural hop β-acids on breast cancer cells. Phytochemistry. 2012;5:144–9.

