# Supplementary material for: Isolation, total synthesis, and biological evaluation of dearomatized isoprenylated acylphloroglucinols from Hypericum przewalskii
Source: Nat Prod Bioprospect. 2026 Apr 1;16(1):47. doi: 10.1007/s13659-026-00626-y (PMC13043971; doi:10.1007/s13659-026-00626-y)

Supporting Information

Isolation, Total Synthesis, and Biological Evaluation of Dearomatized Isoprenylated Acylphloroglucinols from *Hypericum przewalskii*

Yong Li[1,†], Fei-Fei Xiong[2,3,†], Xiao-Yang Sun[2,3,†], Xing-Ren Li[2], Dao-Feng Chen[1,4,*], Gang Xu[1,2,*], Yin Nian[2,*] and Li-Dong Shao[1,*]

[1]Yunnan Key Laboratory of Southern Medicinal Utilization, School of Chinese Materia Medica, Yunnan University of Chinese Medicine, Kunming 650500, China.

[2]Key Laboratory of Phytochemistry and Natural Medicines, Kunming Institute of Botany, Chinese Academy of Sciences, Kunming 650201, People's Republic of China.

[3]University of Chinese Academy of Sciences, Beijing 100049, China.

[4]School of Pharmacy, Institutes of Integrative Medicine, Fudan University, Shanghai 201203, China.

---

† These authors were contributed equally.

* Correspondence:

Li-Dong Shao, E-mail: shaolidong@ynucm.edu.cn

Yin Nian, E-mail: nianyin@mail.kib.ac.cn

Gang Xu, E-mail: xugang008@mail.kib.ac.cn

Dao-Feng Chen, E-mail: dfchen@shmu.edu.cn

Table of Contents

1. General Information 1

2. Experimental procedures 1

- **2. 1 Synthesis of 4a and 4b** 1
- **2.2 Synthesis of 5a and 5b** 1
- **2.3 Synthesis of 6a and 6b** 2
- **2.4 Synthesis of 1 and 2** 3
- **2.5 Synthesis of 7a and 7b** 4
- **2.6 Synthesis of 8** 4
- **2.7 Synthesis of 9** 5
- **2.8 Synthesis of 10** 5
- **2.9 Synthesis of 11** 6
- **2.10 Synthesis of 12** 6
- **2.11 Synthesis of 13** 7
- **2.12 Synthesis of 14** 7

3. Spectral date scans of all compounds 9

- **Fig. S1.** ^{1}H NMR Spectrum of Compound **4a** (400 MHz, CD_3OD) 9

Fig. S2. ^{13}C NMR Spectrum of Compound **4a** (150 MHz, CD_3OD)..9
Fig. S3. ^{1}H NMR Spectrum of Compound **4b** (400 MHz, CD_3OD)..10
Fig. S4. ^{13}C NMR Spectrum of Compound **4b** (150 MHz, CD_3OD)..10
Fig. S5. ^{1}H NMR Spectrum of Compound **5a** (400 MHz, CD_3OD)..11
Fig. S6. ^{13}C NMR Spectrum of Compound **5a** (150 MHz, CD_3OD)..11
Fig. S7. ^{1}H NMR Spectrum of Compound **5b** (400 MHz, CD_3OD)..12
Fig. S8. ^{13}C NMR Spectrum of Compound **5b** (150 MHz, CD_3OD)..12
Fig. S9. ^{1}H NMR Spectrum of Compound **6a** (400 MHz, CD_3OD)..13
Fig. S10. ^{13}C NMR Spectrum of Compound **6a** (150 MHz, CD_3OD)..13
Fig. S11. ^{1}H NMR Spectrum of Compound **6b** (400 MHz, CD_3OD)..14
Fig. S12. ^{13}C NMR Spectrum of Compound **6b** (150 MHz, CD_3OD)..14
Fig. S13. ^{1}H NMR Spectrum of Compound **1** (400 MHz, CD_3OD)..15
Fig. S14. ^{13}C NMR Spectrum of Compound **1** (150 MHz, CD_3OD)..15
Fig. S15. ^{1}H NMR Spectrum of Compound **1** (400 MHz, $CDCl_3$)..16
Fig. S16. ^{13}C NMR Spectrum of Compound **1** (150 MHz, $CDCl_3$)..16
Fig. S17. Dept135 Spectrum of Compound **1** (150 MHz, CD_3OD)..17
Fig. S18. Cosy Spectrum of Compound **1** (150 MHz, CD_3OD)..17
Fig. S19. HMBC Spectrum of Compound **1** (150 MHz, CD_3OD)..18
Fig. S20. HSQC Spectrum of Compound **1** (150 MHz, CD_3OD)..18
Fig. S21. Noesy Spectrum of Compound **1** (150 MHz, CD_3OD)..19
Fig. S22. ^{1}H NMR Spectrum of Compound **2** (400 MHz, CD_3OD)..20
Fig. S23. ^{13}C NMR Spectrum of Compound **2** (150 MHz, CD_3OD)..20
Fig. S24. ^{1}H NMR Spectrum of Compound **2** (400 MHz, $CDCl_3$)..21
Fig. S25. ^{13}C NMR Spectrum of Compound **2** (150 MHz, $CDCl_3$)..21
Fig. S26. Dept135 Spectrum of Compound **2** (150 MHz, CD_3OD)..22
Fig. S27. HMBC Spectrum of Compound 2 (150 MHz, CD_3OD)..22
Fig. S28. HSQC Spectrum of Compound **2** (150 MHz, CD_3OD)..23
Fig. S29. Cosy Spectrum of Compound **2** (150 MHz, CD_3OD)..23
Fig. S30. ^{1}H NMR Spectrum of Compound **7b** (400 MHz, $CDCl_3$)..24
Fig. S31. ^{13}C NMR Spectrum of Compound 7b (150 MHz, $CDCl_3$)..24
Fig. S32. HMBC Spectrum of Compound **7b** (150 MHz, $CDCl_3$)..25
Fig. S33. HSQC Spectrum of Compound **7b** (150 MHz, $CDCl_3$)..25
Fig. S34. ^{1}H NMR Spectrum of Compound **7a** (400 MHz, $CDCl_3$)..26
Fig. S35. ^{13}C NMR Spectrum of Compound **7a** (150 MHz, $CDCl_3$)..26
Fig. S36. ^{1}H NMR Spectrum of Compound **8** (400 MHz, CD_3OD)..27
Fig. S37. ^{13}C NMR Spectrum of Compound **8** (150 MHz, CD_3OD)..27
Fig. S38. HMBC Spectrum of Compound **8** (150 MHz, CD_3OD)..28
Fig. S39. HSQC Spectrum of Compound **8** (150 MHz, CD_3OD)..28
Fig. S40. ^{1}H NMR Spectrum of Compound **9** (400 MHz, CD_3OD)..29
Fig. S41. ^{13}C NMR Spectrum of Compound **9** (150 MHz, CD_3OD)..29
Fig. S42. ^{1}H NMR Spectrum of Compound **10** (400 MHz, CD_3OD)..30
Fig. S43. ^{13}C NMR Spectrum of Compound **10** (150 MHz, CD_3OD)..30
Fig. S44. ^{1}H NMR Spectrum of Compound **11** (400 MHz, $CDCl_3$)..31
Fig. S45. ^{13}C NMR Spectrum of Compound **11** (150 MHz, $CDCl_3$)..31

Fig. S46. ^{1}H NMR Spectrum of Compound **12** (400 MHz, CD_3OD) 32
Fig. S47. ^{13}C NMR Spectrum of Compound **12** (600 MHz, CD_3OD) 32
Fig. S48. ^{1}H NMR Spectrum of Compound **13** (400 MHz, CD_3OD) 33
Fig. S49. ^{13}C NMR Spectrum of Compound **13** (150 MHz, CD_3OD) 33
Fig. S50. ^{1}H NMR Spectrum of Compound **14** (400 MHz, $CDCl_3$) 34
Fig. S51. ^{13}C NMR Spectrum of Compound **14** (150 MHz, $CDCl_3$) 34
Fig. S52. HMBC Spectrum of Compound **14** (150 MHz, $CDCl_3$) 35
Fig. S53. HSQC Spectrum of Compound **14** (150 MHz, $CDCl_3$) 35
4. Comparison of NMR data of natural 1 and synthetic 1 36
Comparison of ^{1}H NMR spectrum of natural **1** and synthetic **1** 37
Comparison of ^{13}C NMR spectrum of natural **1** and synthetic **1** 37
5. Comparison of NMR data of natural 2 and synthetic 2 38
Comparison of ^{1}H NMR spectrum of natural **2** and synthetic **2** 39
Comparison of ^{13}C NMR spectrum of natural **2** and synthetic **2** 39
6. HRESI-MS Spectra of all Compounds 40
7. UV and IR Spectra of Compounds 1 and 2 47
8. Primary Activity Screening 48

1. General Information

Optical rotations were measured on a Jasco P-1020 polarimeter. UV spectra were detected on a Shmadzu UV-2401PC spectrometer. IR spectra were determined on a Bruker FT-IR Tensor-27 infrared spectrophotometer with KBr disks. NMR spectra were recorded on Bruker AVANCE III 400MHz (^{1}H NMR) spectrometer and Bruker DRX 600MHz (^{13}C NMR) spectrometer, and calibrated using residual undeuterated solvent as an internal reference ($CDCl_3$, δ 7.26 ppm ^{1}H NMR, δ 77.0 ppm ^{13}C NMR; Methanol-d_4, δ 4.87 ppm ^{1}H NMR, δ 49.0 ppm ^{13}C NMR;).ESIMS and HRESIMS analysis were carried out on Waters Xevo TQS and Aglient G6230 TOF mass spectrometers, respectively. MCI gel (7 – 150 μm, Mitsubishi Chemical Corporation, Tokyo, Japan) were used for column chromatography. Synthetic field unless otherwise mentioned, all reactions were carried out under an argon atmosphere under anhydrous conditions, and all reagents were purchased from commercial suppliers without further purification. Silica gel (100 – 200, 200 – 300 mesh, Qingdao Marine Chemical Co., Ltd., People's Republic of China). Fractions were monitored by TLC (GF 254, Qingdao Marine Chemical Co., Ltd.), and spots were visualized by heating silica gel plates sprayed with 10% H_2SO_4 in EtOH.

2. Experimental procedures

2. 1 Synthesis of 4a and 4b

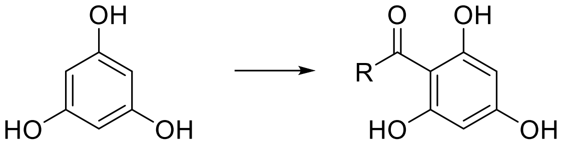

3

4a R = *i*-Bu
4b R = *s*-Bu

Phloroglucinol (1.0 g, 7.93 mmol, 1.0 eq.) was suspended in nitrobenzene (8 mL). $AlCl_3$ (4.23 g, 31.72 mmol, 4.0 eq.) was added in three portions at room temperature. The reaction mixture was stirred at room temperature for 30 min. 3-Methylbutyryl chloride or 2-Methylbutyryl chloride (1.16 mL, 9.52 mmol, 1.2 eq.; 1.18mL, 9.52mmol, 1.2 eq.) was added, and the reaction mixture was heated at 65 °C for 34 h. The reaction mixture was then quenched by pouring into ice-water and the product was extracted with EtOAc (3 x 20 mL). The product was then extracted into 2 M NaOH solution (2 x 15 mL). The aqueous extracts were neutralized with conc. HCl to give a white precipitate. The product was extracted back into EtOAc (3 x 30 mL), then washed with water and brine, dried over Na_2SO_4, filtered and concentrated under reduced pressure. Purification by flash chromatography on silica gel gave **4a** (88% yield) and **4b** (84% yieid).

2.2 Synthesis of 5a and 5b

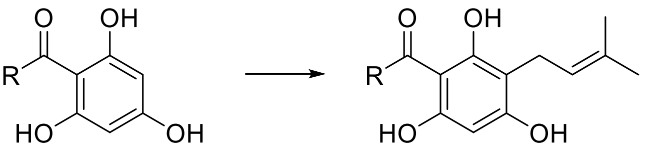

4a/4b **5a/5b**

To a solution of **4a**/**4b** (200 mg, 0.95 mmol, 1.0 eq.) and prenyl bromide (196 μL, 1.9 mmol, 2.0 eq.) in dry THF (3 mL) was added DBU (284 μL, 1.9 mmol, 2.0 eq.). The resultant suspension was heated at 45 °C for 24 h. The reaction mixture allowed to cool to room temperature, then acidified with 1 N HCl solution (1 mL) and extracted with EtOAc (3 x 10 mL). The combined extracts were washed with brine (3 x 10 mL), dried over Na_2SO_4, filtered and concentrated under reduced pressure. The residue was purified by flash chromatography on silica gel to give **5a** (124 mg, 47%) and **5b** (129 mg, 49%) as a yellow oil. Date for **5a**: R_f 0.6 (petrol/EtOAc, 1:1); ^{1}H NMR (400 MHz, CD_3OD) δ 5.89 (s, 1H), 5.21 – 5.11 (m, 1H), 3.18 (d, J = 7.1 Hz, 2H), 2.91 (d, J = 6.8 Hz, 2H), 2.21 (dt, J = 13.5, 6.8 Hz, 1H), 1.74 (s, 3H), 1.67 – 1.62 (m, 3H), 0.95 (d, J = 6.7 Hz, 6H). ^{13}C NMR (150 MHz, CD_3OD) δ 205.67, 163.65, 162.17, 159.96, 129.65, 123.17, 106.62, 103.06, 93.45, 52.40, 25.44, 24.59, 21.82, 20.78, 16.48; HRMS ($C_{16}H_{22}O_4$, ESI): calculated [M-H]$^-$ 277.1445, found 277.1446. Date for **5b**: R_f 0.6 (petrol/EtOAc, 1:1); ^{1}H NMR (400 MHz, CD_3OD) δ 5.88 (s, 1H), 5.15 (s, 1H), 3.87 (h, J = 6.7 Hz, 1H), 3.17 (d, J = 7.2 Hz, 2H), 1.86 – 1.76 (m, 1H), 1.76 – 1.72 (m, 3H), 1.67 – 1.62 (m, 3H), 1.36 (dt, J = 13.8, 7.1 Hz, 1H), 1.11 (d, J = 6.7 Hz, 3H), 0.90 (t, J = 7.4 Hz, 3H). ^{13}C NMR (150 MHz, CD_3OD) δ 210.10, 163.85, 162.01, 159.70, 129.62, 123.19, 106.70, 103.70, 93.54, 45.29, 26.81, 24.57, 20.81, 16.45, 15.87, 10.99; HRMS ($C_{16}H_{22}O_4$, ESI): calculated [M-H]$^-$ 277.1445 , found 277.1446.

2.3 Synthesis of 6a and 6b

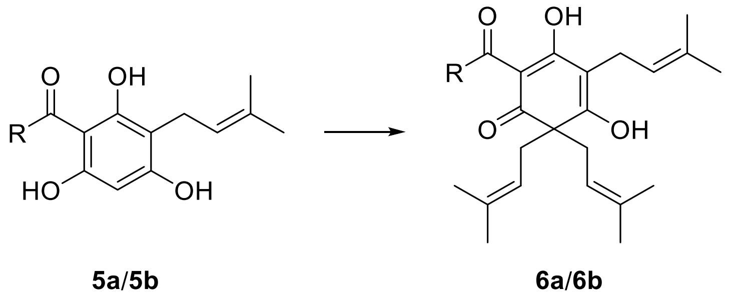

5a/5b **6a/6b**

To a solution of **5a**/**5b** (100 mg, 0.36 mmol, 1.0 eq.) in H_2O (2 mL) under a nitrogen atmosphere at 0 °C was added KOH (40 mg, 0.72 mmol, 2.0 eq.) in one portion, then prenyl bromide (70 μL, 0.72 mmol, 2.0 eq.) was added dropwise over 20 min. The reaction mixture was stirred at 0 °C for a further 2 h, during which time a thick orange precipitate was formed. The reaction mixture was then acidified with 1 N HCl solution (1 mL) and then extracted with EtOAc (3 x 10 mL). The combined extracts were washed with brine (3 x 10 mL), dried over Na_2SO_4, filtered and concentrated under reduced pressure. The residue was purified by flash chromatography on silica gel (petrol/EtOAc, 60:1→30:1) to give **6a** (90 mg, 61%) as a yellow oil and **6b** (88 mg, 59%) as a white oil. Date for **6a**: R_f 0.5 (petrol/EtOAc, 2:1); ^{1}H NMR (400 MHz, CD_3OD) δ 5.25 – 5.09

(m, 1H), 4.85 – 4.73 (m, 2H), 3.20 (d, *J* = 7.3 Hz, 2H), 2.92 (d, *J* = 7.0 Hz, 2H), 2.67 – 2.61 (m, 2H), 2.51 (dd, *J* = 13.8, 7.8 Hz, 2H), 2.19 – 2.09 (m, 1H), 1.79 (d, *J* = 5.0 Hz, 6H), 1.56 (s, 12H), 0.98 – 0.95 (m, 6H). ^{13}C NMR (150 MHz, CD_3OD) δ 203.97, 198.19, 191.00, 174.44, 135.67, 132.59, 123.22, 119.21, 112.39, 109.36, 58.79, 50.10, 38.77, 27.05, 26.03, 23.11, 21.61, 17.94; HRMS ($C_{26}H_{38}O_4$, ESI): calculated $[M-H]^-$ 413.2697, found 413.2703. Date for **6b**: R_f 0.5 (petrol/EtOAc, 2:1); ^{1}H NMR (400 MHz, CD_3OD) δ 5.01 (d, *J* = 7.2 Hz, 1H), 4.77 (d, *J* = 7.4 Hz, 2H), 3.89 (q, *J* = 7.0 Hz, 1H), 3.10 (d, *J* = 7.0 Hz, 2H), 2.66 – 2.51 (m, 4H), 1.73 (d, *J* = 5.3 Hz, 4H), 1.67 (s, 3H), 1.58 – 1.53 (m, 12H), 1.38 – 1.33 (m, 1H), 1.08 (d, *J* = 6.7 Hz, 3H), 0.90 (d, *J* = 7.4 Hz, 3H). ^{13}C NMR (150 MHz, CD_3OD) δ 208.30, 198.02, 191.21, 174.23, 135.63, 132.56, 123.24, 119.20, 112.35, 108.82, 58.73, 43.72, 39.05, 38.78, 27.70, 26.05, 21.65, 18.10, 16.99, 12.36; HRMS ($C_{26}H_{38}O_4$, ESI): calculated $[M-H]^-$ 413.2697, found 413.2707.

2.4 Synthesis of 1 and 2

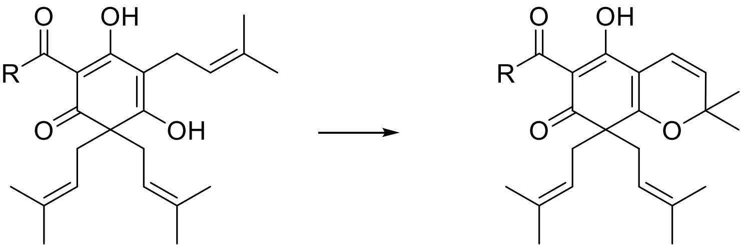

6a/6b

1 R = *i*Bu
2 R = *s*Bu

To a solution of **6a**/**6b** (50 mg, 0.12 mmol, 1.0 eq.) in dry THF (2 mL) at -78 °C under a nitrogen atmosphere was added TEMPO (38 mg, 0.24 mmol, 2.0 eq.) followed by $PhI(OAc)_2$ (45 mg, 0.14 mmol, 1.2 eq.). The reaction mixture was stirred at -78 °C for 5 min, then allowed to warm to room temperature over 30 min. The reaction mixture was quenched with H_2O (1 mL), then extracted with EtOAc (3 x 10 mL). The combined organics were dried over Na_2SO_4, filtered and concentrated under reduced pressure. The residue was then purified by flash chromatography on silica gel (petrol/EtOAc, 100:1→40:1) to give **1** (33 mg, 69%) and **2** (36 mg, 72%) as a brown oil. Date for **1**: R_f 0.6 (petrol/EtOAc, 10:1); ^{1}H NMR (400 MHz, CD_3OD) δ 6.44 (d, *J* = 10.1 Hz, 1H), 5.45 (dd, *J* = 22.8, 10.1 Hz, 1H), 4.80 – 4.68 (m, 2H), 2.91 (d, *J* = 7.0 Hz, 2H), 2.65 (dd, *J* = 13.8, 7.8 Hz, 2H), 2.51 (dd, *J* = 13.9, 7.3 Hz, 2H), 2.07 (dq, *J* = 13.4, 6.4 Hz, 1H), 1.57 (d, *J* = 8.8 Hz, 12H), 1.44 (d, *J* = 11.8 Hz, 6H), 0.95 (d, *J* = 6.8 Hz, 6H). ^{13}C NMR (150 MHz, CD_3OD) δ 203.52, 197.30, 187.49, 173.38, 136.00, 125.06, 119.16, 118.66, 114.98, 109.27, 107.61, 82.85, 58.24, 49.39, 38.66, 37.35, 29.08, 27.27, 25.98, 22.96, 18.31; HRMS ($C_{26}H_{36}O_4$, ESI): calculated $[M-H]^-$ 411.2541 , found 411.2549. Date for **2**: R_f 0.6 (petrol/EtOAc, 10:1); ^{1}H NMR (400 MHz, CD_3OD) δ 6.44 (d, *J* = 10.1 Hz, 1H), 5.48 (d, *J* = 10.2 Hz, 1H), 4.82 – 4.77 (m, 2H), 3.95 – 3.79 (m, 1H), 2.67 (dt, *J* = 14.3, 7.4 Hz, 2H), 2.51 (dd, *J* = 13.9, 7.4 Hz, 2H), 1.76 – 1.70 (m, 1H), 1.57 (d, *J* = 9.0 Hz, 12H), 1.46 (s, 6H), 1.29 (s, 1H), 1.09 (d, *J* = 6.9 Hz, 3H), 0.90 (d, *J* = 7.5 Hz, 3H). ^{13}C NMR (150 MHz, CD_3OD) δ 208.09, 197.18, 187.54, 173.34, 136.00,

125.07, 119.24, 114.99, 108.70, 107.49, 82.95, 43.42, 58.43, 38.84, 38.51, 29.07, 27.71, 26.01, 18.29, 16.94, 12.33; HRMS ($C_{26}H_{36}O_4$, ESI): calculated $[M+H]^+$ 413.2686, found 413.2690.

2.5 Synthesis of 7a and 7b

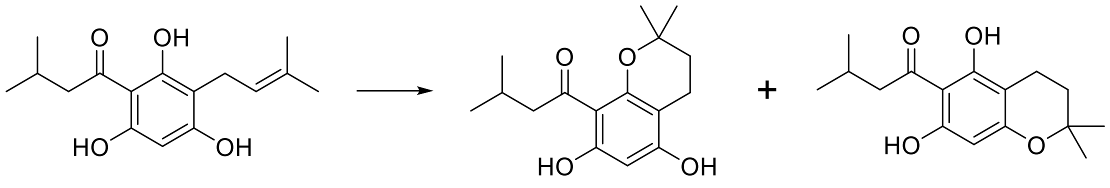

5a **7a** **7b**

To a solution of **5a** (100 mg, 0.36 mmol, 1.0 eq.) in toluene (3 mL) at room temperature was added PSTA (124 mg, 0.72 mmol, 2.0 eq.).The reaction mixture was stirred at room temperature for 2h. The reaction mixture was quenched with H_2O (1 mL), then extracted with EtOAc (3 x 10 mL). The combined organics were dried over Na_2SO_4, filtered and concentrated under reduced pressure. The residue was then purified by flash chromatography on silica gel (petrol/EtOAc, 40:1→15:1) to give **7a** (49 mg 49%) as a white oil and on silica gel (petrol/EtOAc, 40:1→20:1) to give **7b** (33 mg 33%) as a yellow oil. Date for **7a**: R_f 0.4 (petrol/EtOAc, 4:1) ^{1}H NMR (400 MHz, $CDCl_3$) δ 13.92 (s, 1H), 5.93 (s, 1H), 2.90 (d, J = 7.0 Hz, 2H), 2.59 (t, J = 6.8 Hz, 2H), 2.21 (dq, J = 13.4, 6.7 Hz, 1H), 1.80 (t, J = 6.8 Hz, 2H), 1.40 (s, 6H), 0.97 (d, J = 6.6 Hz, 6H). ^{13}C NMR (150 MHz, $CDCl_3$) δ 206.01, 165.07, 159.94, 157.14, 106.32, 99.36, 95.34, 76.04, 53.40, 31.51, 26.77, 25.39, 22.77, 16.35; HRMS ($C_{16}H_{22}O_4$, ESI): calculated $[M-H]^-$ 277.1445, found 277.1442. Date for **7b**: R_f 0.5 (petrol/EtOAc, 4:1) ^{1}H NMR (400 MHz, $CDCl_3$) δ 13.57 (s, 1H), 6.43 (s, 1H), 5.72 (s, 1H), 2.93 (d, J = 6.8 Hz, 2H), 2.58 (t, J = 6.8 Hz, 2H), 2.26 (dp, J = 13.4, 6.7 Hz, 1H), 1.78 (t, J = 6.8 Hz, 2H), 1.32 (s, 6H), 0.97 (d, J = 6.7 Hz, 6H). ^{13}C NMR (150 MHz, $CDCl_3$) δ 205.87, 164.00, 160.40, 158.01, 104.46, 101.74, 95.72, 76.13, 52.91, 32.31, 26.89, 25.62, 23.04, 16.30; HRMS ($C_{16}H_{22}O_4$, ESI): calculated $[M-H]^-$ 277.1445, found 277.1452.

2.6 Synthesis of 8

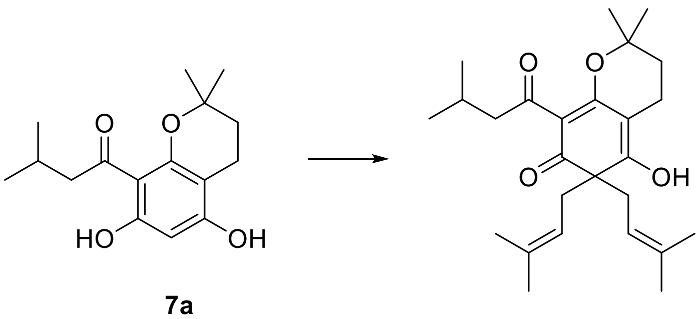

7a **8**

To a solution of **7a** (40 mg, 0.14 mmol, 1.0 eq.) in H_2O (1.5 mL) under a nitrogen atmosphere at 0 °C was added KOH (16 mg, 0.28 mmol, 2.0 eq.) in one portion, then prenyl bromide (27 μL, 0.28 mmol, 2.0 eq.) was added dropwise over 20 min. The reaction mixture was stirred at 0 °C for a further 2 h, during which time a thick orange

precipitate was formed. The reaction mixture was then acidified with 1 N HCl solution (1 mL) and then extracted with EtOAc (3 x 10 mL). The combined extracts were washed with brine (3 x 10 mL), dried over Na_2SO_4, filtered and concentrated under reduced pressure. The residue was purified by flash chromatography on silica gel (petrol/EtOAc, 60:1→35:1) to give **8** (34 mg, 61%) as a pale yellow oil. Date for **8**: R_f 0.6 (petrol/EtOAc, 4:1); ^{1}H NMR (400 MHz, CD_3OD) δ 4.78 – 4.70 (m, 2H), 2.78 (d, J = 7.3 Hz, 2H), 2.69 – 2.55 (m, 4H), 2.34 (t, J = 6.8 Hz, 2H), 2.10 (dt, J = 13.6, 6.8 Hz, 1H), 1.78 (t, J = 6.8 Hz, 2H), 1.60 – 1.46 (m, 12H), 1.40 (s, 6H), 0.96 (d, J = 6.7 Hz, 6H). ^{13}C NMR (150 MHz, CD_3OD) δ 201.38, 198.09, 196.61, 166.61, 136.01, 119.17, 108.78, 107.39, 80.25, 61.01, 49.43, 39.67, 32.52, 28.58, 26.66, 26.03, 22.87, 17.98, 17.32; HRMS ($C_{26}H_{38}O_4$, ESI): calculated [M-H]$^-$ 413.2770, found 413.2697.

2.7 Synthesis of 9

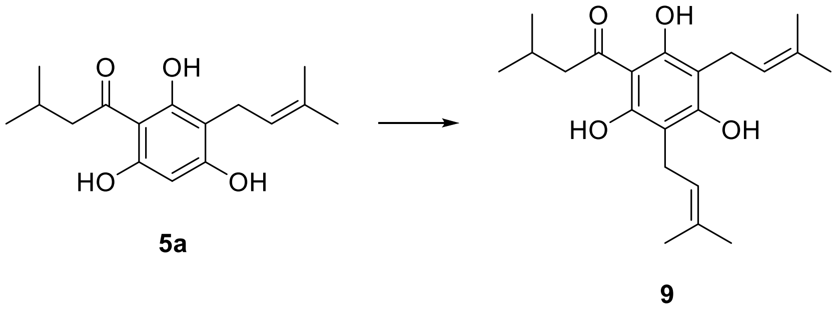

Stir a solution of **5a** (100 mg, 0.36 mmol, 1.0 eq.) in H_2O (2 mL) under a nitrogen atmosphere at 0 °C was added KOH (40 mg, 0.72 mmol, 2.0 eq.) in one portion, then prenyl bromide (70 µL, 0.72 mmol, 2 eq.) was added dropwise over 20 min. The resultant suspension was stirred at 0 °C for a further 15 min. The reaction mixture was then acidified with 1 N HCl solution (2 mL) and then extracted with EtOAc (3 x 10 mL). The combined extracts were washed with brine (3 x 15 mL), dried over Na_2SO_4, filtered and concentrated under reduced pressure. The residue was purified by flash chromatography on silica gel (petrol/EtOAc, 60:1→40:1) to give **9** (67 mg, 53%) as a brown oil. Date for **9**: R_f 0.6 (petrol/EtOAc, 5:1); ^{1}H NMR (400 MHz, CD_3OD) δ 5.20 – 5.04 (m, 2H), 3.28 (d, J = 6.6 Hz, 4H), 2.96 (d, J = 6.8 Hz, 2H), 2.22 (dt, J = 13.4, 6.7 Hz, 1H), 1.76 (s, 6H), 1.67 (d, J = 1.6 Hz, 6H), 0.95 (d, J = 6.7 Hz, 6H); ^{13}C NMR (150 MHz, CD_3OD) δ 207.87, 161.02, 159.94, 132.68, 123.96, 108.66, 107.27, 54.17, 26.86, 25.95, 23.21, 22.66, 17.98; HRMS ($C_{21}H_{30}O_4$, ESI): calculated [M-H]$^-$ 345.2071, found 345.2079.

2.8 Synthesis of 10

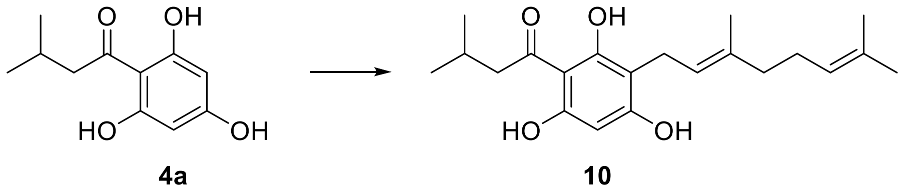

To a mixture solution of **4a** (100 mg, 0.48 mmol, 1.0 eq.) and geranyl bromide (190 µL,

0.96 mmol, 2.0 eq.) in DMF (2 mL) was added DIPEA (171 μL 0.96 mmol, 2.0 eq.). The resultant suspension was heated at 80 °C for 3 h, then gradually warmed to room temperature. The reaction mixture was quenched with H_2O (1 mL) and extracted with EtOAc (3 x 10 mL). The combined organic extracts were dried over Na_2SO_4, filtered and concentrated under reduced pressure. The residue was then purified by flash chromatography on silica gel (petrol/EtOAc, 20:1→5:1) to give **10** (78 mg 47%) as a yellow oil. Date for **10** : R_f 0.4 (petrol/EtOAc, 2:1); ^{1}H NMR (400 MHz, CD_3OD) δ 5.88 (s, 1H), 5.17 (dt, J = 6.1, 4.3 Hz, 1H), 5.05 (dt, J = 7.3, 1.5 Hz, 1H), 3.18 (d, J = 7.1 Hz, 2H), 2.91 (d, J = 6.8 Hz, 2H), 2.21 (dt, J = 13.1, 6.6 Hz, 1H), 2.04 (q, J = 7.4 Hz, 2H), 1.93 (t, J = 7.5 Hz, 2H), 1.75 – 1.72 (m, 3H), 1.63 – 1.56 (m, 3H), 1.55 (s, 3H), 0.95 (d, J = 6.7 Hz, 6H). ^{13}C NMR (150 MHz, CD_3OD) δ 207.03, 165.37, 163.87, 161.63, 134.93, 130.94, 125.80, 124.94, 108.35, 105.75, 95.12, 53.80, 40.93, 28.01, 27.13, 26.13, 23.50, 22.09, 17.71, 16.48; HRMS ($C_{21}H_{30}O_4$, ESI): calculated $[M+H]^+$ 347.2217, found 347.2217.

2.9 Synthesis of 11

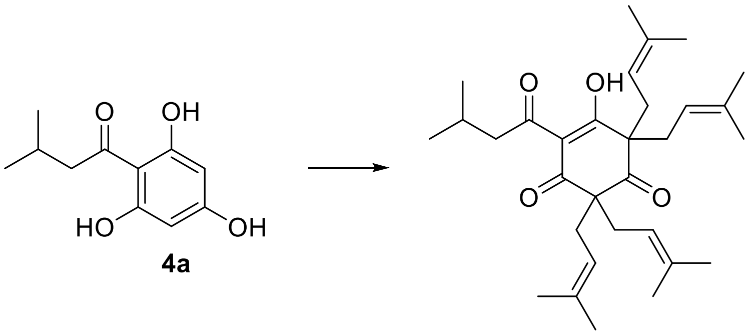

11

Stir a solution of **4a** (40 mg, 0.19 mmol, 1.0 eq.) in $NH_3{\cdot}H_2O$ (1.5 mL). The reaction mixture was stirred at room temperature for 15 min, then prenyl bromide (45 μL, 0.38 mmol, 2.0 eq.) was added dropwise. The reaction mixture was stirred for 4 h at rt, then acidified with 1 N HCl solution (1.5 mL) and extracted with EtOAc (3 x 15 mL). The combined extracts were washed with brine (3 x 10 mL), dried over Na_2SO_4, filtered and concentrated under reduced pressure. The residue was purified by flash chromatography on silica gel (petrol/EtOAc, 100:1→60:1) to give **11** (13 mg, 17%) as a white oil. Data for **11**: R_f 0.4 (petrol/EtOAc, 20:1); ^{1}H NMR (400 MHz, $CDCl_3$) δ 4.90 (s, 4H), 2.81 (s, 2H), 2.65 (dd, J = 13.8, 8.0 Hz, 2H), 2.53 (dt, J = 14.9, 7.9 Hz, 4H), 2.30 (dd, J = 14.6, 6.1 Hz, 2H), 2.23 – 2.14 (m, 1H), 1.63 – 1.54 (m, 24H), 0.99 (d, J = 6.7 Hz, 6H); ^{13}C NMR (150 MHz, $CDCl_3$) δ 207.55, 203.65, 197.41, 194.73, 136.16, 134.60, 118.89, 118.26, 113.57, 65.53, 60.94, 47.65, 36.82, 33.85, 25.96, 25.85, 25.77, 22.69, 17.88, 17.84; HRMS ($C_{31}H_{46}O_4$, ESI): calculated $[M+H]^+$ 483.3469, found 483.3472.

2.10 Synthesis of 12

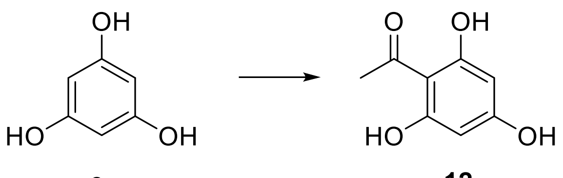

3 **12**

Phloroglucinol (1.0 g, 7.93 mmol, 1.0 eq.) was suspended in nitrobenzene (8 mL). $AlCl_3$ (4.23 g, 31.7 mmol, 4.0 eq.) was added in three portions at room temperature. The reaction mixture was stirred at room temperature for 30 min. Acyl chloride (0.914 mL, 8.72 mmol, 1.2 eq.) was added, and the reaction mixture was heated at 65 °C for 34 h. The reaction mixture was then quenched by pouring into ice-water and the product was extracted with EtOAc (3 x 20 mL). The product was then extracted into 2 M NaOH solution (2 x 15 mL). The aqueous extracts were neutralized with conc. HCl to give a white precipitate. The product was extracted back into EtOAc (3 x 30 mL), then washed with water and brine, dried over Na_2SO_4, filtered and concentrated under reduced pressure. Purification by flash chromatography on silica gel gave **12** (81% yield).

2.11 Synthesis of 13

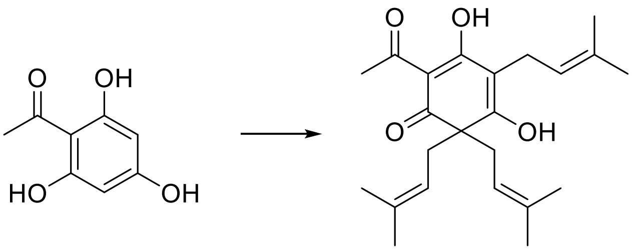

12 **13**

To a solution of **12** (400 mg, 2.9 mmol, 1.0 eq.) in dry THF (4 mL) at room temperature was added DBU (834 μL, 8.7 mmol, 2.0 eq.). The reaction mixture was stirred at room temperature for 5 min, then prenyl bromide (1.3mL, 8.7 mmol, 2.0 eq.) was added dropwise. The reaction mixture was stirred for 24 h at 45°C, then acidified with 1 N HCl solution (2 mL) and extracted with EtOAc (3 x 20 mL). The combined extracts were washed with brine (3 x 15 mL), dried over Na_2SO_4, filtered and concentrated under reduced pressure. The residue was purified by flash chromatography on silica gel (petrol/EtOAC, 80:1→40:1) to give **13** (230 mg, 39%) as a white crystal. Date for **13**: R_f 0.4 (petrol/EtOAc, 10:1); ^{1}H NMR (400 MHz, CD_3OD) δ 5.06 (t, J = 7.1 Hz, 1H), 4.80 (t, J = 7.5 Hz, 2H), 3.02 (d, J = 6.9 Hz, 2H), 2.52 (d, J = 7.5 Hz, 4H), 2.45 (s, 3H), 1.71 (s, 3H), 1.64 (s, 3H), 1.54 (d, J = 6.4 Hz, 12H); ^{13}C NMR (150 MHz, CD_3OD) δ 204.77, 198.89, 186.47, 174.46, 134.36, 132.10, 123.76, 119.36, 110.25, 108.11, 55.47, 38.52, 28.96, 25.38, 21.23, 17.40; HRMS ($C_{23}H_{32}O_4$, ESI): calculated $[M-H]^-$ 371.2228, found 371.2234.

2.12 Synthesis of 14

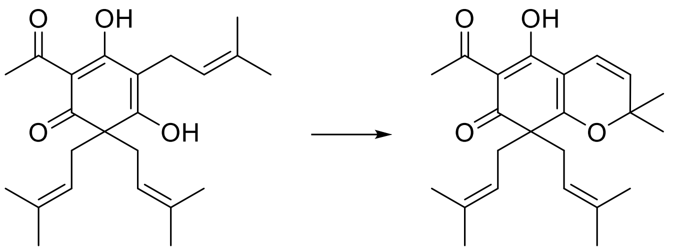

13 **14**

To a solution of **13** (200 mg, 0.54mmol, 1.0 eq.) in dry THF (4 mL) at -78 °C under a nitrogen atmosphere was added TEMPO (169 mg, 1.08 mmol, 2.0 eq.) followed by $PhI(OAc)_2$ (209 mg, 0.65 mmol, 1.2 eq.). The reaction mixture was stirred at -78 °C for 5 min, then allowed to warm to room temperature over 30 min. The reaction mixture was quenched with H_2O (2 mL), then extracted with EtOAc (3 x 30 mL). The combined organics were dried over Na_2SO_4, filtered and concentrated under reduced pressure. The residue was then purified by flash chromatography on silica gel (petrol/EtOAc, 80:1→30:1) to give **14** (110 mg, 64%) as a yellow oil. Date for **14**: R_f 0.4 (petrol/EtOAc, 10:1); ^{1}H NMR (400 MHz, $CDCl_3$) δ 6.48 (dd, J = 29.2, 10.0 Hz, 1H), 5.32 (dd, J = 14.7, 10.0 Hz, 1H), 4.77 (t, J = 7.1 Hz, 2H), 2.70 (d, J = 8.3 Hz, 2H), 2.58 (s, 3H), 2.48 (dd, J = 13.9, 7.6 Hz, 2H), 1.62 (d, J = 1.0 Hz, 12H), 1.41 (d, J = 13.9 Hz, 6H). ^{13}C NMR (150 MHz, $CDCl_3$) δ 199.48, 195.47, 185.85, 172.21, 134.60, 123.10, 117.90, 114.31, 108.20, 105.70, 81.03, 56.80, 37.34, 28.50, 25.50, 17.91; HRMS ($C_{23}H_{30}O_4$, ESI): calculated $[M\text{-}H]^-$ 369.2071, found 369.2075.

3. Spectral date scans of all compounds

Fig. S1. ^{1}H NMR Spectrum of Compound **4a** (400 MHz, CD_3OD)

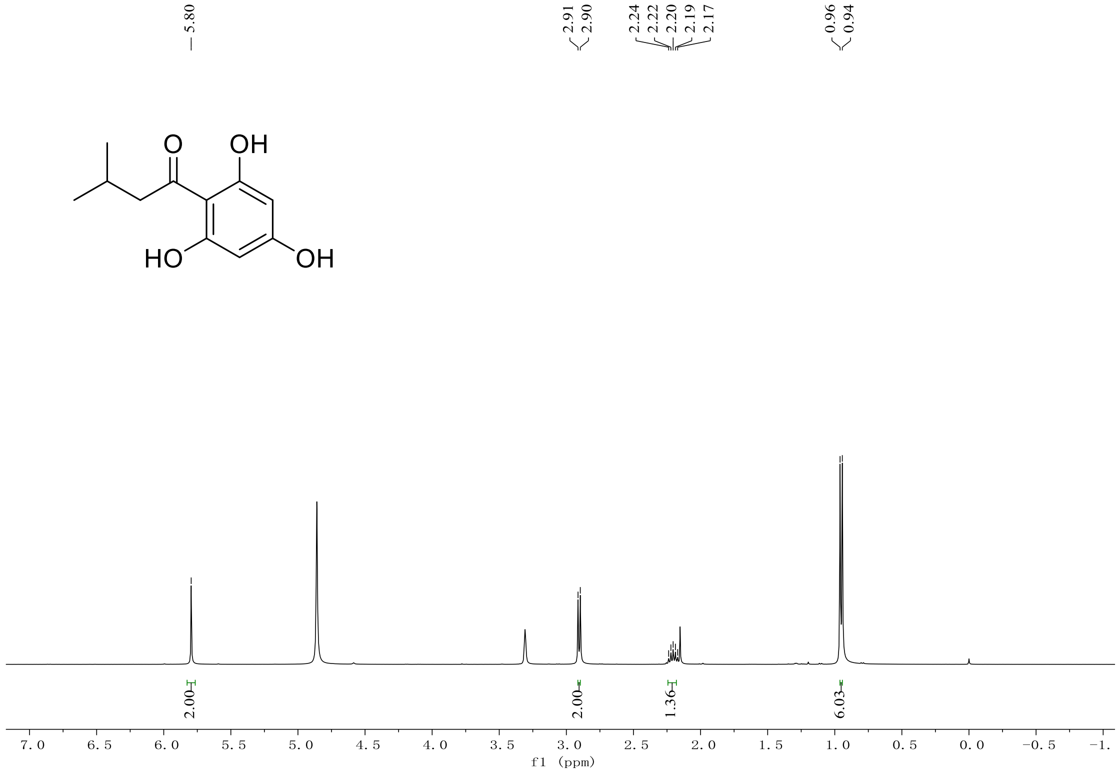

Fig. S2. ^{13}C NMR Spectrum of Compound **4a** (150 MHz, CD_3OD)

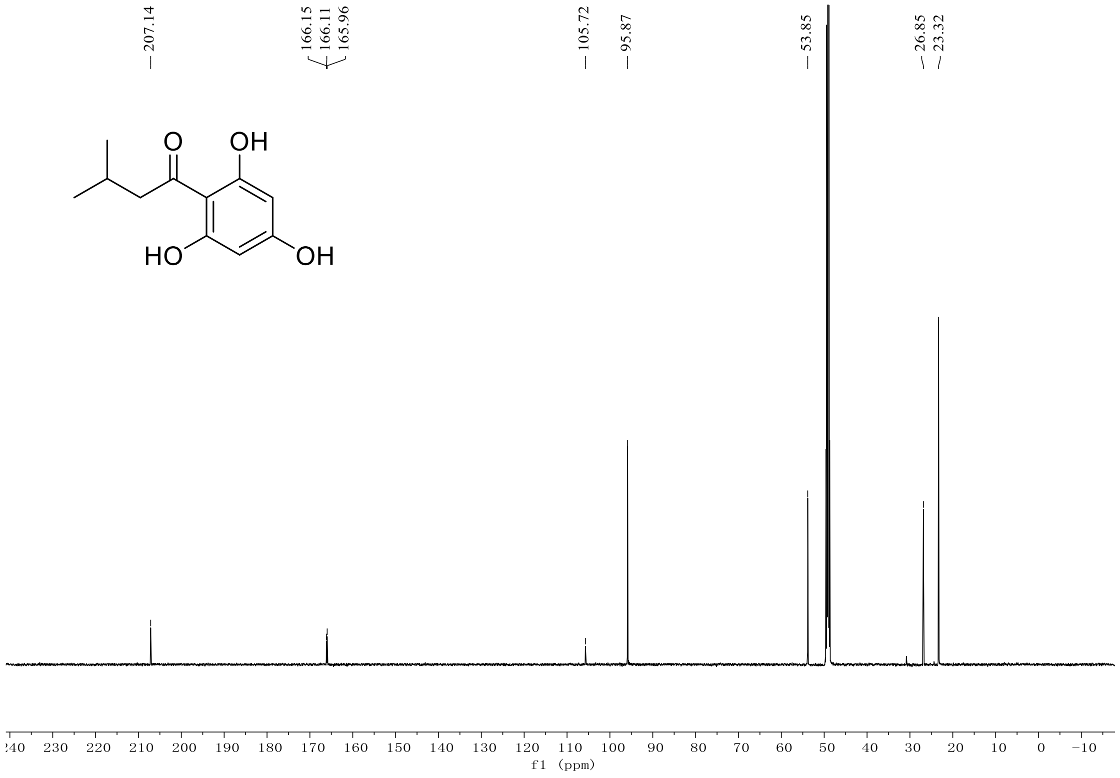

Fig. S3. ¹H NMR Spectrum of Compound **4b** (400 MHz, CD_3OD)

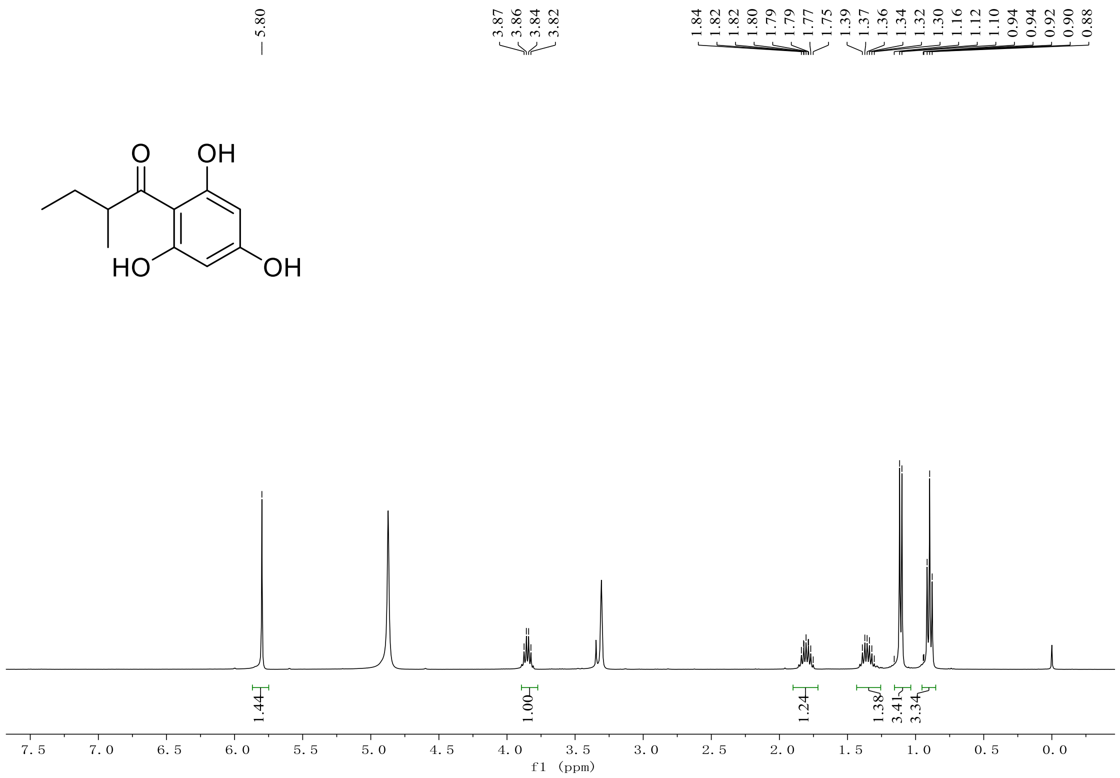

Fig. S4. ¹³C NMR Spectrum of Compound **4b** (150 MHz, CD_3OD)

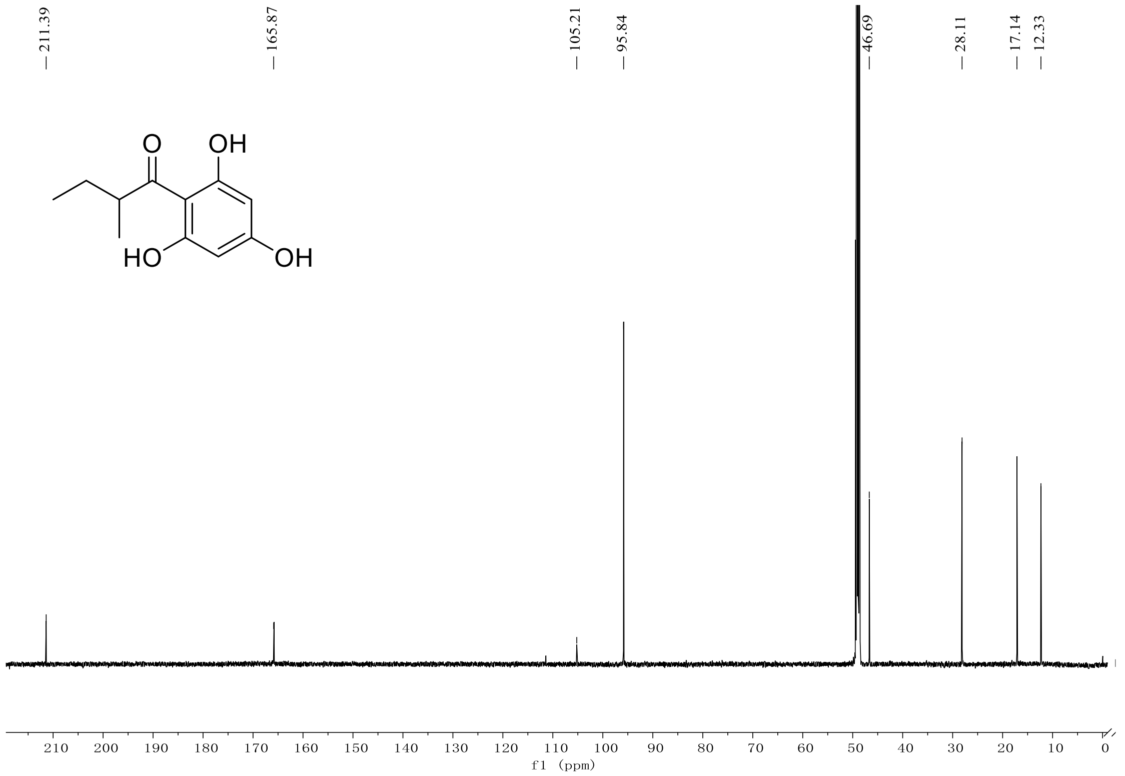

Fig. S5. ^{1}H NMR Spectrum of Compound **5a** (400 MHz, CD_3OD)

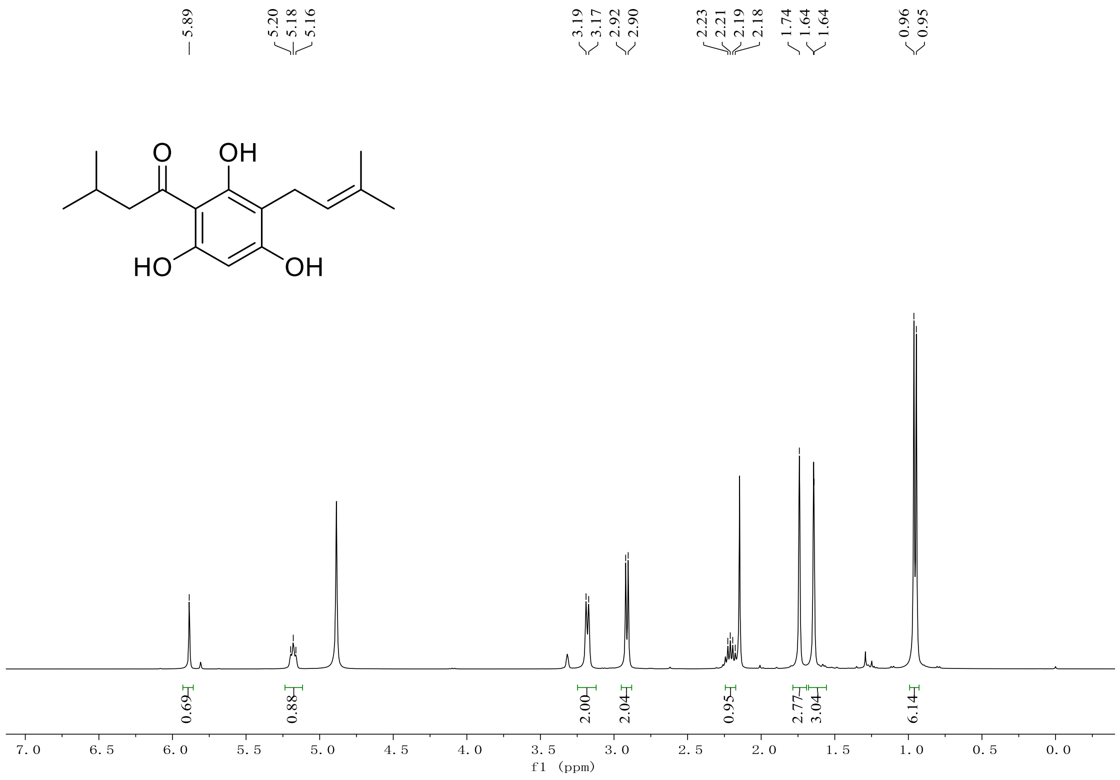

Fig. S6. ^{13}C NMR Spectrum of Compound **5a** (150 MHz, CD_3OD)

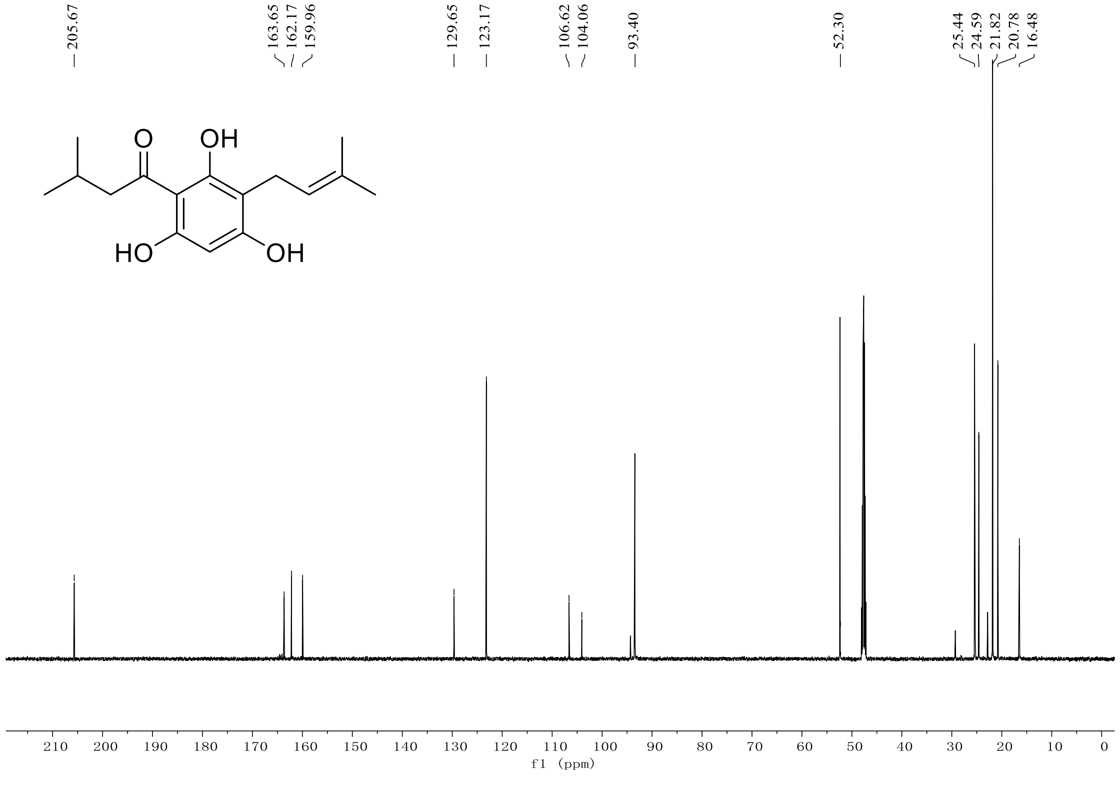

Fig. S7. ^{1}H NMR Spectrum of Compound **5b** (400 MHz, CD_3OD)

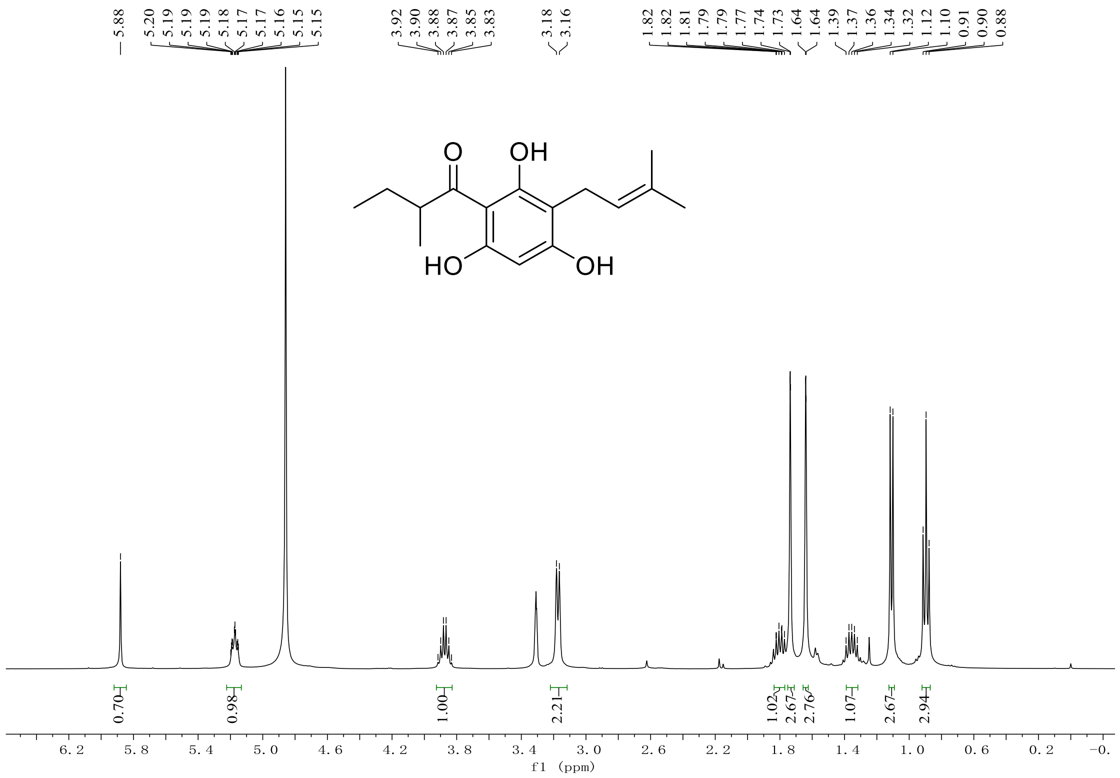

Fig. S8. ^{13}C NMR Spectrum of Compound **5b** (150 MHz, CD_3OD)

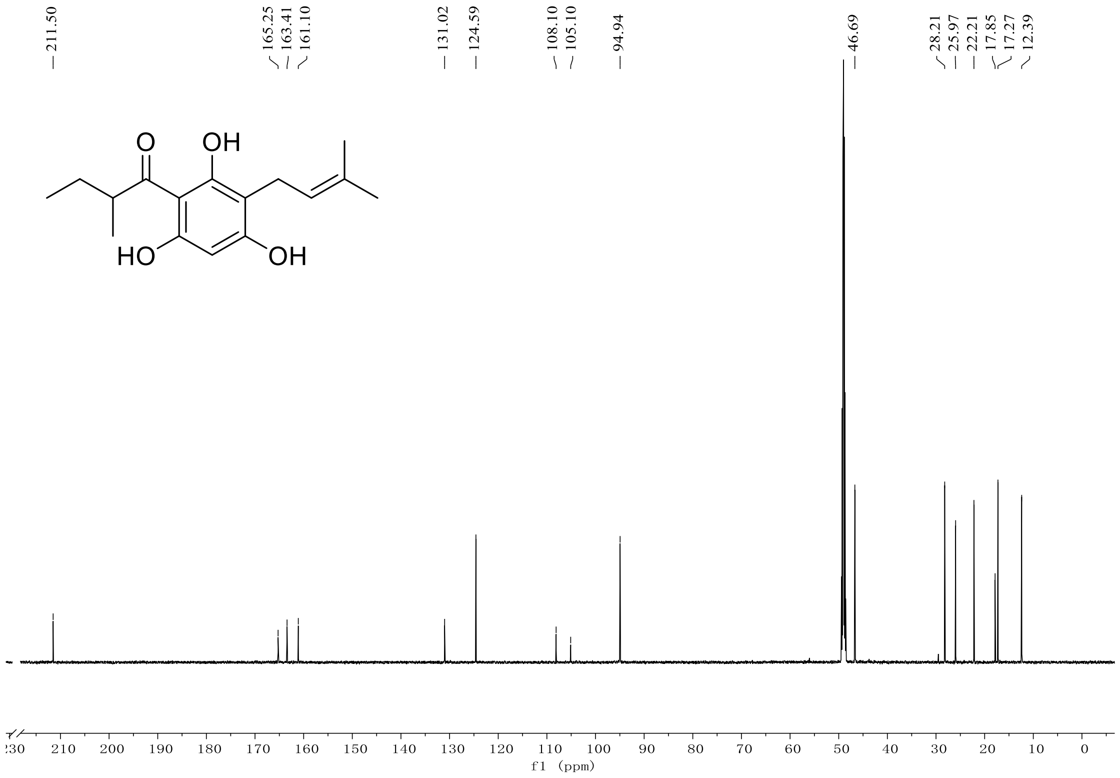

Fig. S9. ^{1}H NMR Spectrum of Compound **6a** (400 MHz, CD_3OD)

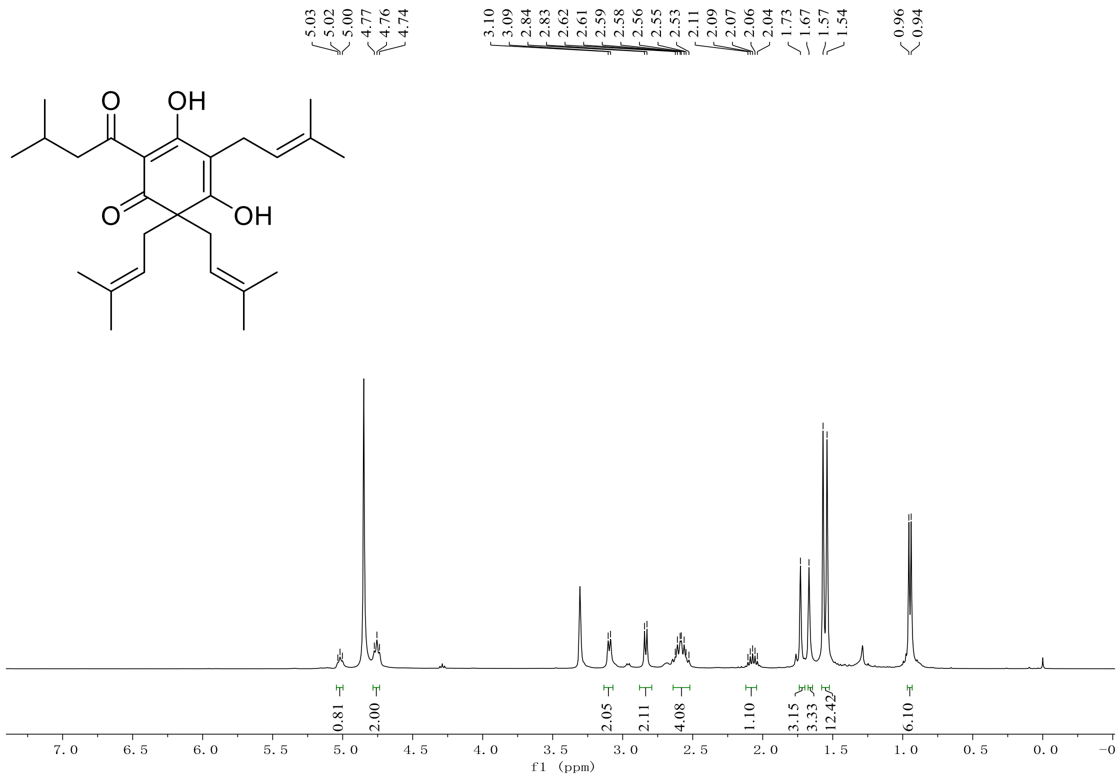

Fig. S10. ^{13}C NMR Spectrum of Compound **6a** (150 MHz, CD_3OD)

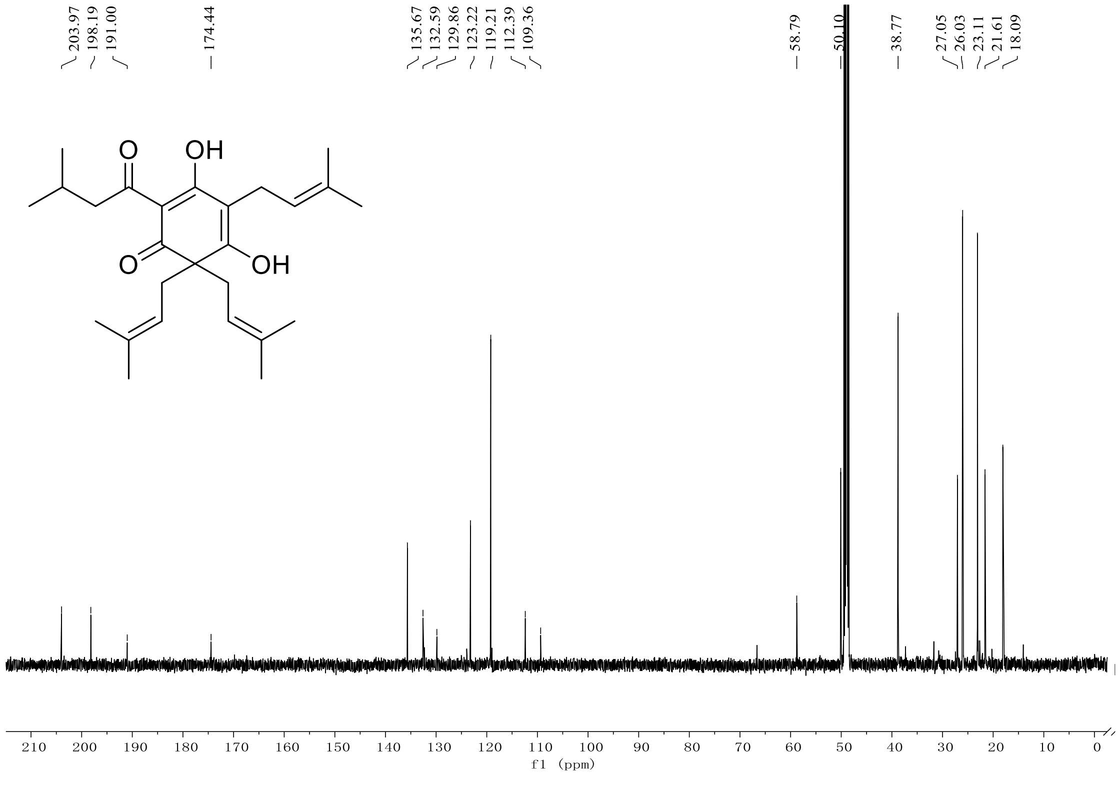

Fig. S11. ^{1}H NMR Spectrum of Compound **6b** (400 MHz, CD_3OD)

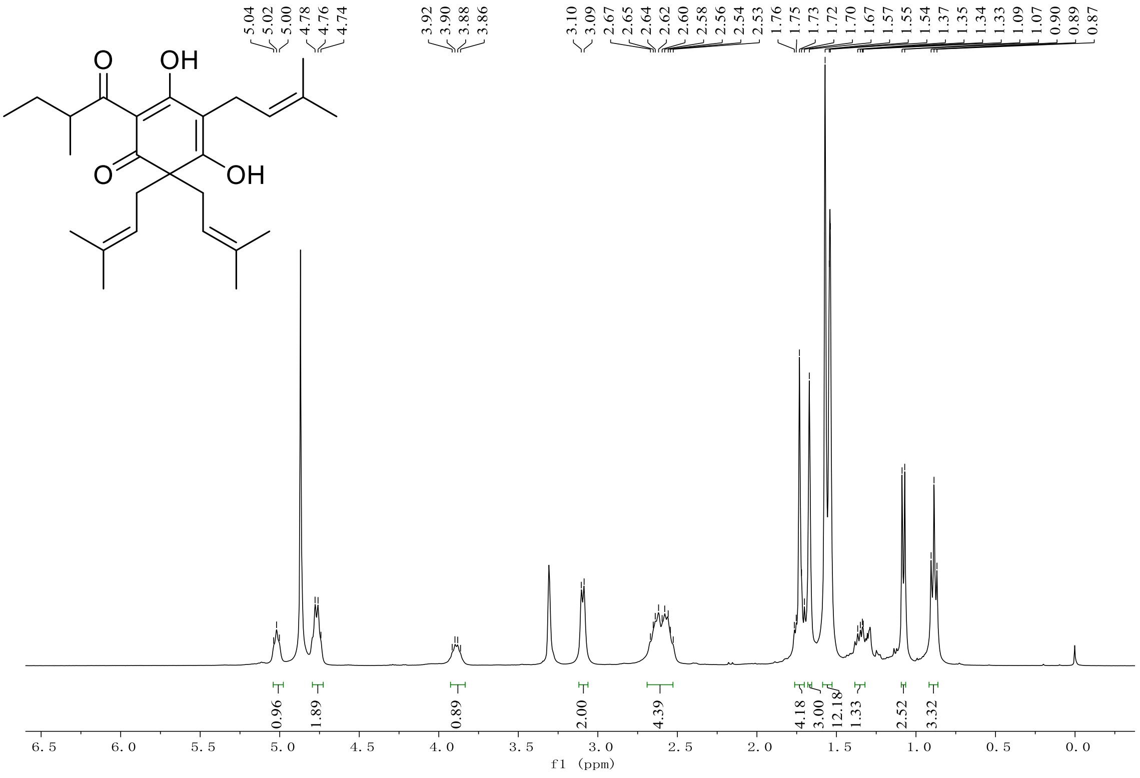

Fig. S12. ^{13}C NMR Spectrum of Compound **6b** (150 MHz, CD_3OD)

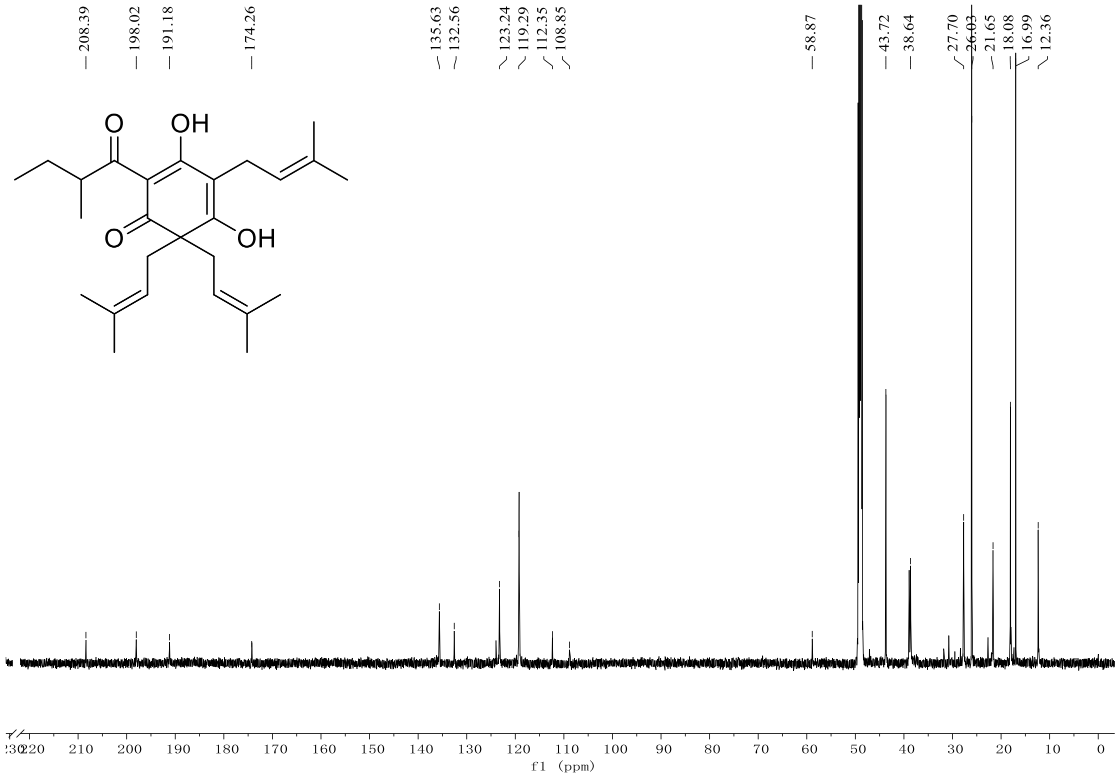

Fig. S13. ^{1}H NMR Spectrum of Compound **1** (400 MHz, CD_3OD)

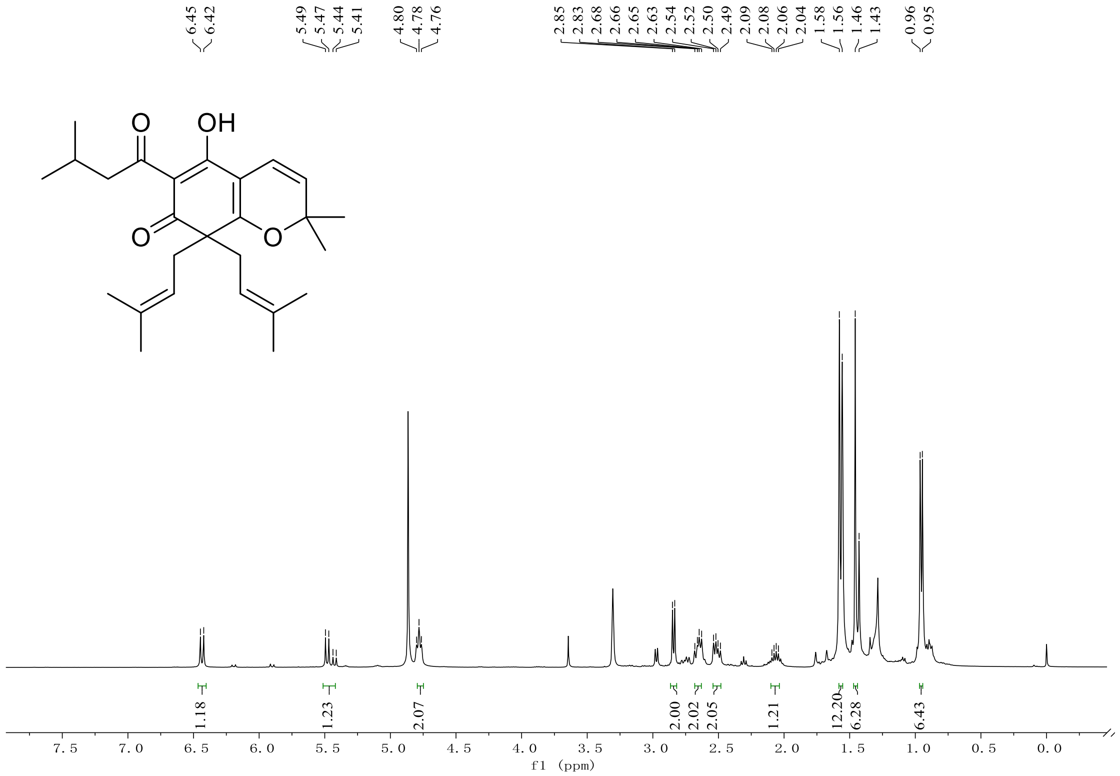

Fig. S14. ^{13}C NMR Spectrum of Compound **1** (150 MHz, CD_3OD)

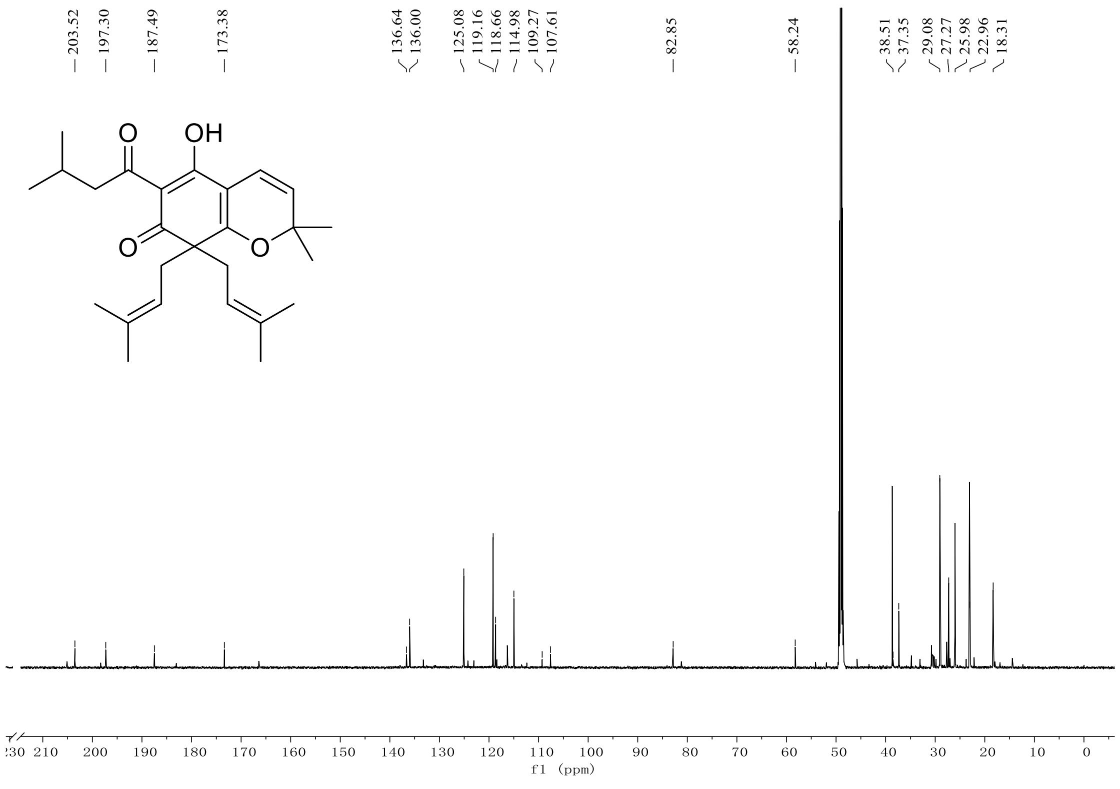

Fig. S15. ^{1}H NMR Spectrum of Compound **1** (400 MHz, $CDCl_3$)

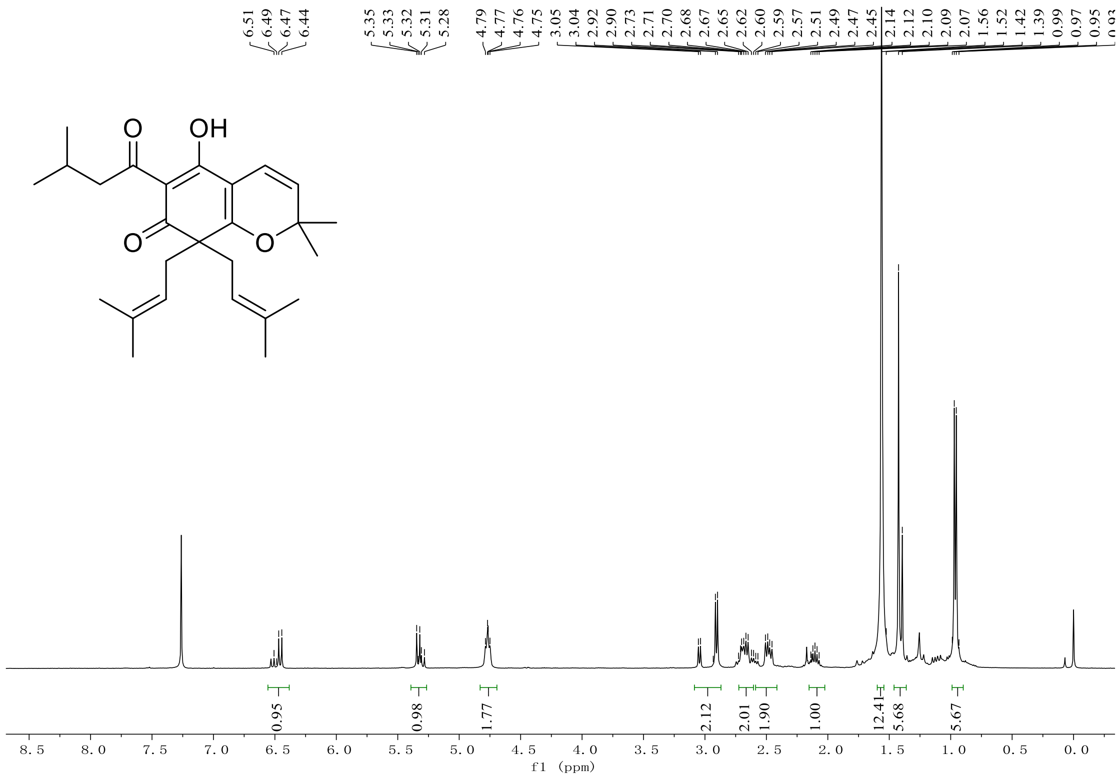

Fig. S16. ^{13}C NMR Spectrum of Compound **1** (150 MHz, $CDCl_3$)

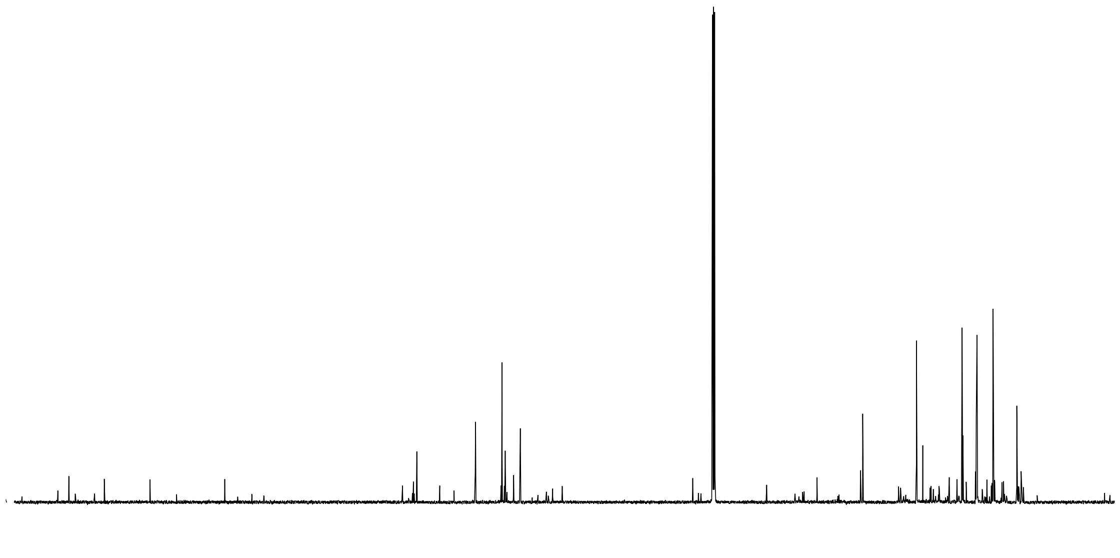

Fig. S17. Dept135 Spectrum of Compound **1** (150 MHz, CD_3OD)

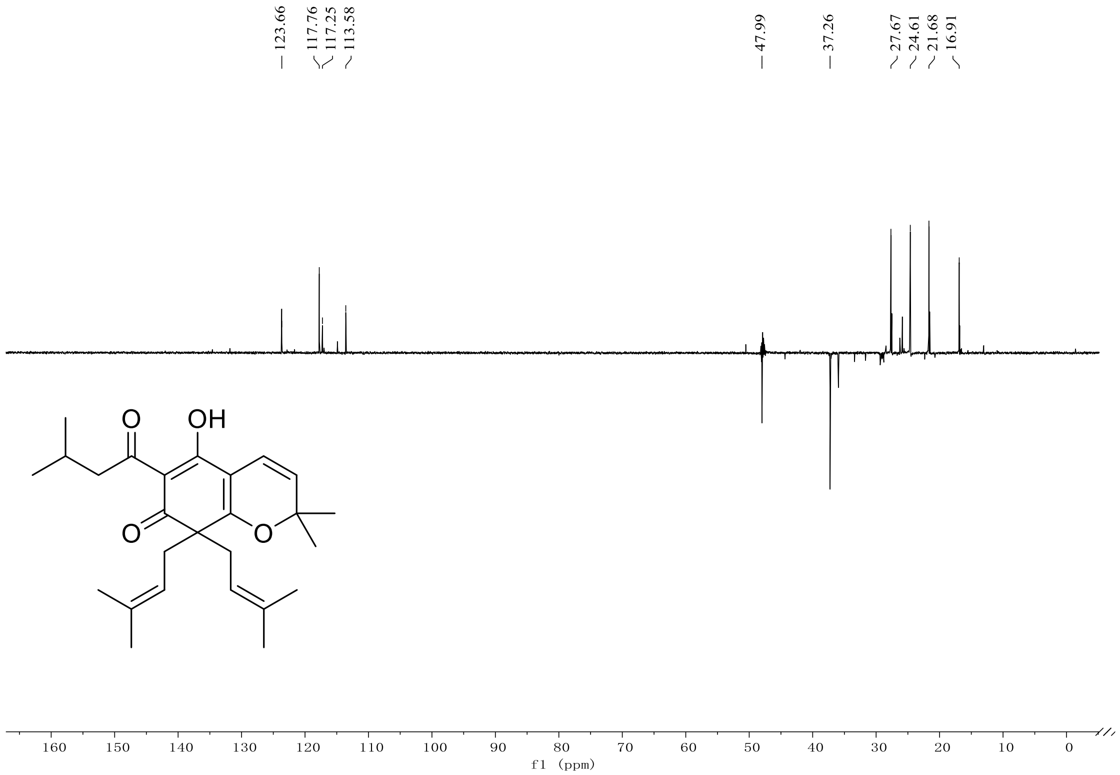

Fig. S18. Cosy Spectrum of Compound **1** (150 MHz, CD_3OD)

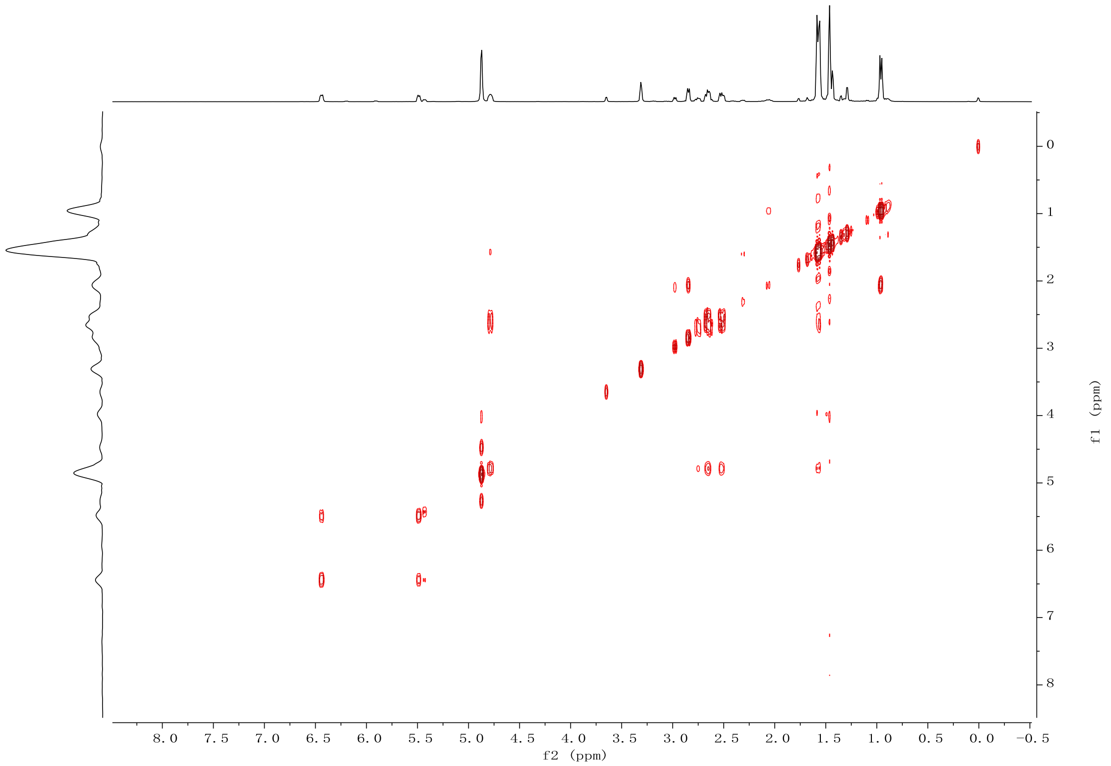

Fig. S19. HMBC Spectrum of Compound **1** (150 MHz, CD_3OD)

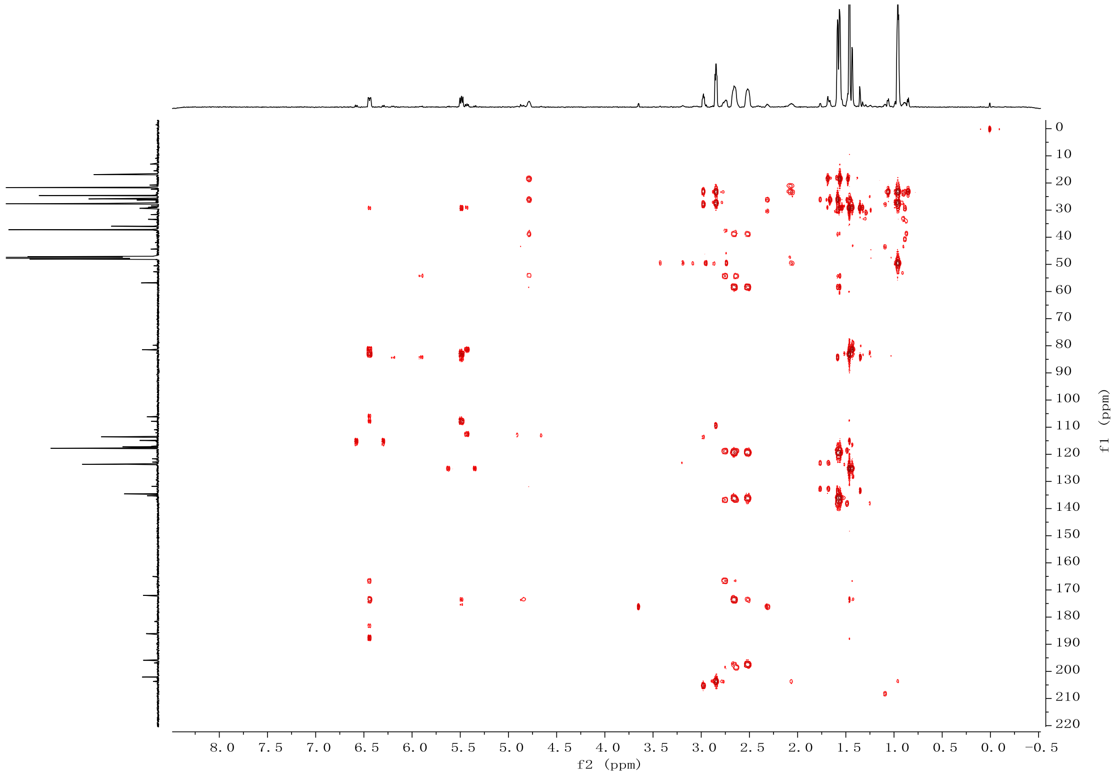

Fig. S20. HSQC Spectrum of Compound **1** (150 MHz, CD_3OD)

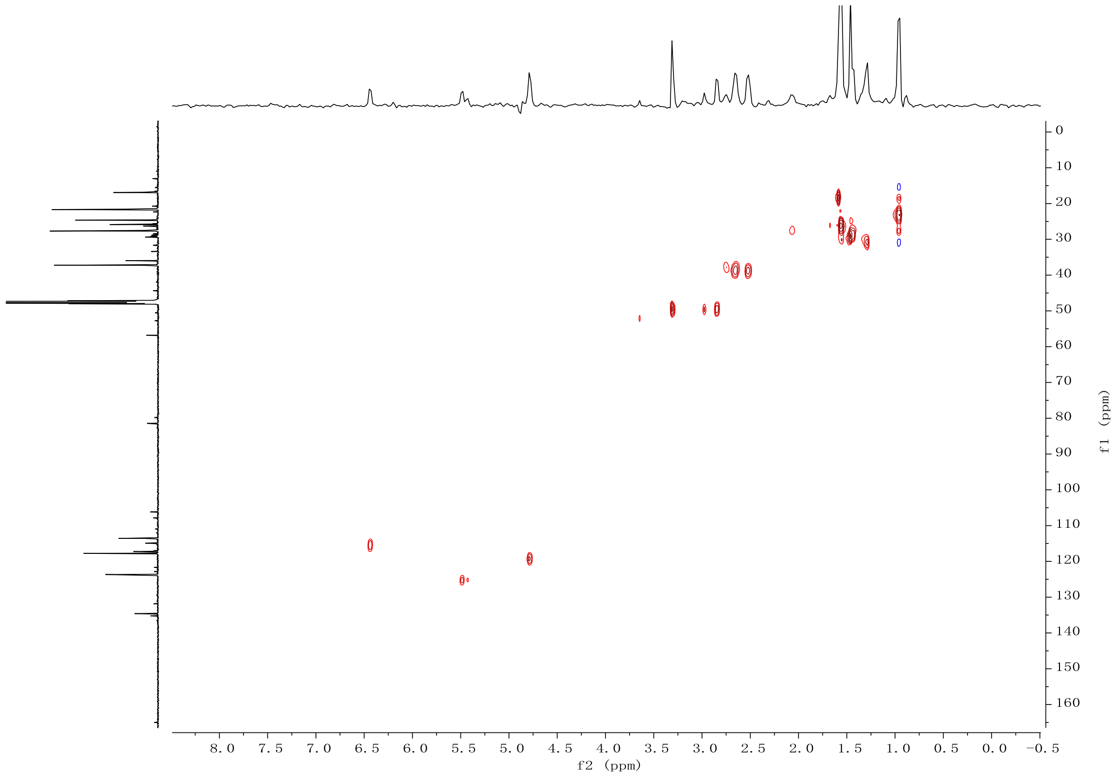

Fig. S21. Noesy Spectrum of Compound **1** (150 MHz, CD_3OD)

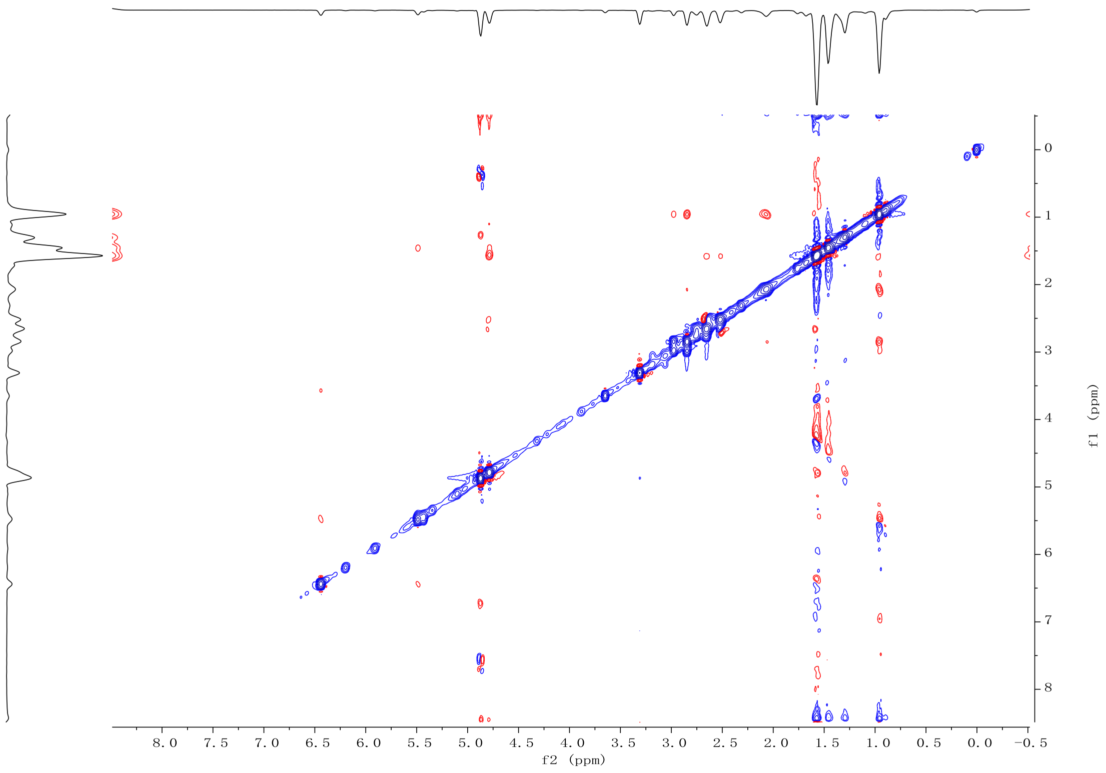

Fig. S22. ^{1}H NMR Spectrum of Compound **2** (400 MHz, CD_3OD)

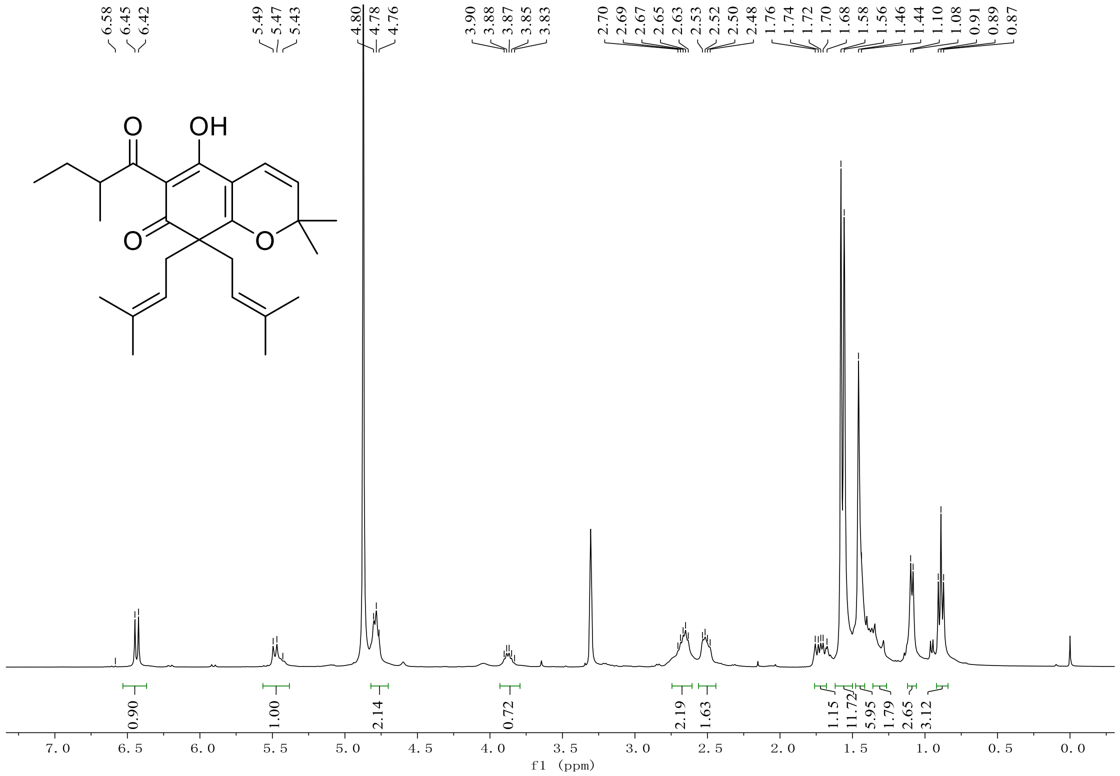

Fig. S23. ^{13}C NMR Spectrum of Compound **2** (150 MHz, CD_3OD)

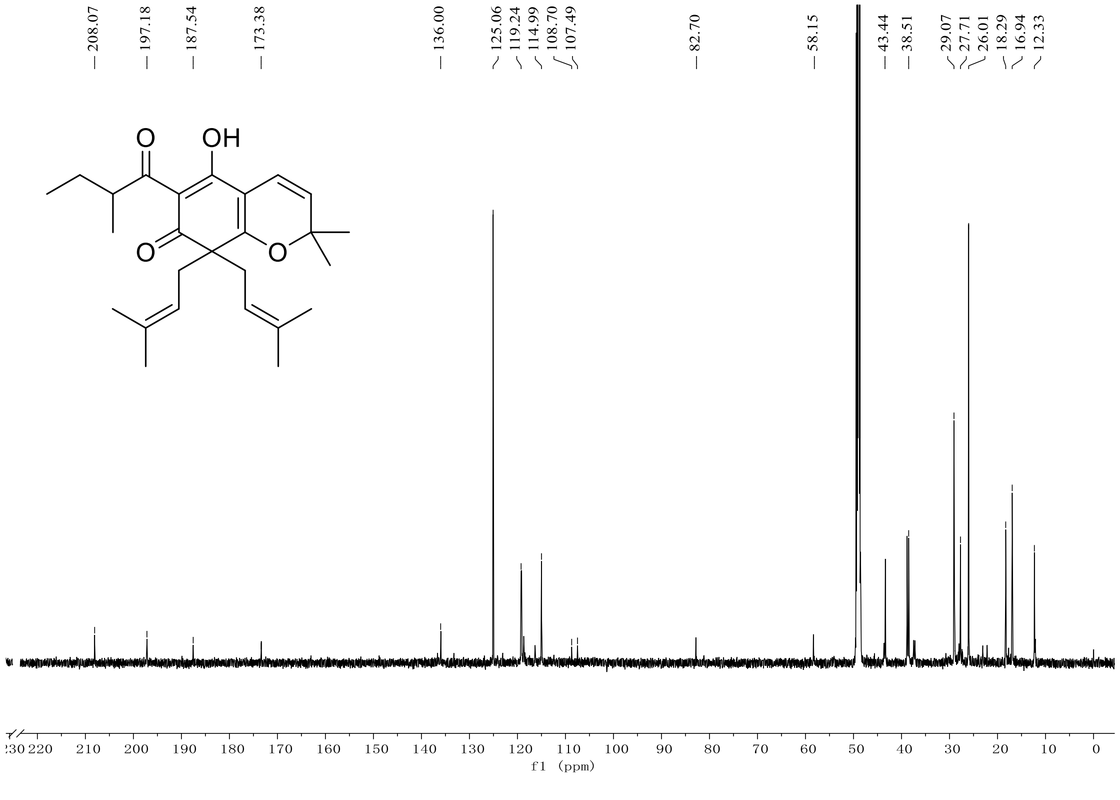

Fig. S24. ^{1}H NMR Spectrum of Compound **2** (400 MHz, $CDCl_3$)

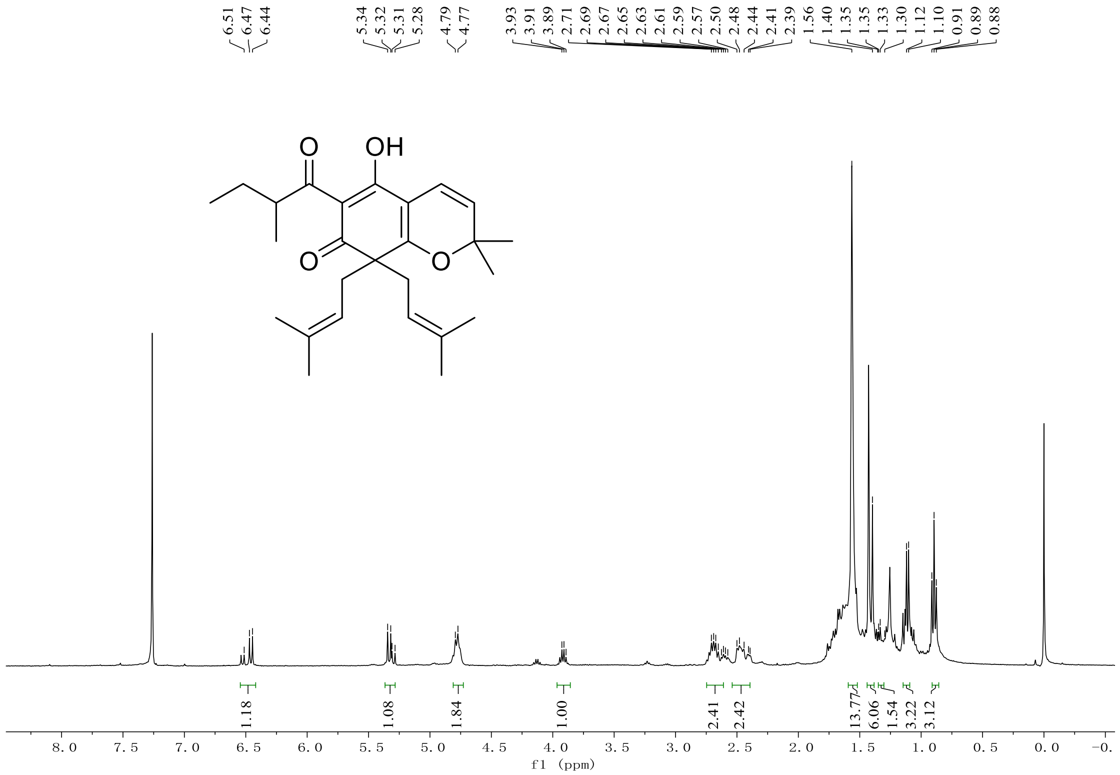

Fig. S25. ^{13}C NMR Spectrum of Compound **2** (150 MHz, $CDCl_3$)

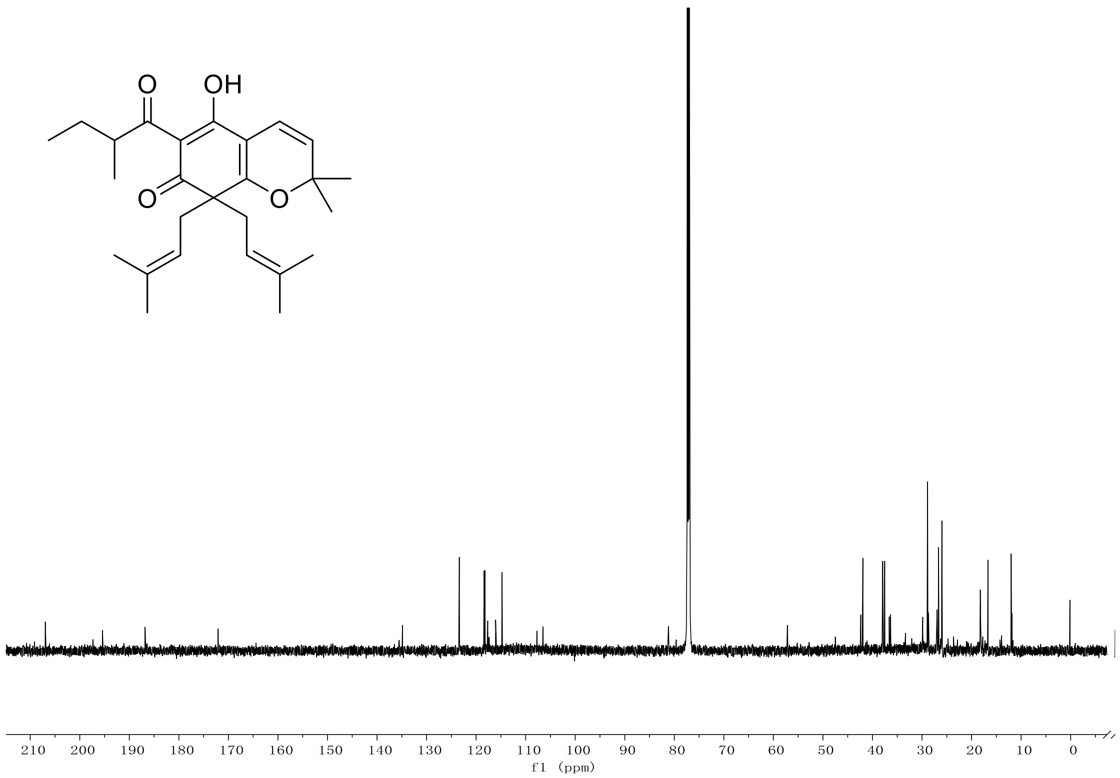

Fig. S26. Dept135 Spectrum of Compound **2** (150 MHz, CD_3OD)

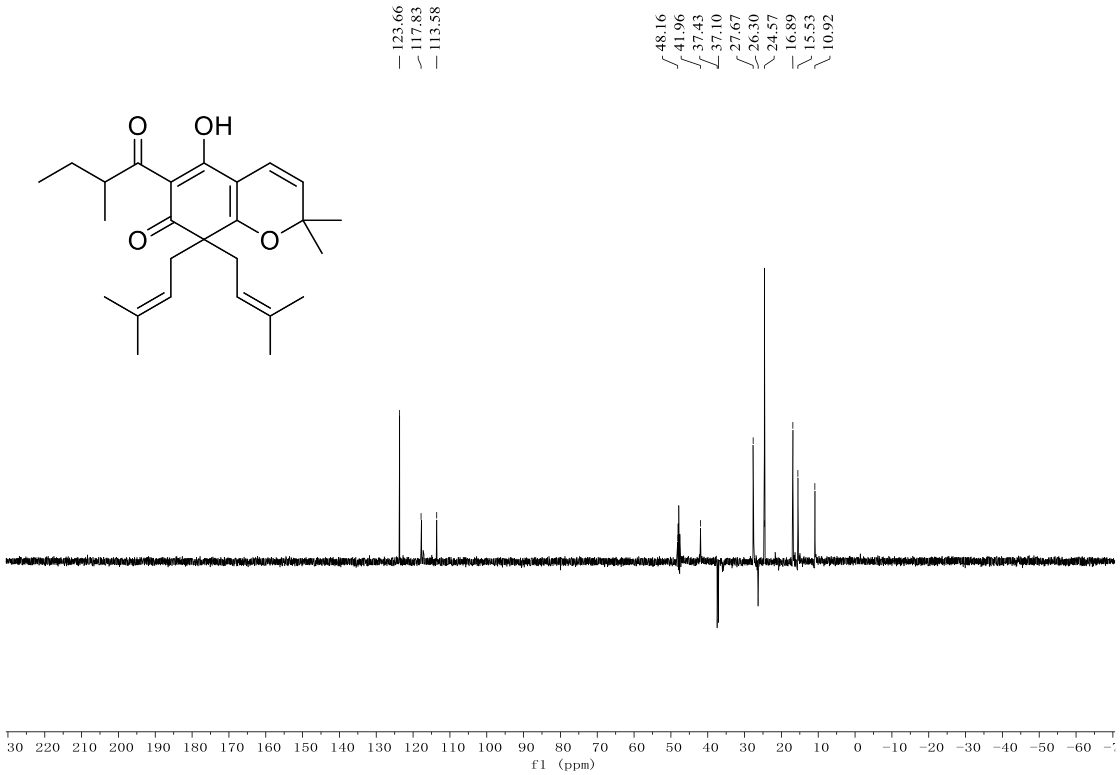

Fig. S27. HMBC Spectrum of Compound 2 (150 MHz, CD_3OD)

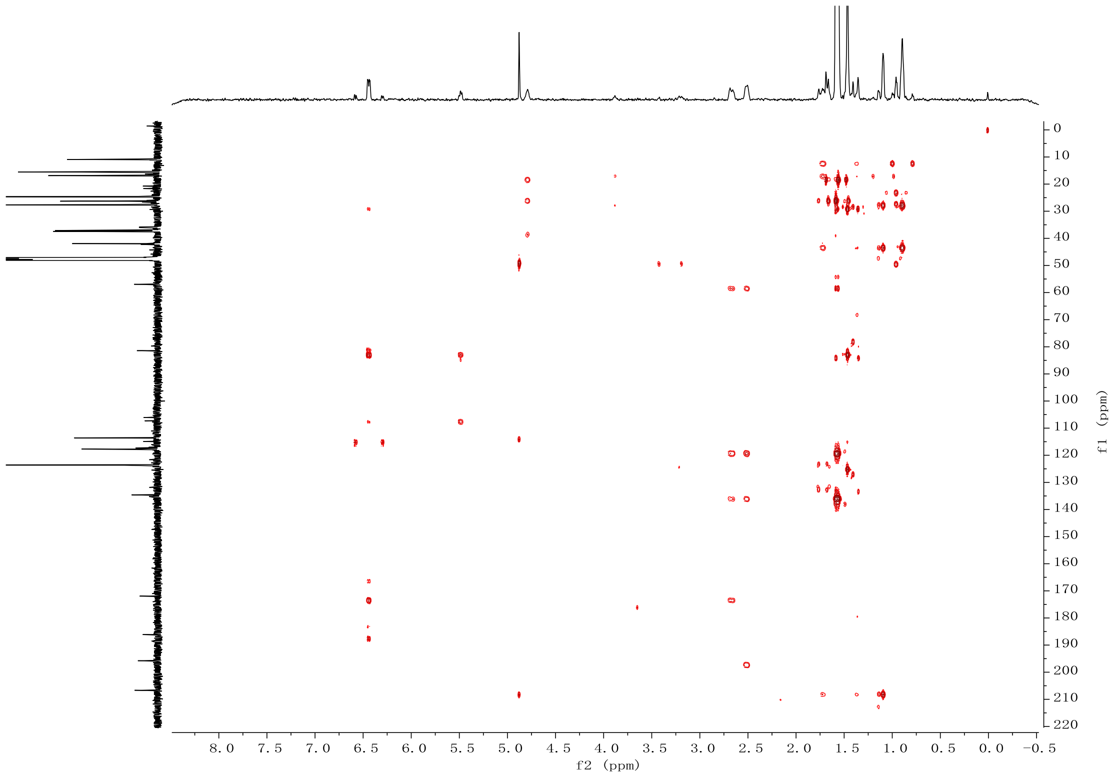

Fig. S28. HSQC Spectrum of Compound **2** (150 MHz, CD_3OD)

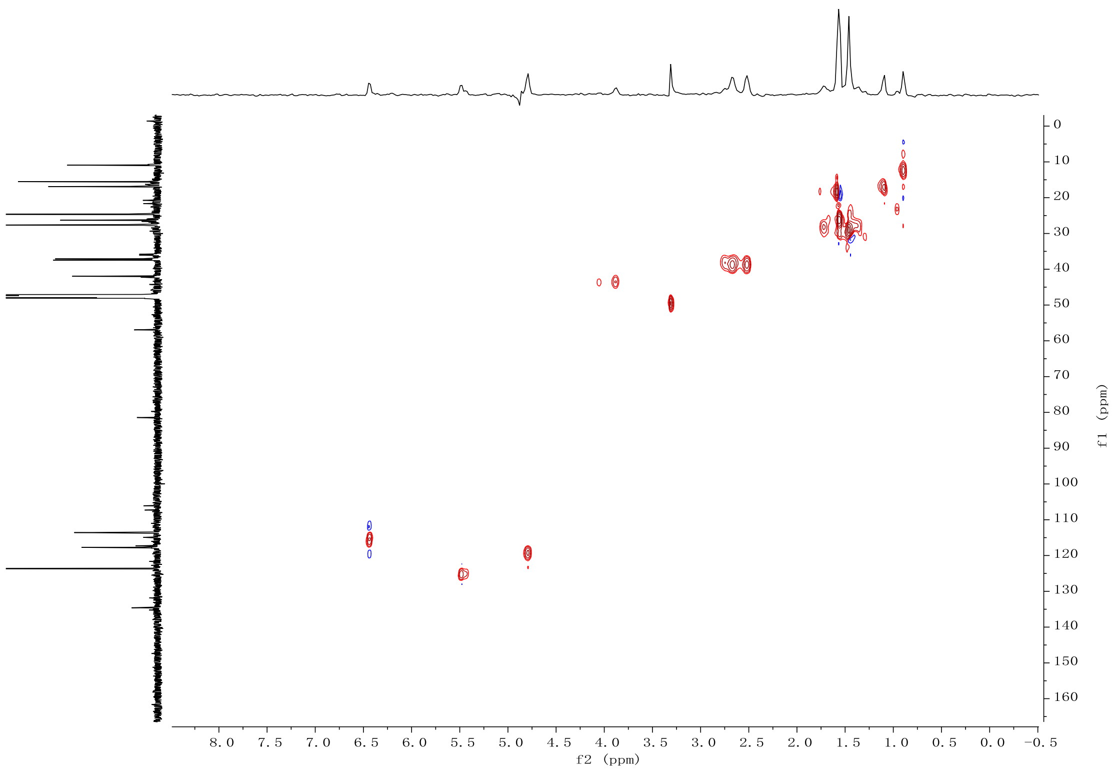

Fig. S29. Cosy Spectrum of Compound **2** (150 MHz, CD_3OD)

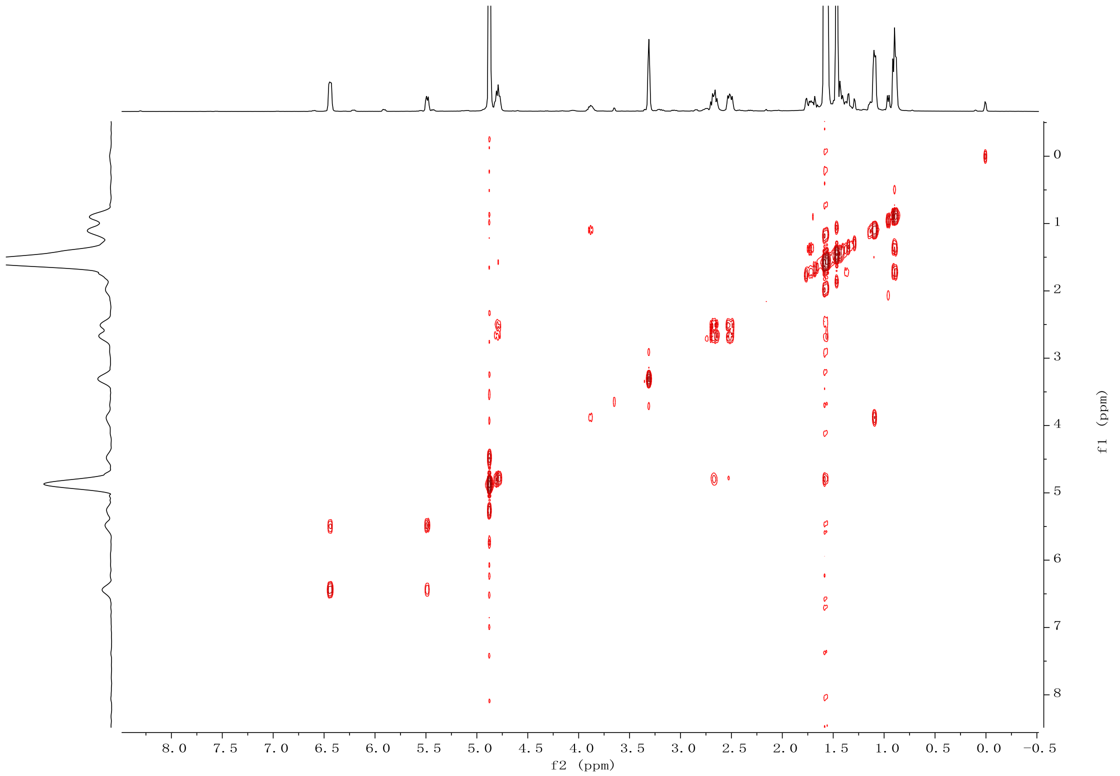

Fig. S30. ^{1}H NMR Spectrum of Compound **7b** (400 MHz, $CDCl_3$)

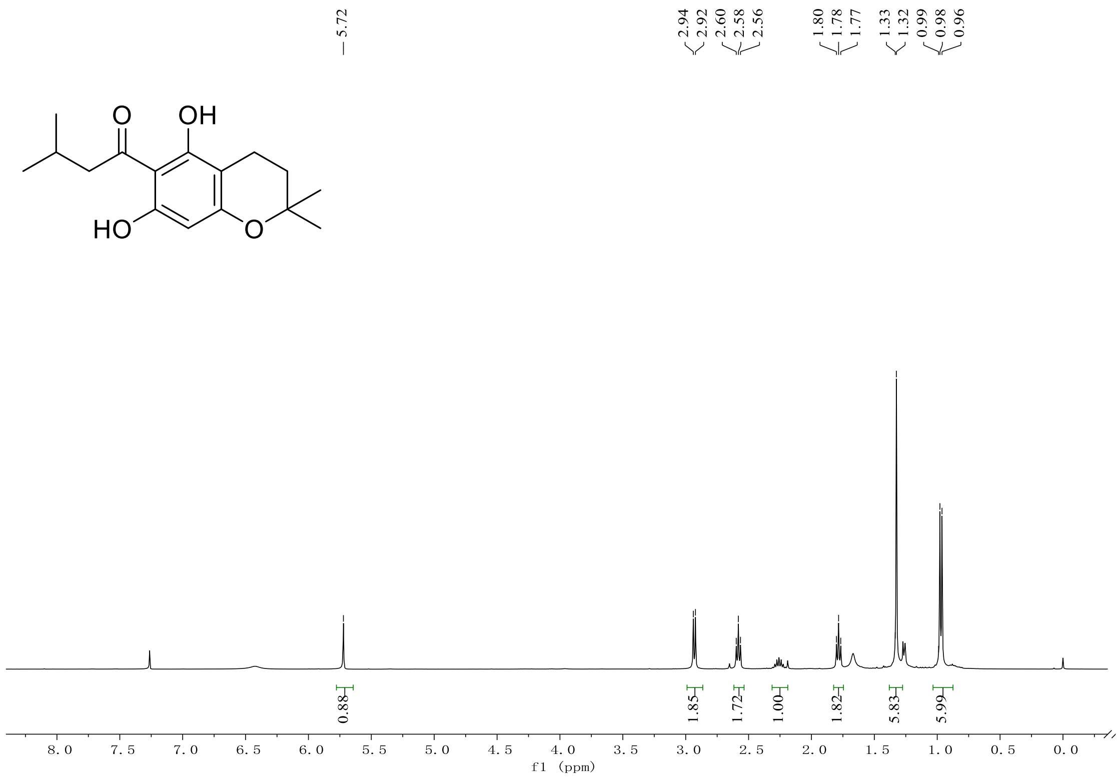

Fig. S31. ^{13}C NMR Spectrum of Compound 7b (150 MHz, $CDCl_3$)

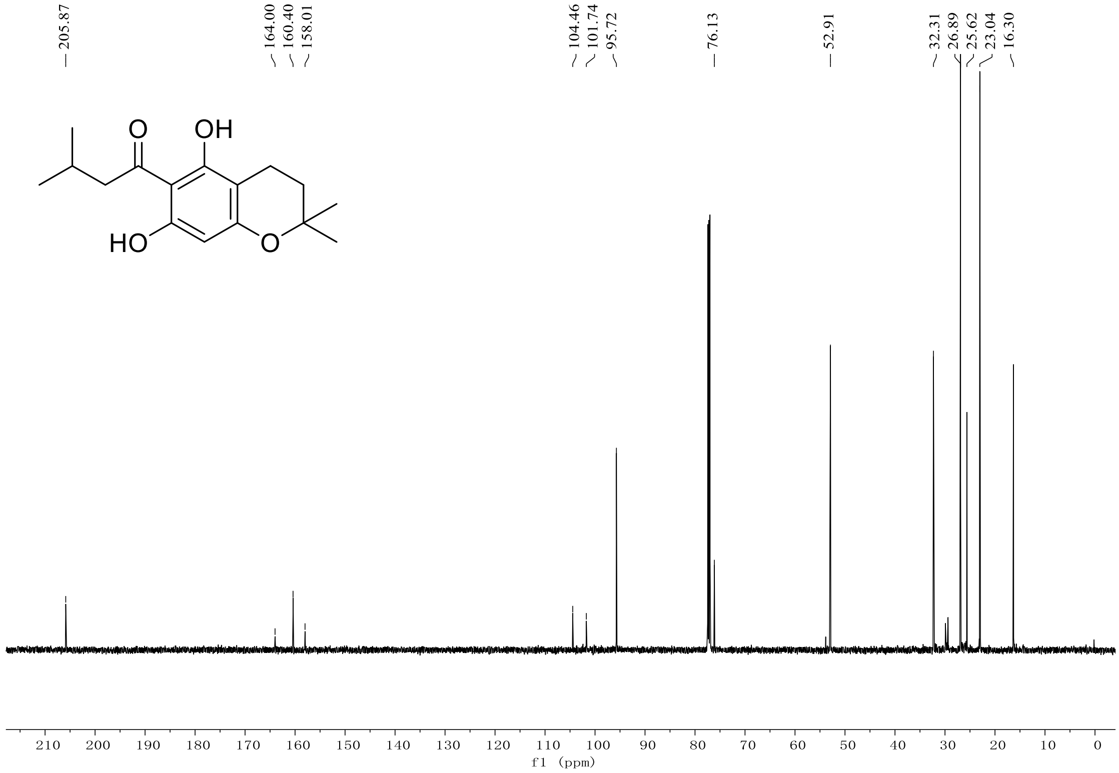

Fig. S32. HMBC Spectrum of Compound **7b** (150 MHz, $CDCl_3$)

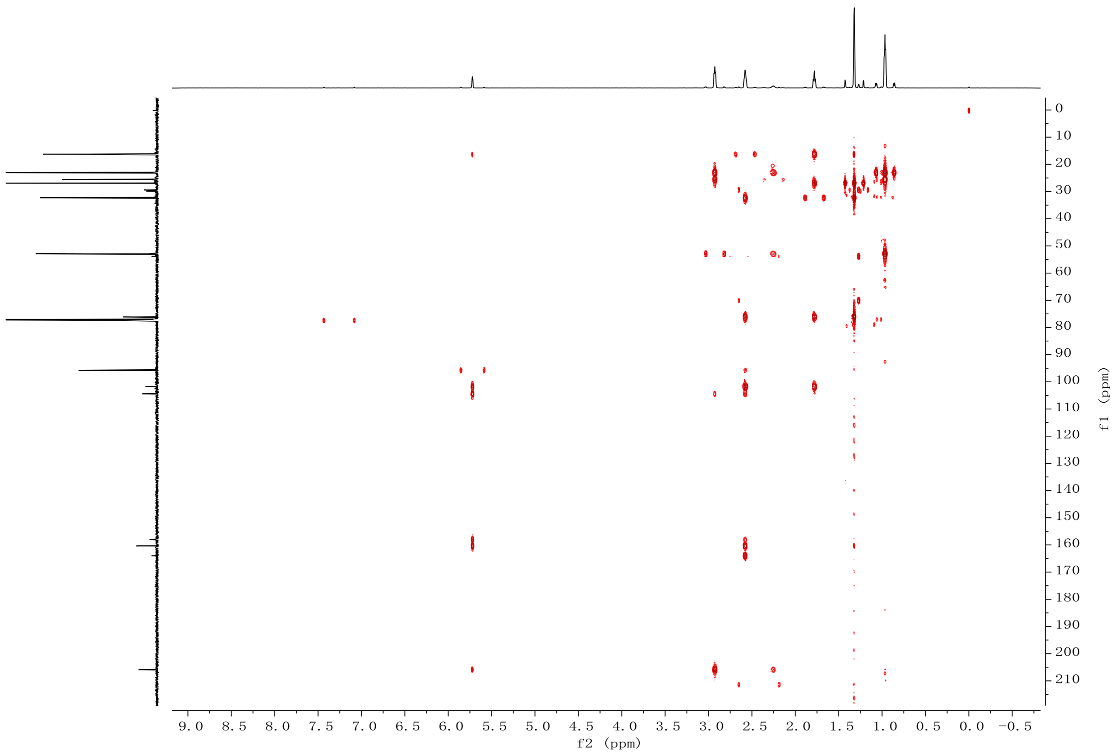

Fig. S33. HSQC Spectrum of Compound **7b** (150 MHz, $CDCl_3$)

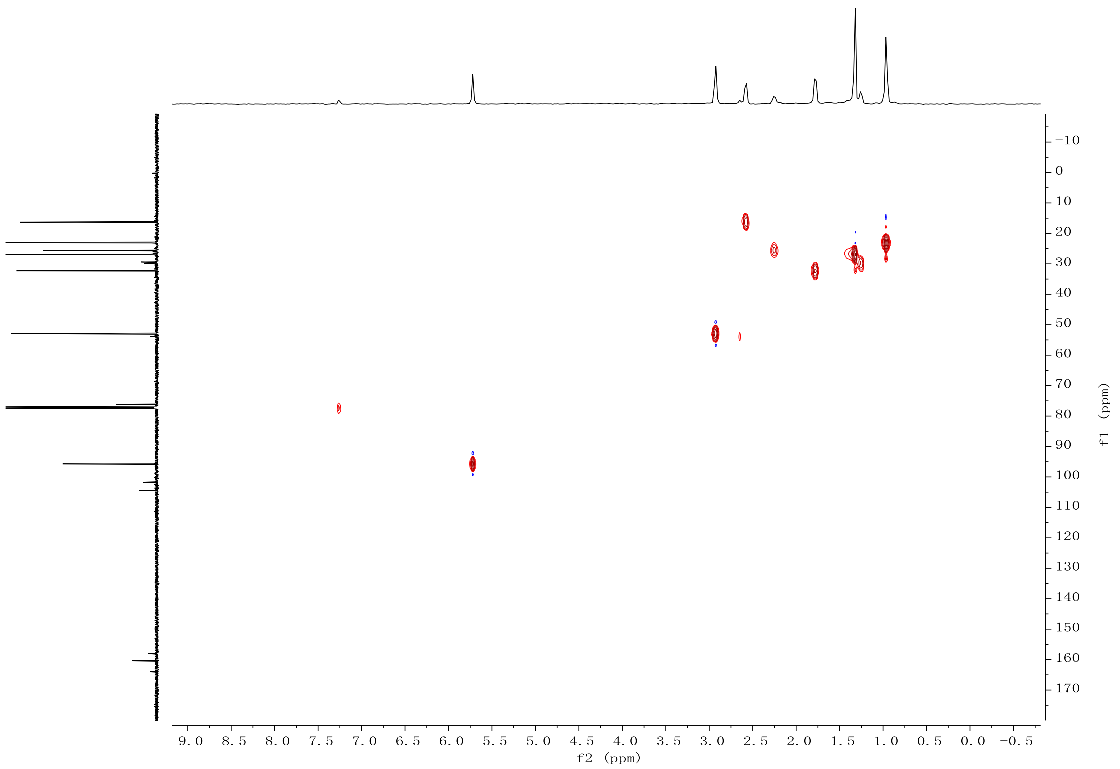

Fig. S34. ^{1}H NMR Spectrum of Compound **7a** (400 MHz, $CDCl_3$)

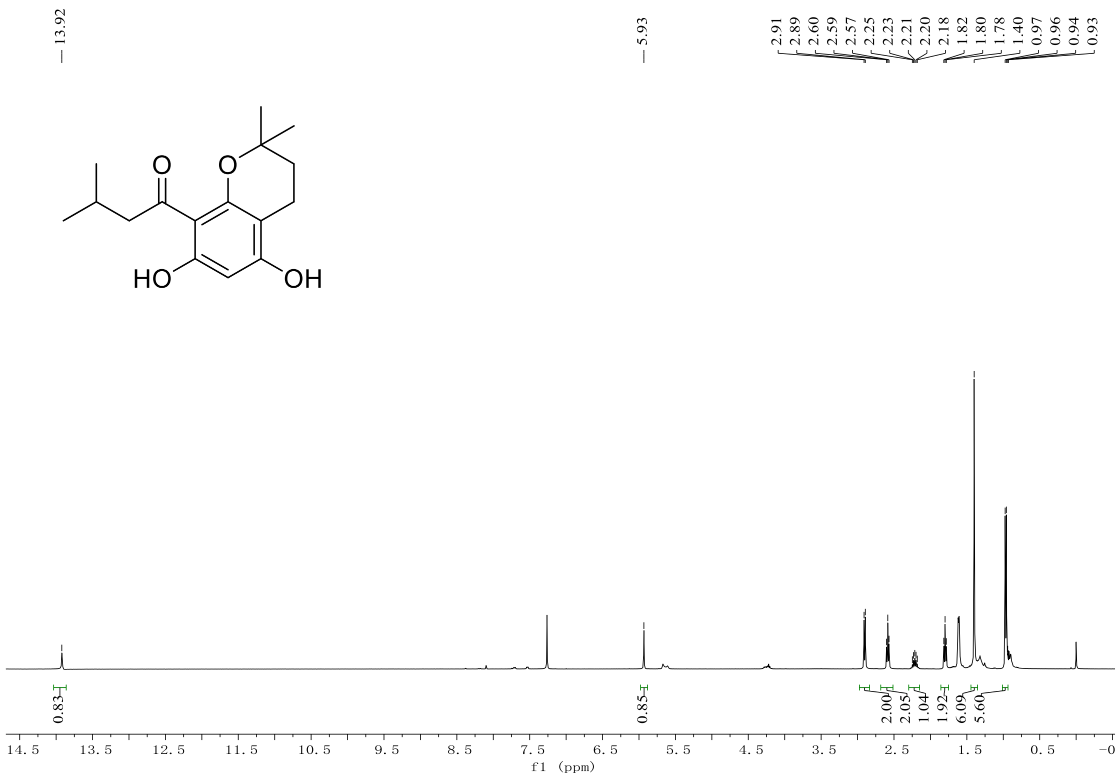

Fig. S35. ^{13}C NMR Spectrum of Compound **7a** (150 MHz, $CDCl_3$)

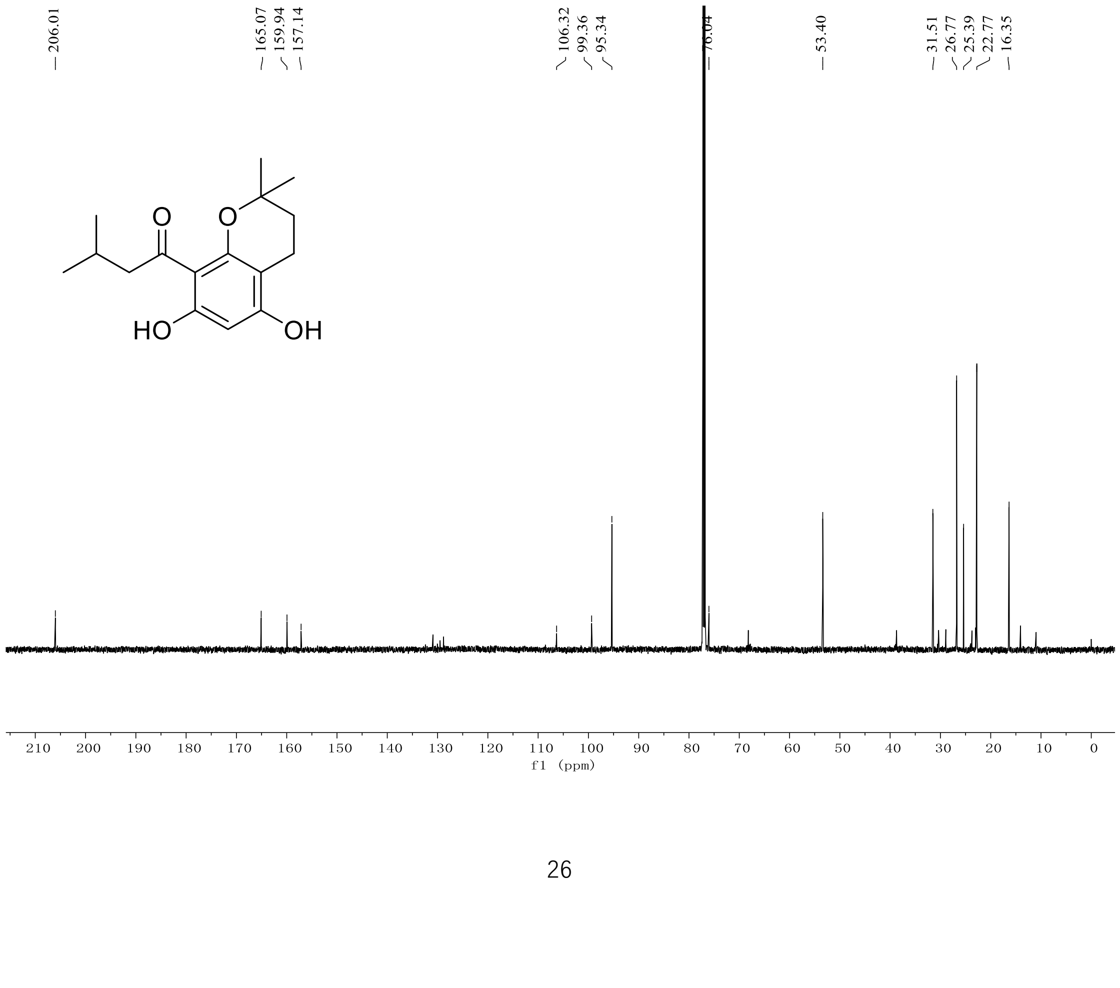

Fig. S36. ^{1}H NMR Spectrum of Compound **8** (400 MHz, CD_3OD)

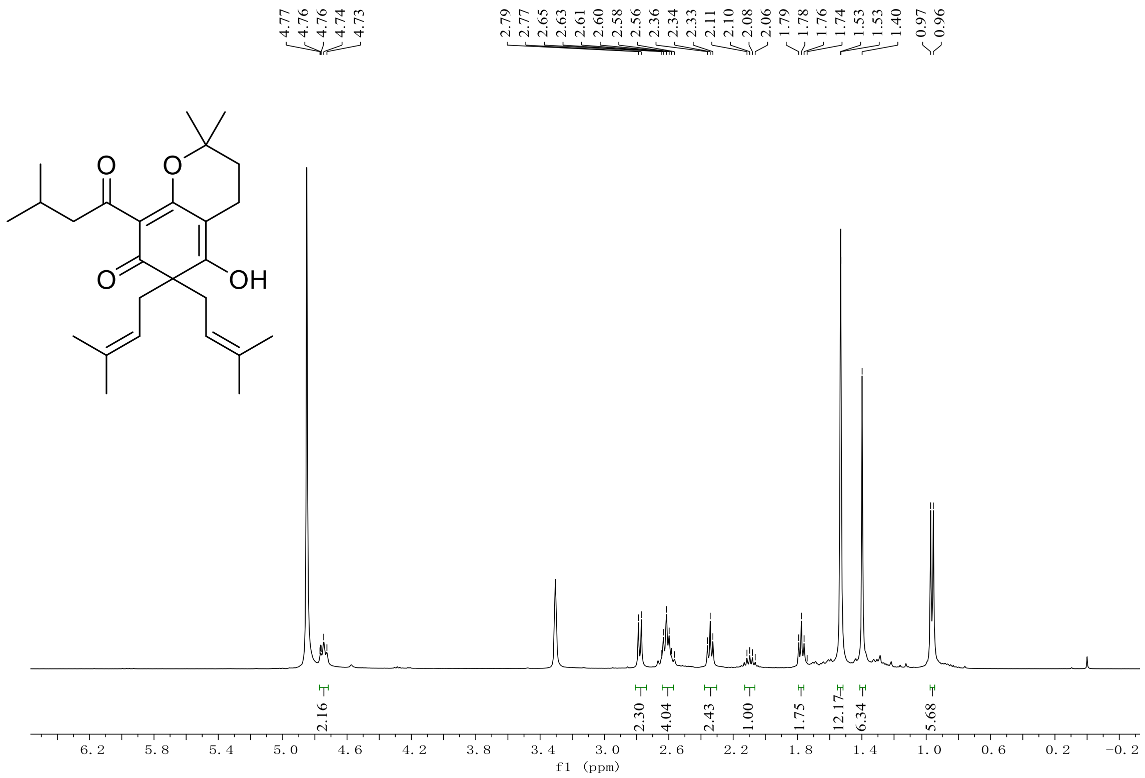

Fig. S37. ^{13}C NMR Spectrum of Compound **8** (150 MHz, CD_3OD)

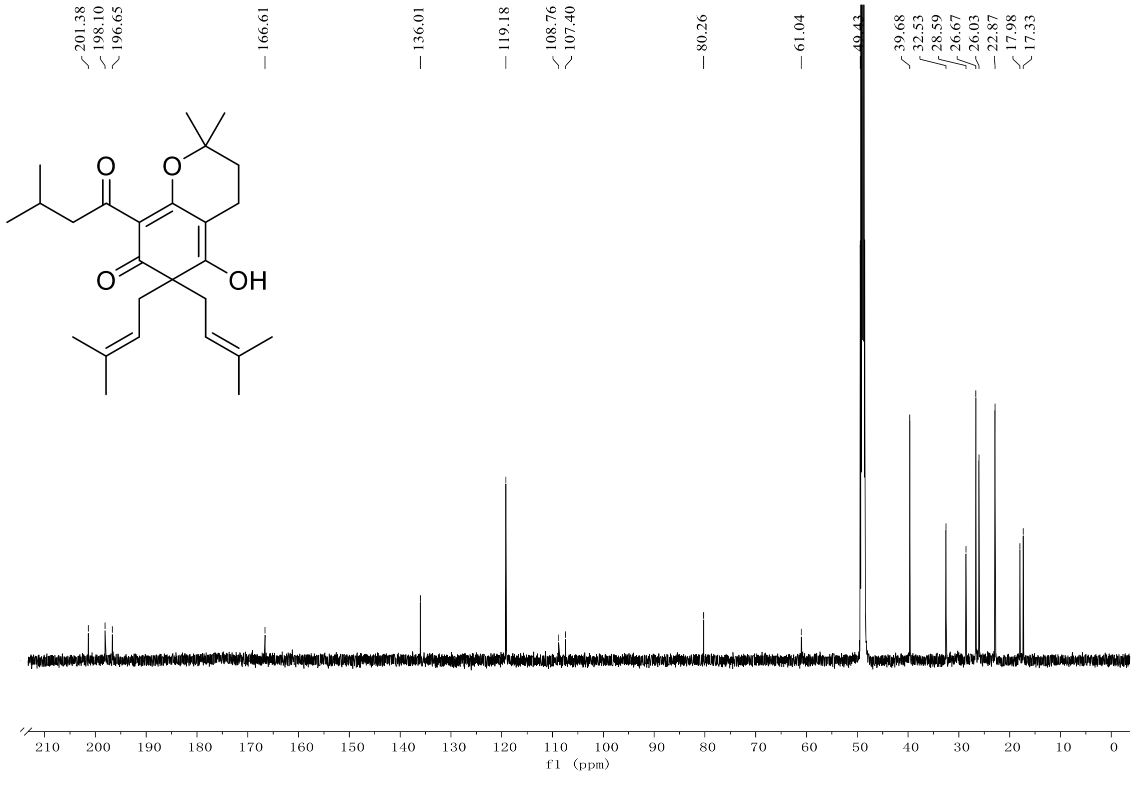

Fig. S38. HMBC Spectrum of Compound **8** (150 MHz, CD_3OD)

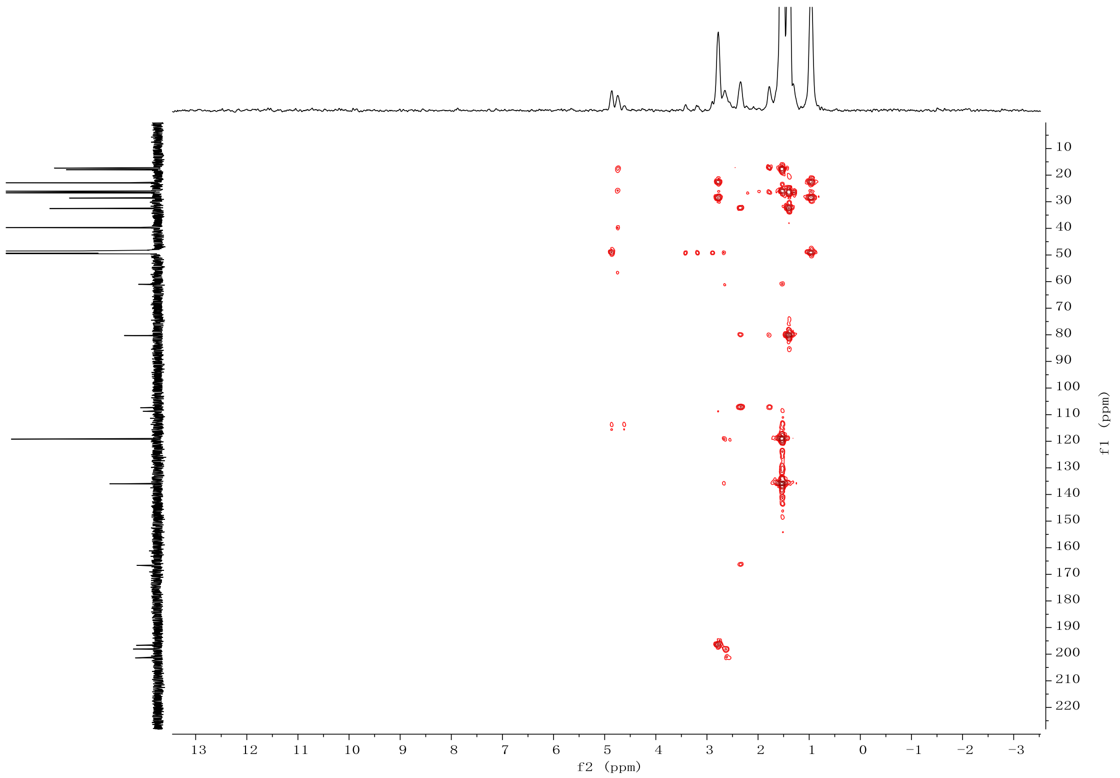

Fig. S39. HSQC Spectrum of Compound **8** (150 MHz, CD_3OD)

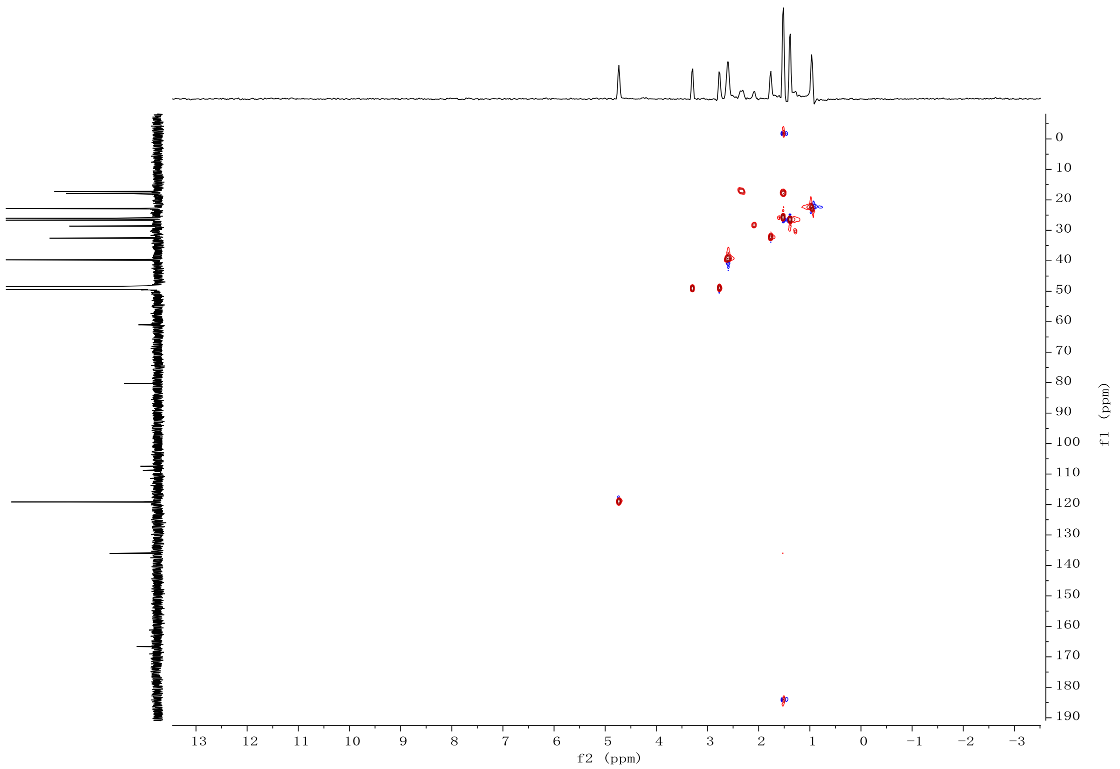

Fig. S40. ^{1}H NMR Spectrum of Compound **9** (400 MHz, CD_3OD)

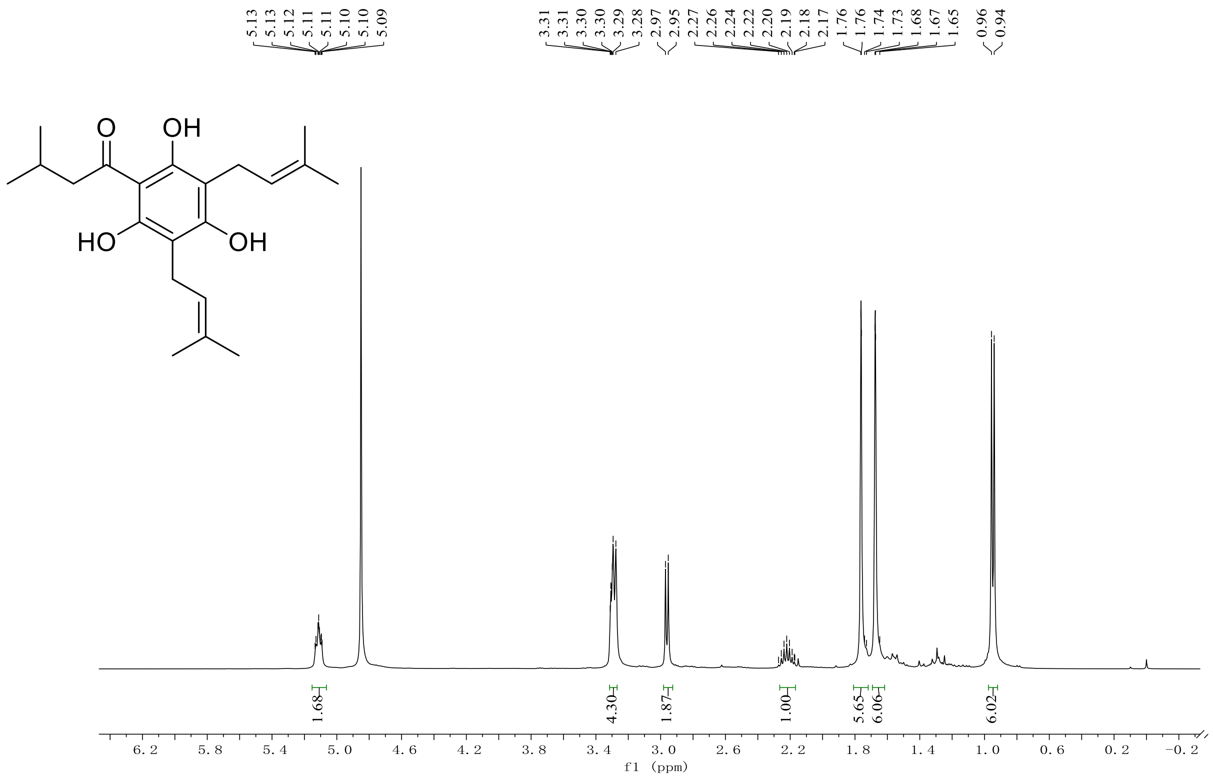

Fig. S41. ^{13}C NMR Spectrum of Compound **9** (150 MHz, CD_3OD)

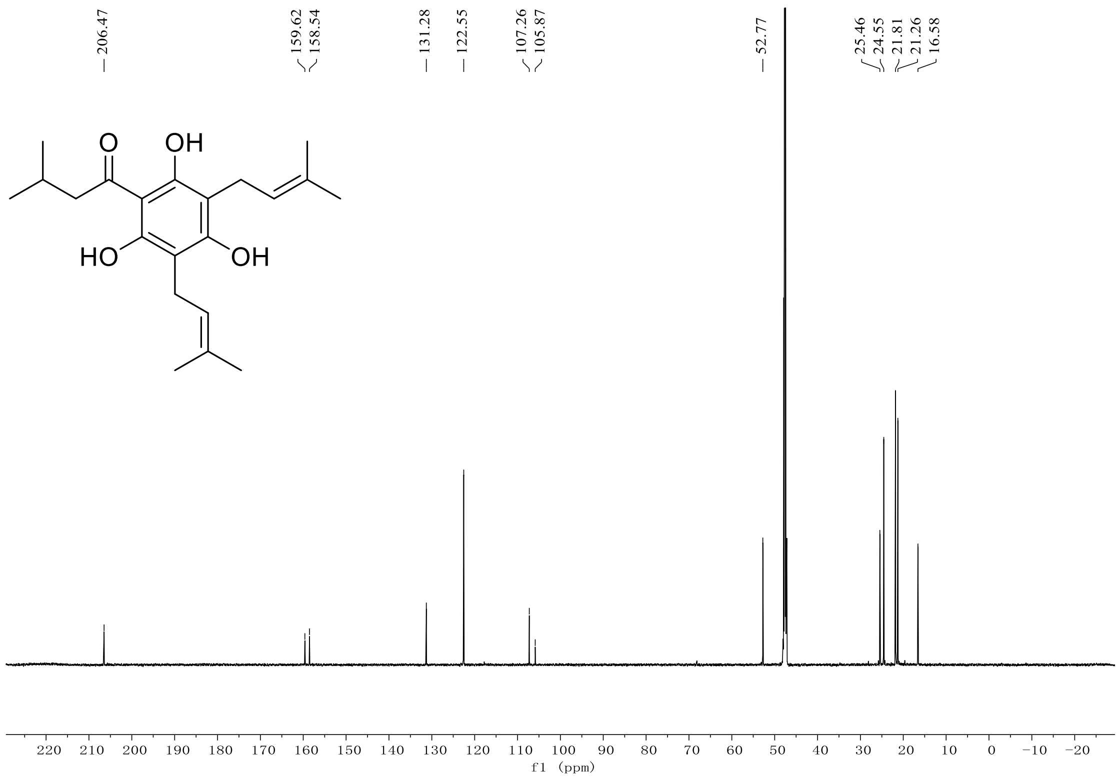

Fig. S42. ^{1}H NMR Spectrum of Compound **10** (400 MHz, CD_3OD)

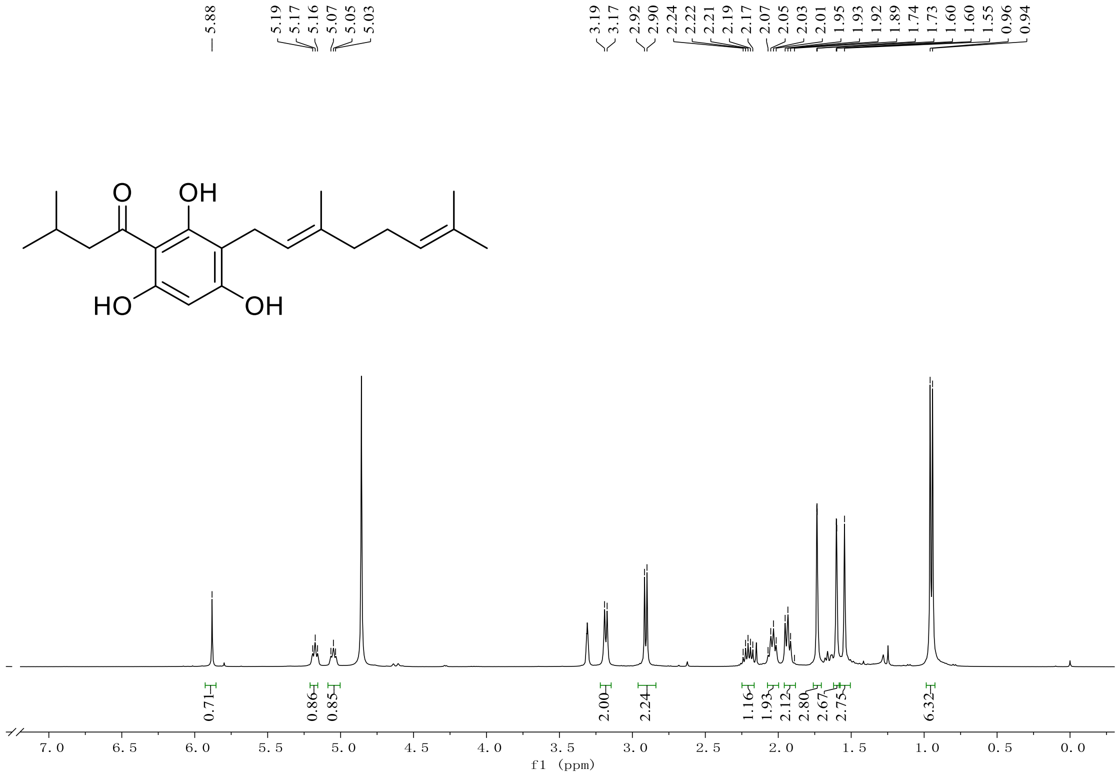

Fig. S43. ^{13}C NMR Spectrum of Compound **10** (150 MHz, CD_3OD)

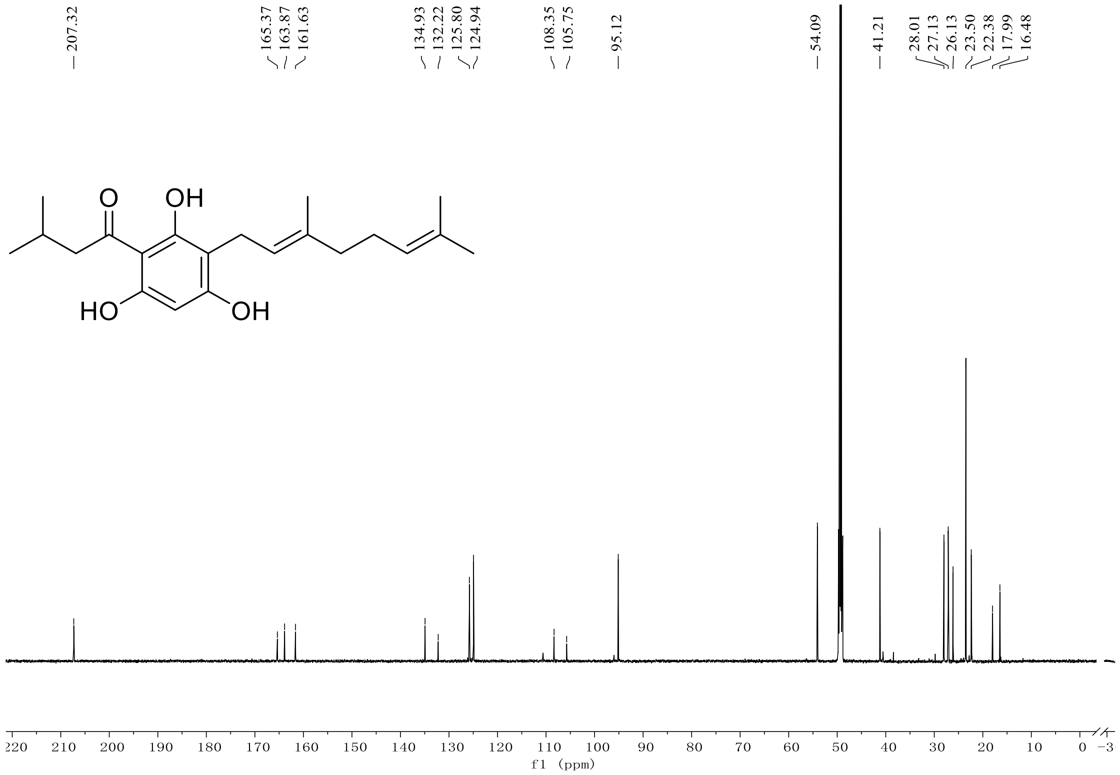

Fig. S44. ^{1}H NMR Spectrum of Compound **11** (400 MHz, $CDCl_3$)

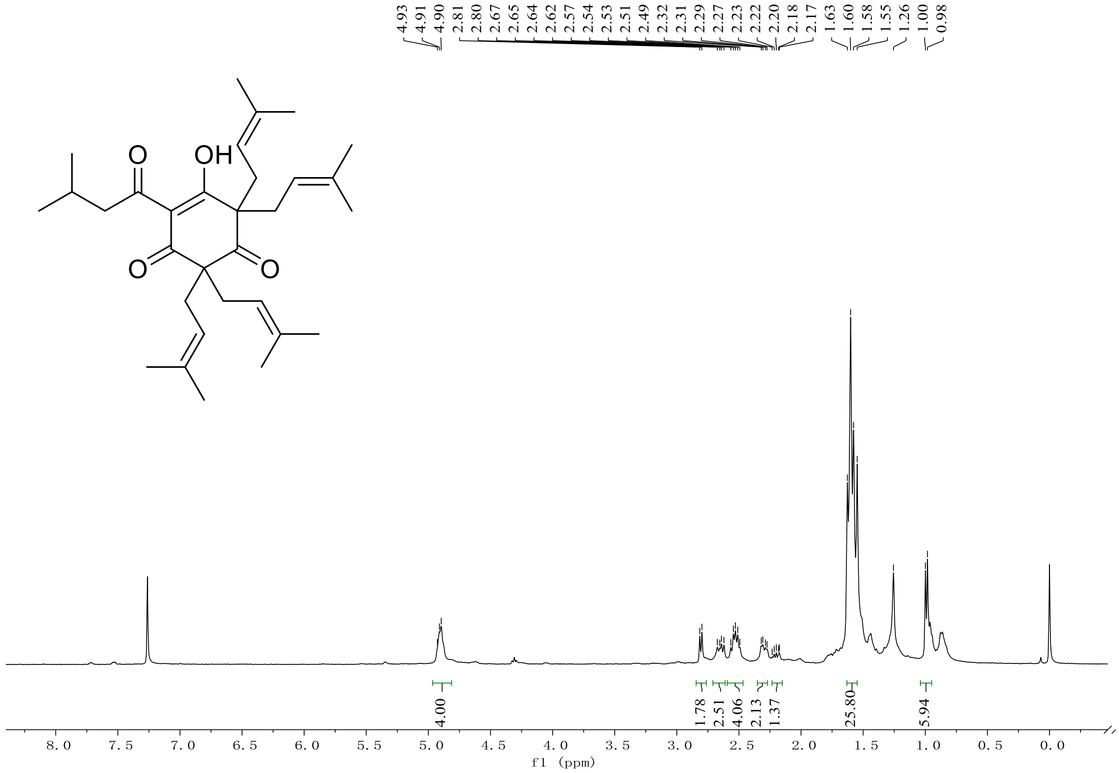

Fig. S45. ^{13}C NMR Spectrum of Compound **11** (150 MHz, $CDCl_3$)

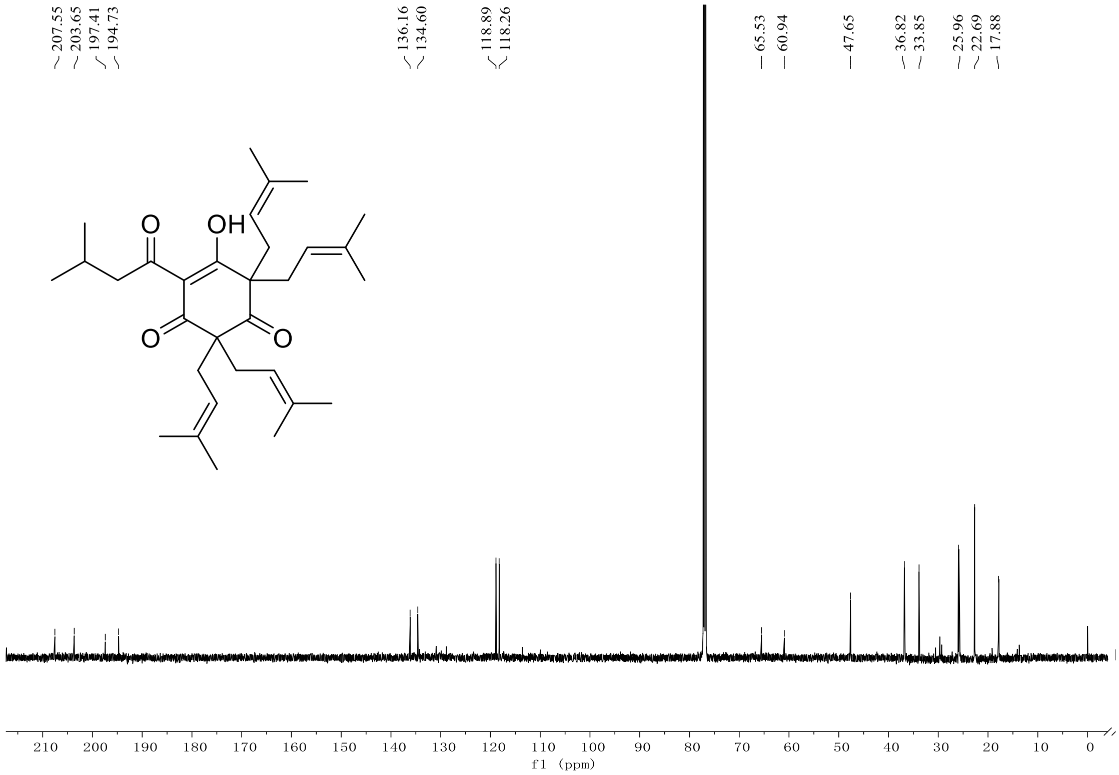

Fig. S46. ^{1}H NMR Spectrum of Compound **12** (400 MHz, CD_3OD)

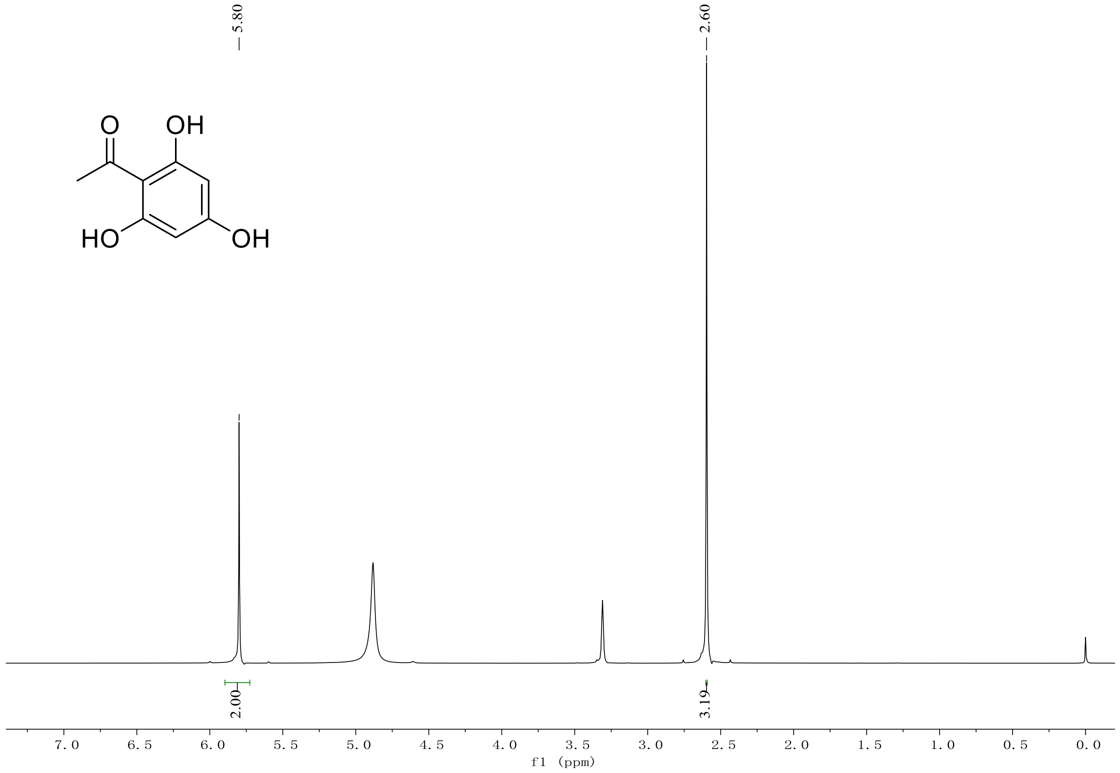

Fig. S47. ^{13}C NMR Spectrum of Compound **12** (150 MHz, CD_3OD)

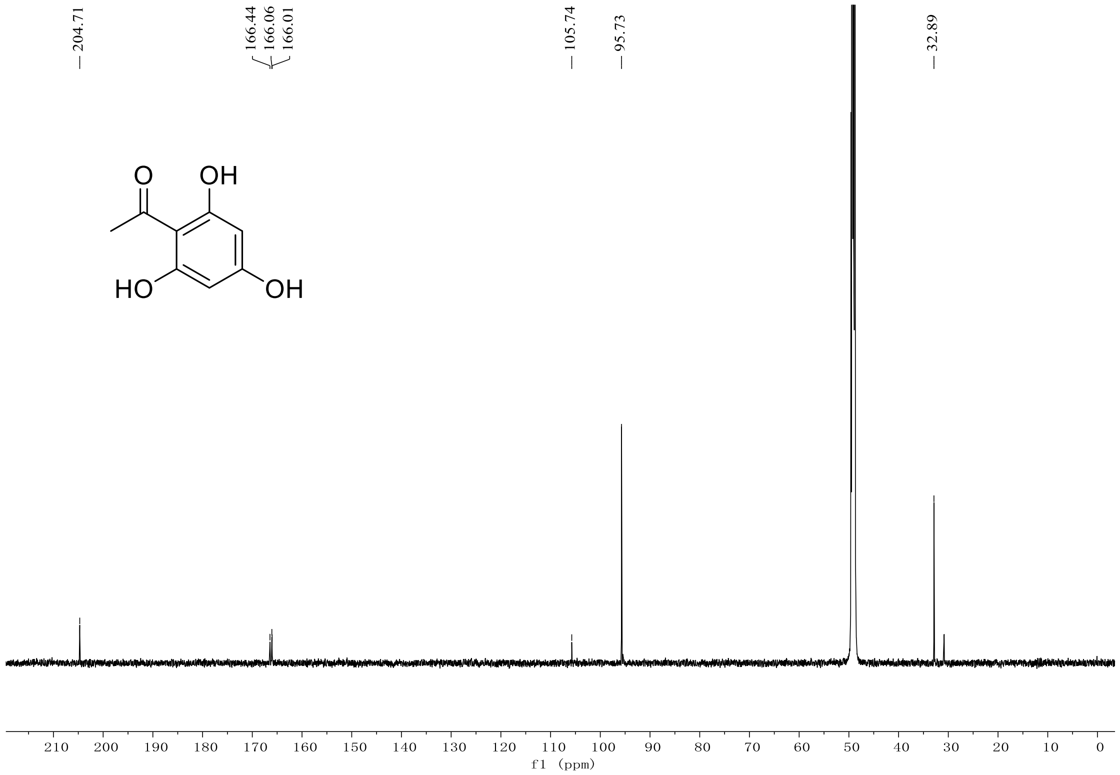

Fig. S48. ^{1}H NMR Spectrum of Compound **13** (400 MHz, CD_3OD)

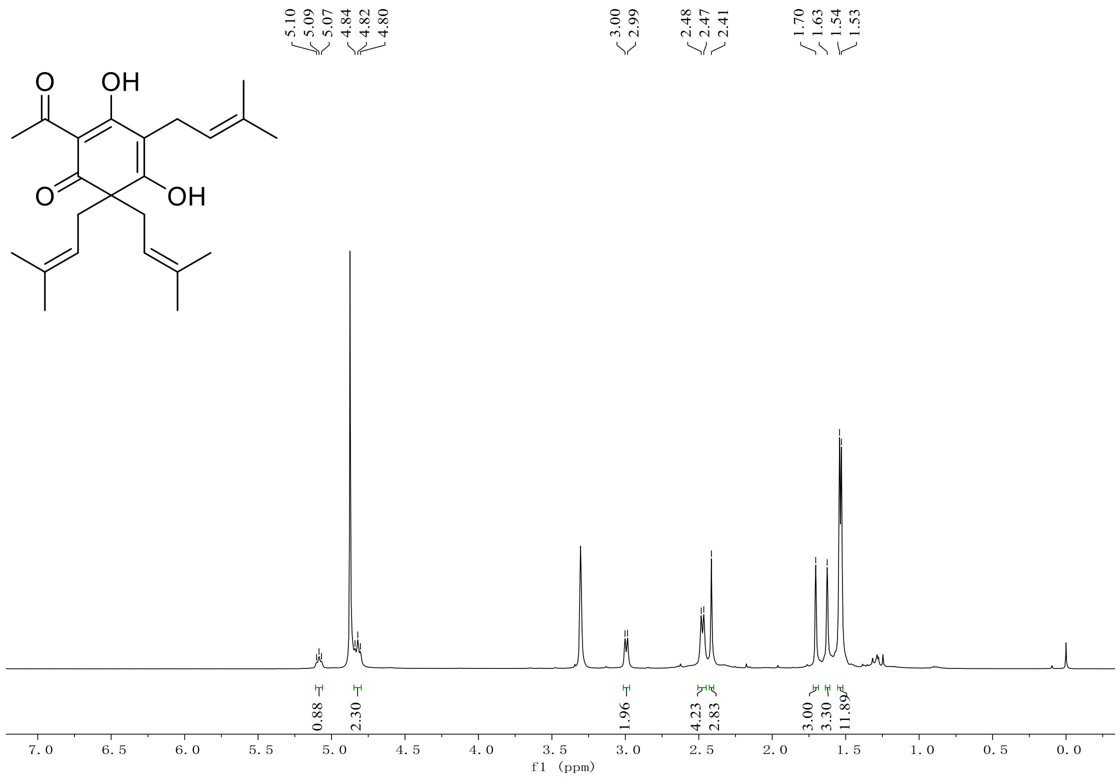

Fig. S49. ^{13}C NMR Spectrum of Compound **13** (150 MHz, CD_3OD)

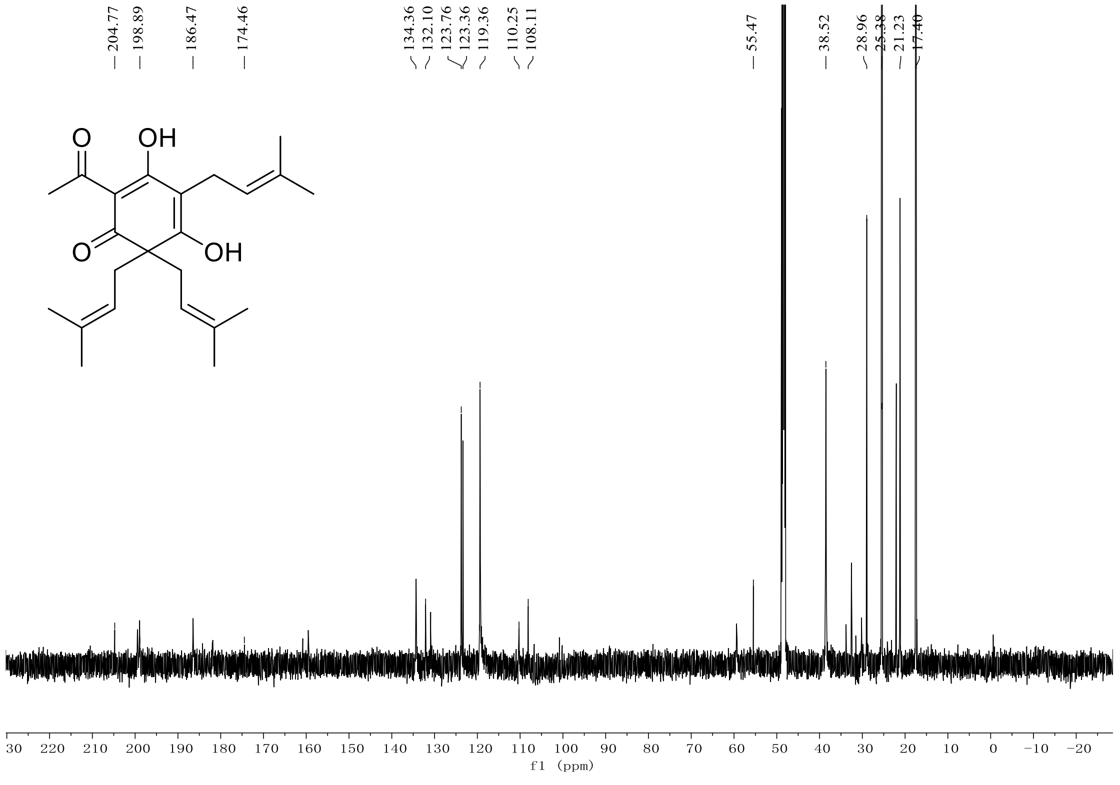

Fig. S50. ^{1}H NMR Spectrum of Compound **14** (400 MHz, $CDCl_3$)

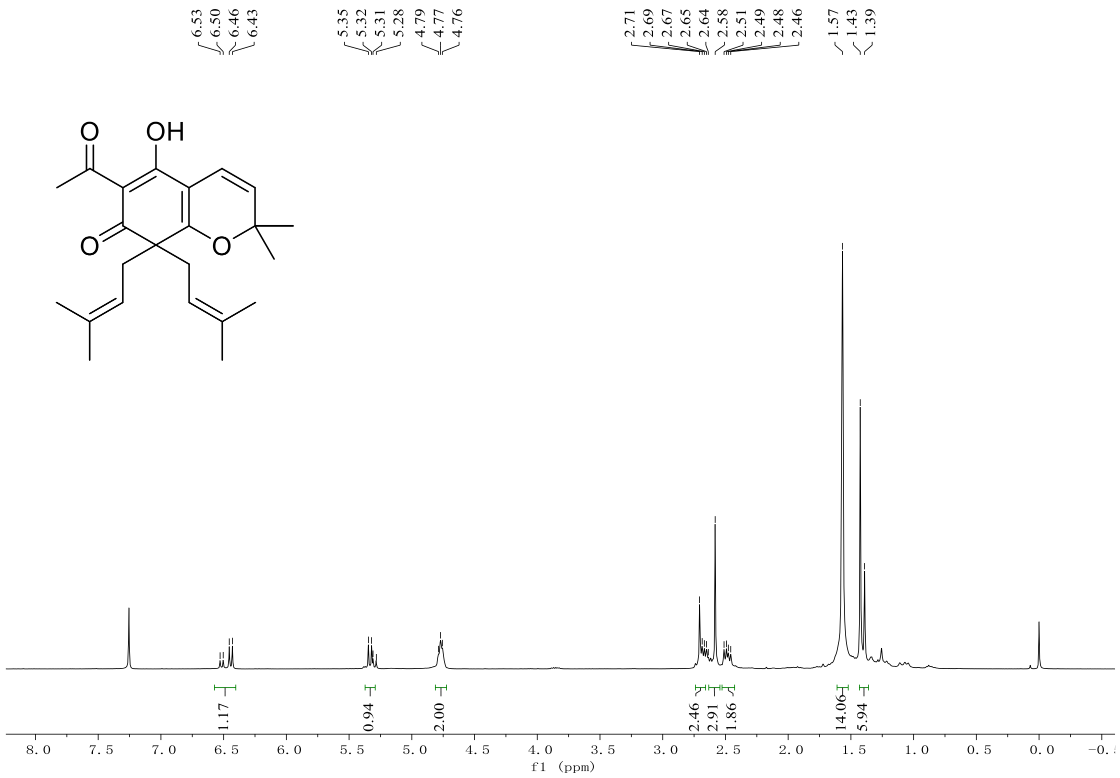

Fig. S51. ^{13}C NMR Spectrum of Compound **14** (150 MHz, $CDCl_3$)

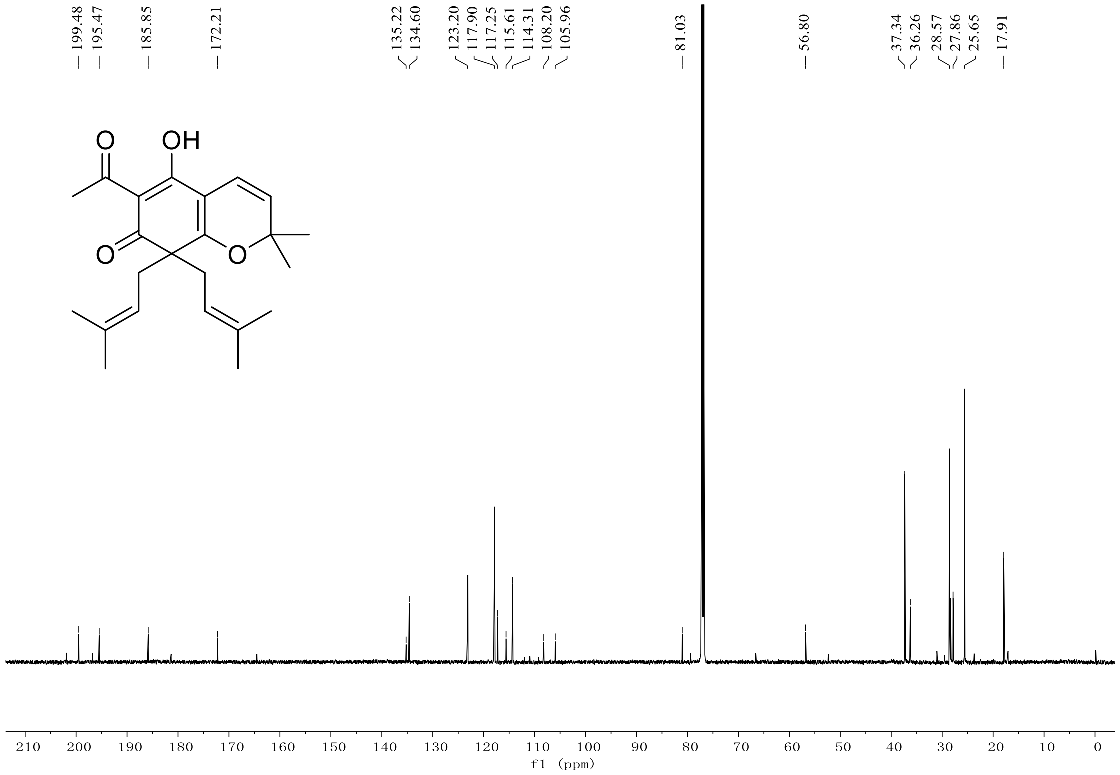

Fig. S52. HMBC Spectrum of Compound **14** (600 MHz, $CDCl_3$)

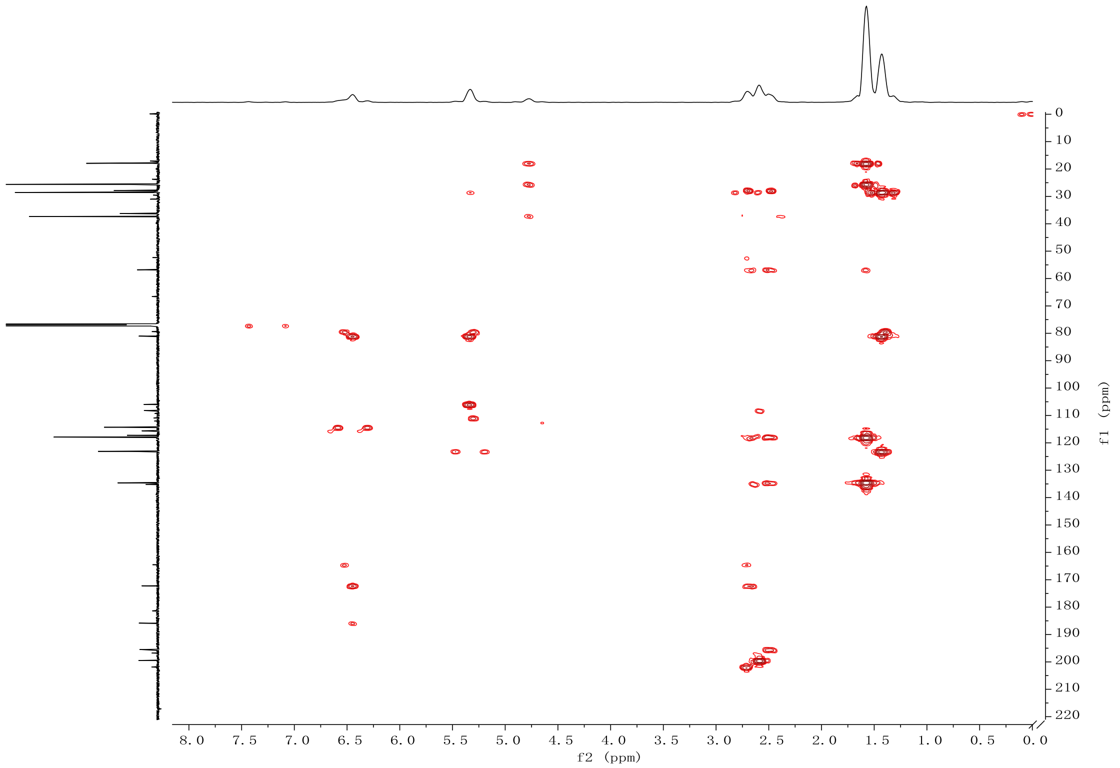

Fig. S53. HSQC Spectrum of Compound **14** (600 MHz, $CDCl_3$)

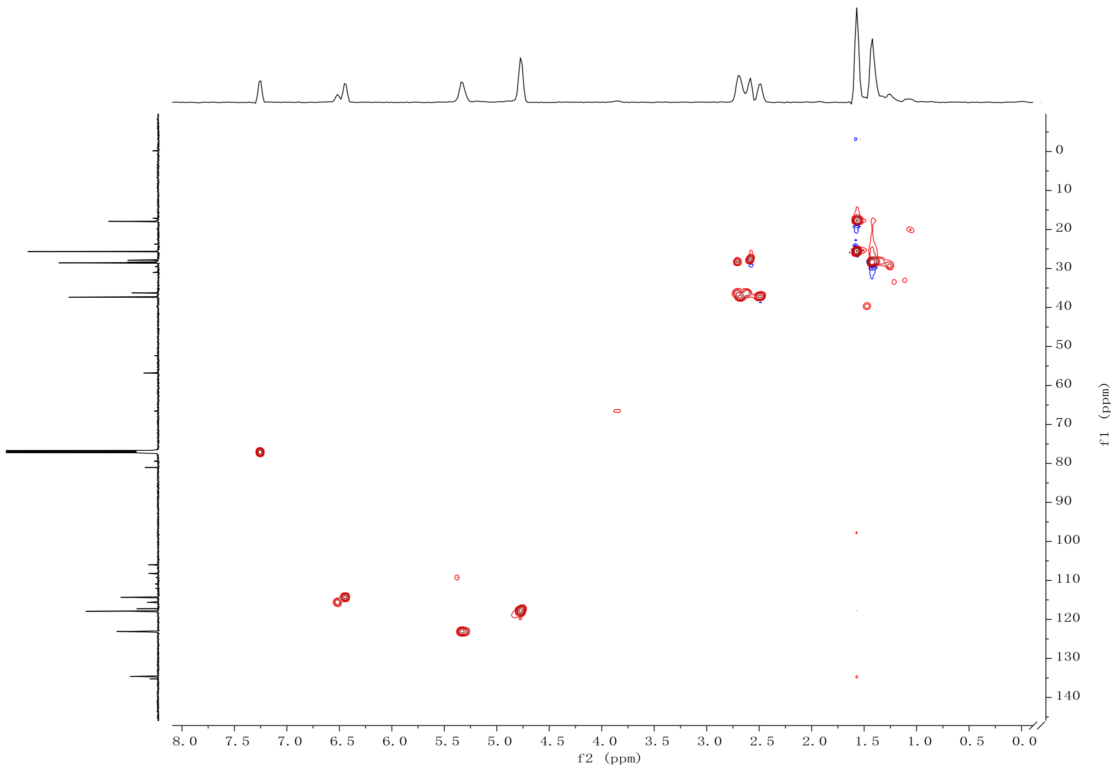

4. Comparison of NMR data of natural 1 and synthetic 1

No.	Natural **1** (600 MHz)		Synthetic **1** (400 MHz)	
	δ_C (ppm)	δ_H (ppm, J in Hz)	δ_C (ppm)	δ_H (ppm, J in Hz)
1	107.6		107.3	
2	187.5		187.2	
3	109.2		109.0	
4	173.4		173.3	
5	58.2		58.0	
6	197.3		197.0	
7	203.5		203.2	
8	49.4	2.91 d (7.0)	49.2	2.89 d (7.0)
9	27.3	2.07 dq (13.4, 6.4)	27.0	2.06 dq (13.8, 6.4)
10	22.9	0.94 s	22.8	0.96 s
11	23.1	0.95 s	22.9	0.95 s
1’	114.9	6.44 d (10.1)	114.7	6.42 d (10.1)
2’	125.1	5.45 d (10.1)	124.8	5.44 d (10.1)
3’	82.8		82.6	
4’	28.9	1.45 s	28.8	1.45 s
5’	28.9	1.42 s	28.8	1.43 s
12	38.7	2.51 dd (13.9, 7.3)	38.4	2.51 dd (14.2, 7.3)
13	119.2	4.75 t (7.2)	118.9	4.75 t (7.2)
14	136.0		135.7	
15	18.3	1.57 s	18.0	1.56 s
16	25.9	1.56 s	25.7	1.55 s
17	38.7	2.65 dd (13.8, 7.8)	38.4	2.65 dd (13.8, 7.3)
18	119.2	4.80 t (7.2)	118.9	4.80 t (7.2)
19	136.0		135.7	
20	18.3	1,57 s	18.0	1.56 s
21	25.9	1.56 s	25.7	1.55 s

Tested in methanol-d_4.

Comparison of ^{1}H NMR spectrum of natural **1** (600 MHz) and synthetic **1** (400 MHz)

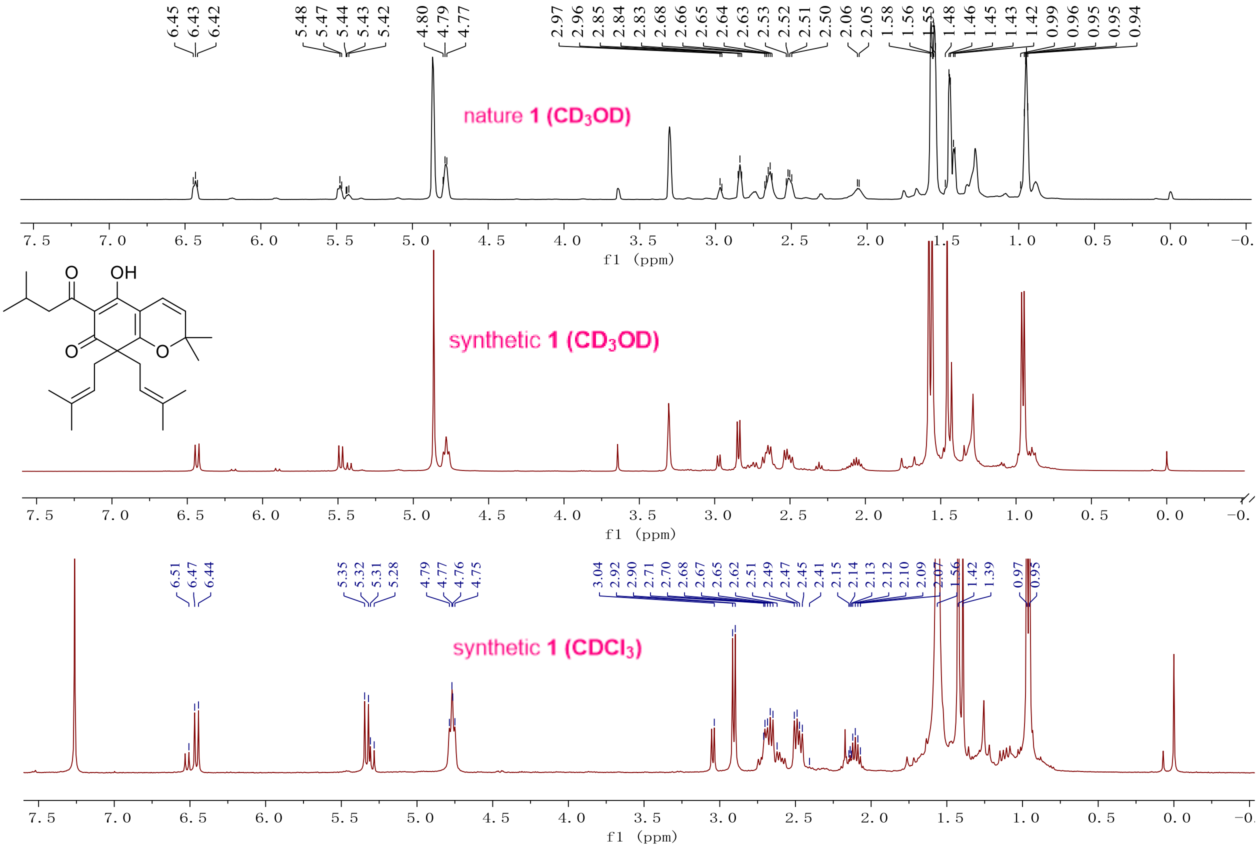

Comparison of ^{13}C NMR spectrum of natural **1** (150 MHz) and synthetic **1** (150 MHz)

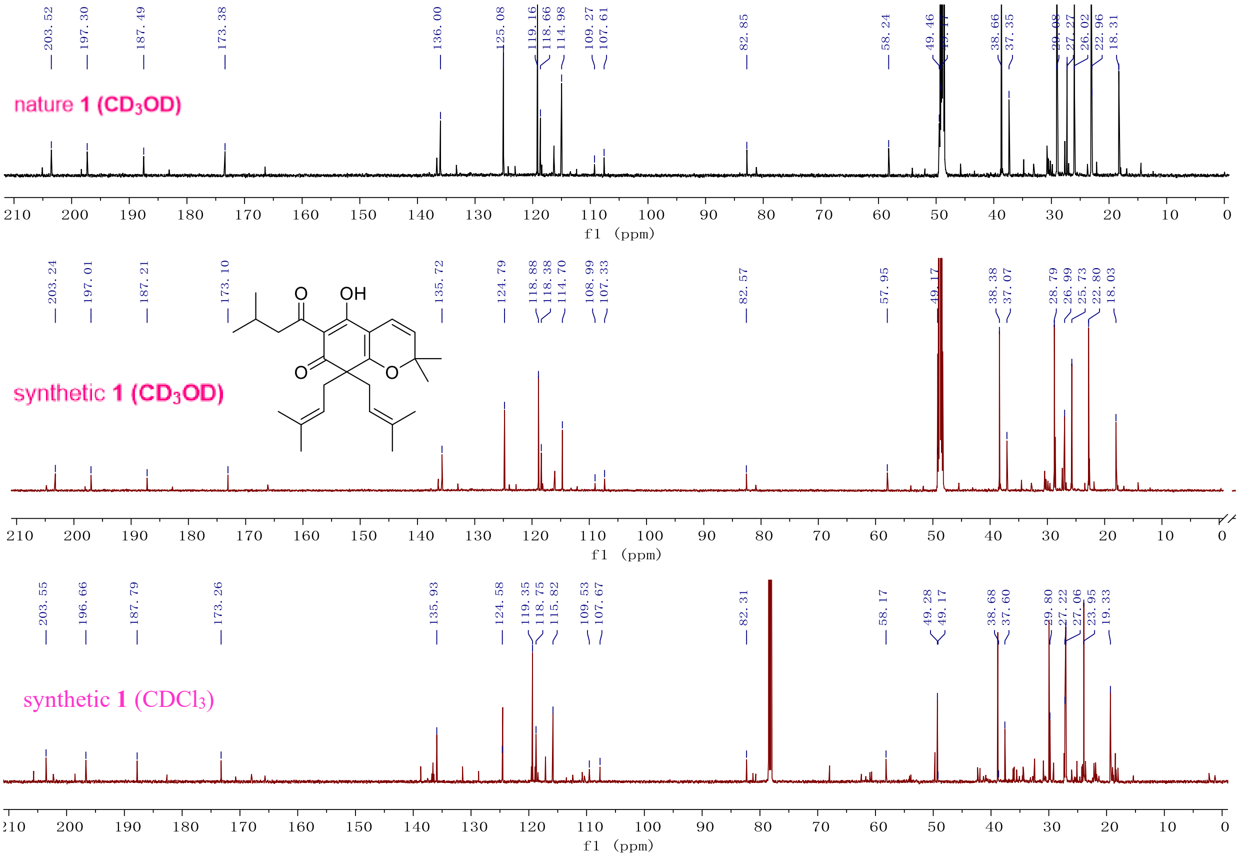

5. Comparison of NMR data of natural 2 and synthetic 2

No.	Natural **2** (600 MHz)		Synthetic **2** (400 MHz)	
	δ_C (ppm)	δ_H (ppm, J in Hz)	δ_C (ppm)	δ_H (ppm, J in Hz)
1	107.5		107.2	
2	187.5		187.3	
3	108.7		108.4	
4	173.4		173.1	
5	58.2		58.0	
6	197.2		197.0	
7	208.1		207.8	
8	43.4	3.87 m	43.4	3.87 m
9	16.9	1.09 d (6.9)	16.7	1.08 d (6.9)
10	27.7	1.74 m	27.7	1.74 m
		1.34 overlap		1.34 overlap
11	12.3	0.90 m	12.0	0.89 m
1’	115.0	6.44 d (10.1)	114.7	6.44 d (10.1)
2’	125.1	5.48 d (10.2)	124.8	5.48 d (10.2)
3’	83.0		82.6	
4’	29.1	1.46 s	28.9	1.46 s
5’	29.1	1.46 s	28.9	1.46 s
12	38.5	2.51 dd (13.9, 7.4)	38.2	2.51 dd (13.9, 7.4)
13	119.2	4.78 t (7.2)	118.9	4.78 t (7.2)
14	136.0		135.7	
15	18.3	1.58 s	18.0	1.58 s
16	26.0	1.56 s	25.7	1.56 s
17	38.8	2.67 dd (14.3, 7.4)	38.5	2.65 dd (14.2, 7.3)
18	119.2	4.78 t (7.2)	118.9	4.77 t (7.2)
19	136.0		135.7	
20	18.3	1,58 s	18.0	1.58 s
21	26.0	1.56 s	25.7	1.56 s

Tested in methanol-d_4.

Comparison of ^{1}H NMR spectrum of natural **2** and synthetic **2**

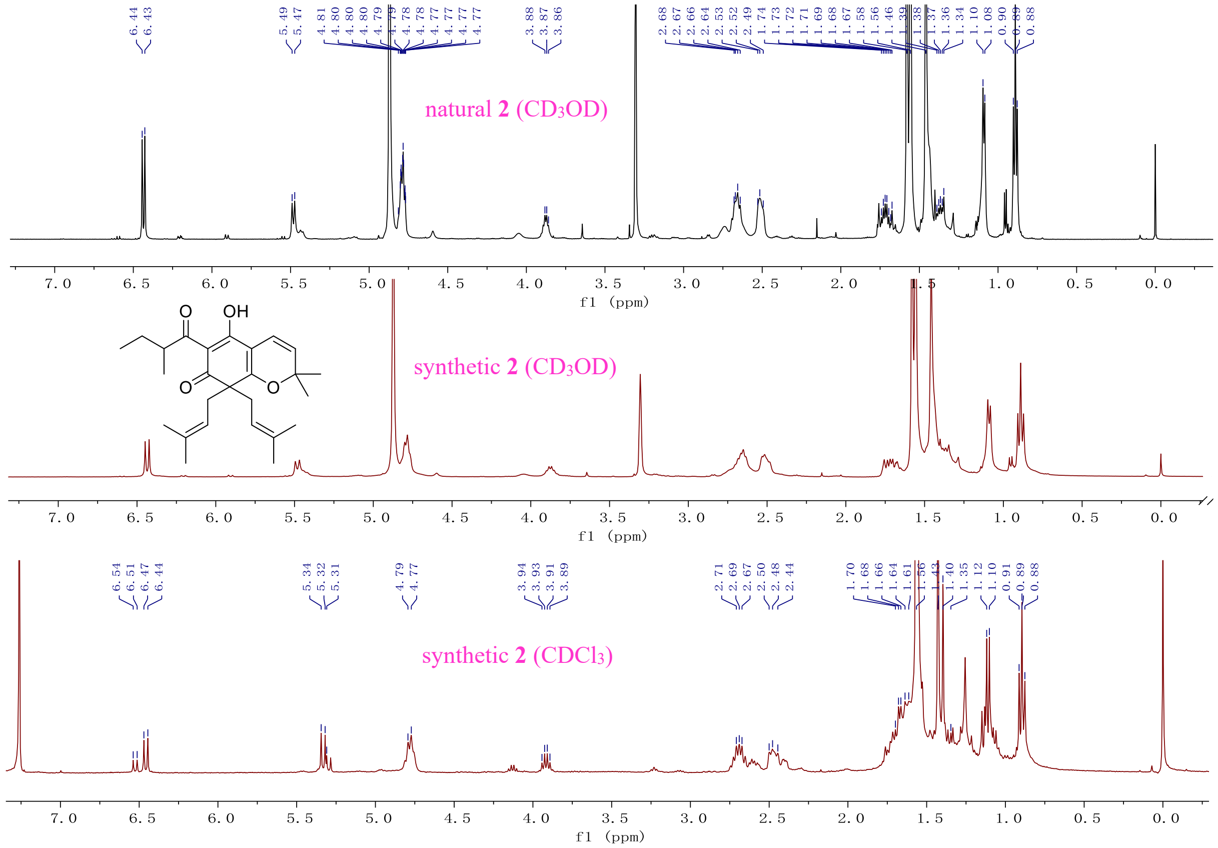

Comparison of ^{13}C NMR spectrum of natural **2** and synthetic **2**

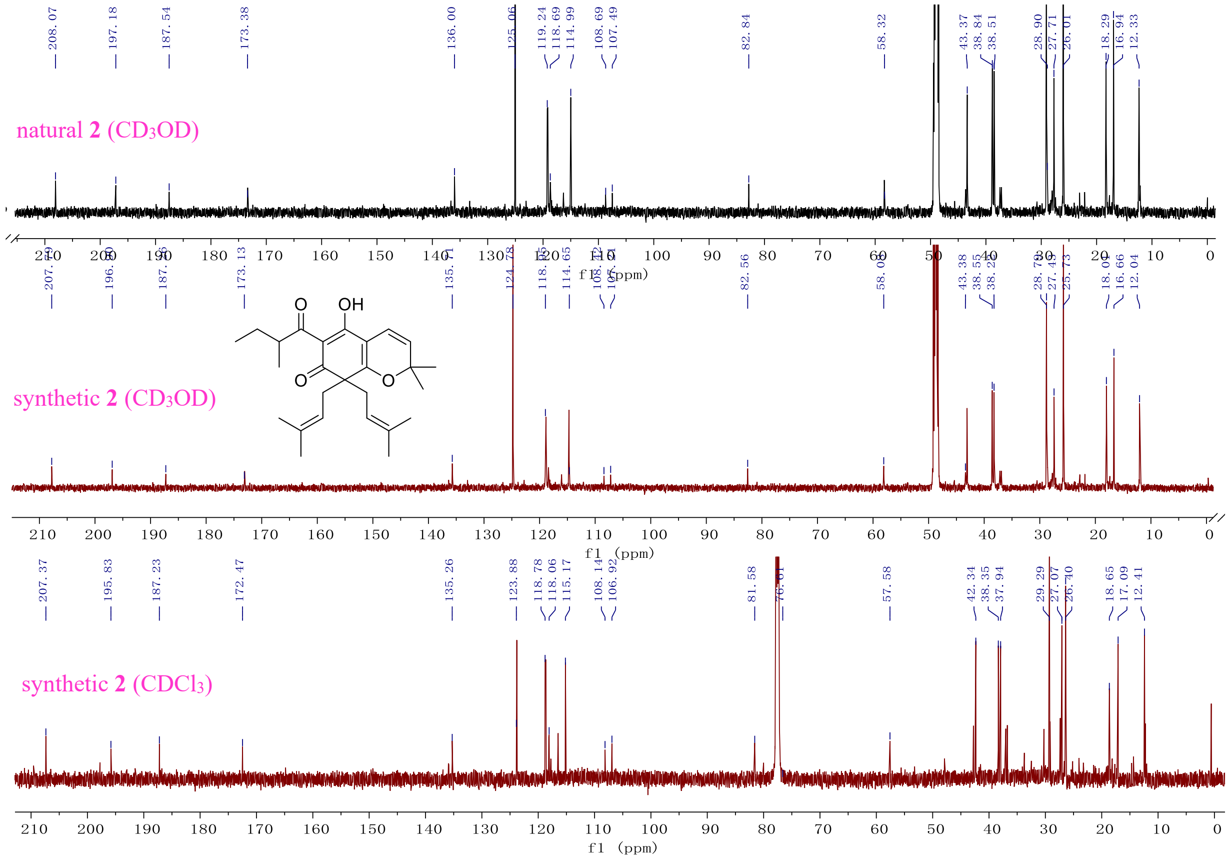

6. HRESI-MS Spectra of all Compounds

HRESI-MS Spectrum of Compound 6a

User Spectra

Fragmentor Voltage	Collision Energy	Ionization Mode
135	0	ESI

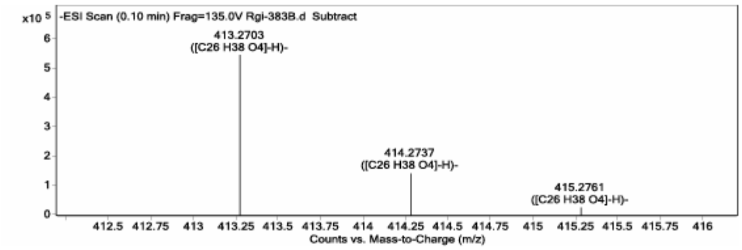

Peak List

m/z	z	Abund	Formula	Ion
195.8103	1	2547.15		
197.807	1	2670.15		
345.2096	1	2705.26		
397.1402	1	5825.69		
413.2703	1	546827.81	C26 H38 O4	(M-H)-
414.2737	1	144996.81	C26 H38 O4	(M-H)-
415.2761	1	26890.71	C26 H38 O4	(M-H)-
416.2806	1	3572.95	C26 H38 O4	(M-H)-
471.2293	1	2505.08		
955.9671	1	4826.44		

Formula Calculator Element Limits

Element	Min	Max
C	3	50
H	0	100
O	0	20

Formula Calculator Results

Formula	CalculatedMass	CalculatedMz	Mz	Diff. (mDa)	Diff. (ppm)	DBE
C26 H38 O4	414.2770	413.2697	413.2703	-0.60	-1.45	8.0000

--- End Of Report ---

HRESI-MS Spectrum of Compound 6b

User Spectra

Fragmentor Voltage	Collision Energy	Ionization Mode
135	0	ESI

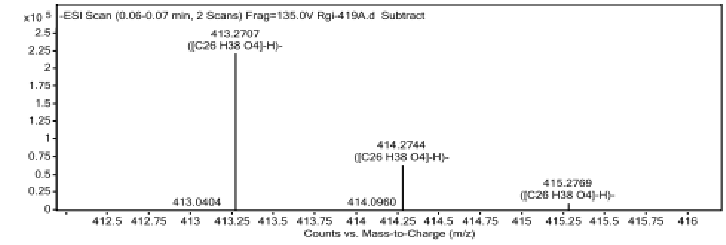

Peak List

m/z	z	Abund	Formula	Ion
112.9854	1	3034.51		
331.1926	1	2028.33		
413.2707	1	221825.45	C26 H38 O4	(M-H)-
414.2744	1	64313	C26 H38 O4	(M-H)-
415.2769	1	10410.66	C26 H38 O4	(M-H)-
416.2792	1	1830.92	C26 H38 O4	(M-H)-
448.3147	1	5192.24		
449.3212	1	10222.59		
450.3256	1	2888.76		
481.3323	1	4445.54		

Formula Calculator Element Limits

Element	Min	Max
C	3	50
H	0	100
O	0	20

Formula Calculator Results

Formula	CalculatedMass	CalculatedMz	Mz	Diff. (mDa)	Diff. (ppm)	DBE
C26 H38 O4	414.2770	413.2697	413.2707	-1.00	-2.42	8.0000

--- End Of Report ---

HRESI-MS Spectrum of Compound **1**

User Spectra

Fragmentor Voltage	Collision Energy	Ionization Mode
135	0	ESI

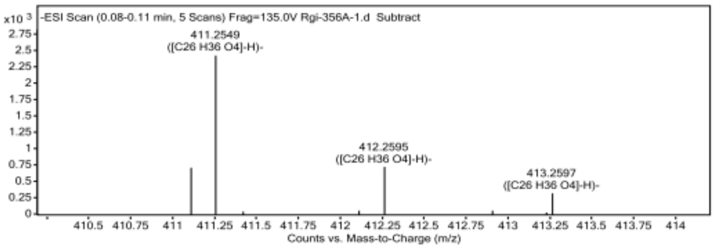

Peak List

m/z	z	Abund	Formula	Ion
68.9954	1	2216.8		
112.9856	1	16101.24		
235.0979	1	7252.67		
297.1145	1	3028.83		
343.1923	1	3307.38		
411.2549	1	2436.44	C26 H36 O4	(M-H)-
496.2334	1	3747.9		
845.4968	1	3094.41		
1257.7437	1	2338.01		
1258.7508	1	2406.69		

Formula Calculator Element Limits

Element	Min	Max
C	3	50
H	0	100
O	0	20

Formula Calculator Results

Formula	CalculatedMass	CalculatedMz	Mz	Diff. (mDa)	Diff. (ppm)	DBE
C26 H36 O4	412.2614	411.2541	411.2549	-0.80	-1.95	9.0000

--- End Of Report ---

ESI-MS Spectrum of Compound **1**

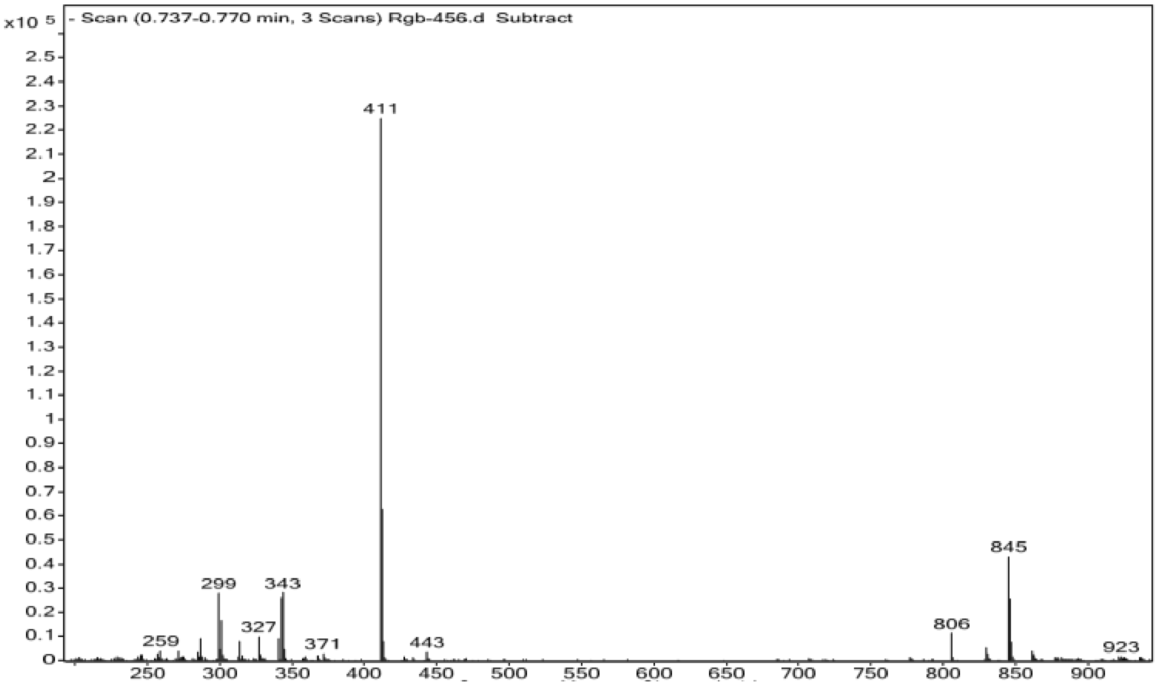

HRESI-MS Spectrum of Compound **2**

User Spectra

Fragmentor Voltage	Collision Energy	Ionization Mode
135	0	ESI

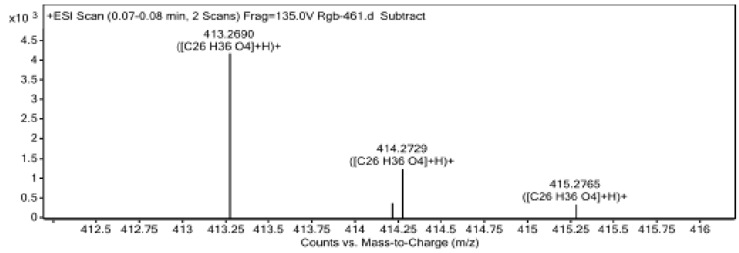

Peak List

m/z	z	Abund	Formula	Ion
274.2749	1	1442.77		
376.2618	1	923.46		
393.286	1	3322.41		
394.2905	1	974.17		
398.2448	1	1038.74		
413.269	1	4183.95	C26 H36 O4	(M+H)+
414.2729	1	1248.29	C26 H36 O4	(M+H)+
421.3191	1	1040.54		
725.5485	1	3186.82		
726.5518	1	1871.67		

Formula Calculator Element Limits

Element	Min	Max
C	3	50
H	0	100
O	0	20

Formula Calculator Results

Formula	CalculatedMass	CalculatedMz	Mz	Diff. (mDa)	Diff. (ppm)	DBE
C26 H36 O4	412.2614	413.2686	413.2690	-0.40	-0.97	9.0000

--- End Of Report ---

ESI-MS Spectrum of Compound **2**

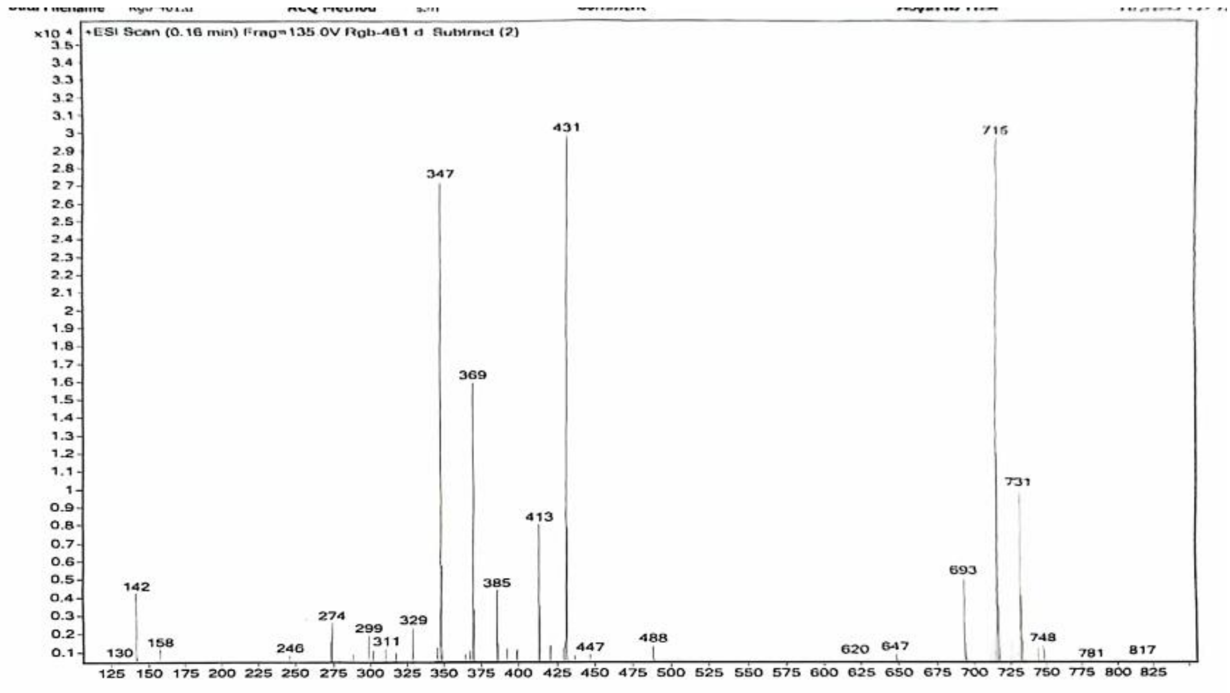

HRESI-MS Spectrum of Compound **7b**

User Spectra

Fragmentor Voltage	Collision Energy	Ionization Mode
135	0	ESI

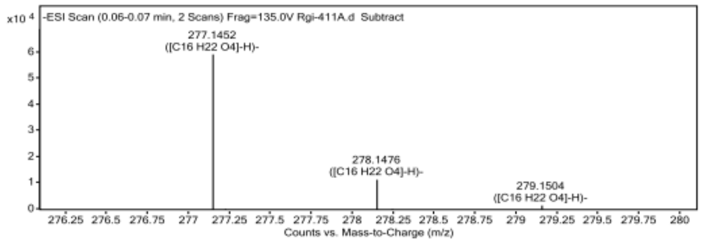

Peak List

m/z	z	Abund	Formula	Ion
61.9883	1	1618.8		
112.9858	1	19485.61		
277.1452	1	59207.21	C16 H22 O4	(M-H)-
278.1476	1	11548.57	C16 H22 O4	(M-H)-
279.1504	1	1570.25	C16 H22 O4	(M-H)-
391.1386	1	5047.15		
555.2958	1	2971.26		
556.2999	1	1330.09		
569.2774	1	2562.2		
1033.9877	1	3301.65		

Formula Calculator Element Limits

Element	Min	Max
C	3	50
H	0	100
O	0	20

Formula Calculator Results

Formula	CalculatedMass	CalculatedMz	Mz	Diff. (mDa)	Diff. (ppm)	DBE
C16 H22 O4	278.1518	277.1445	277.1452	-0.70	-2.53	6.0000

--- End Of Report ---

HRESI-MS Spectrum of Compound **7a**

User Spectra

Fragmentor Voltage	Collision Energy	Ionization Mode
135	0	ESI

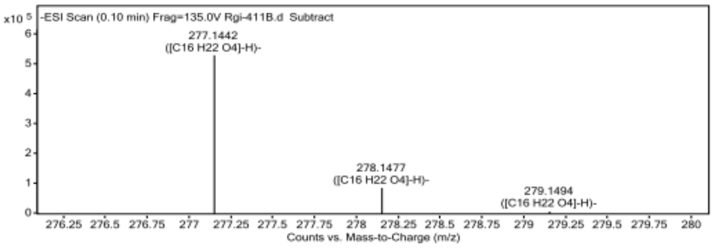

Peak List

m/z	z	Abund	Formula	Ion
112.9855	1	19598.25		
148.9521	1	6428.86		
188.032	1	6191.84		
277.1442	1	529945.38	C16 H22 O4	(M-H)-
278.1477	1	86973.22	C16 H22 O4	(M-H)-
279.1494	1	9845.21	C16 H22 O4	(M-H)-
333.2068	1	9591.66		
345.1308	1	12102.22		
499.2347	1	7554.72		
501.1542	1	6496.57		

Formula Calculator Element Limits

Element	Min	Max
C	3	50
H	0	100
O	0	20

Formula Calculator Results

Formula	CalculatedMass	CalculatedMz	Mz	Diff. (mDa)	Diff. (ppm)	DBE
C16 H22 O4	278.1518	277.1445	277.1442	0.30	1.08	6.0000

--- End Of Report ---

HRESI-MS Spectrum of Compound **8**

User Spectra

Fragmentor Voltage	Collision Energy	Ionization Mode
135	0	ESI

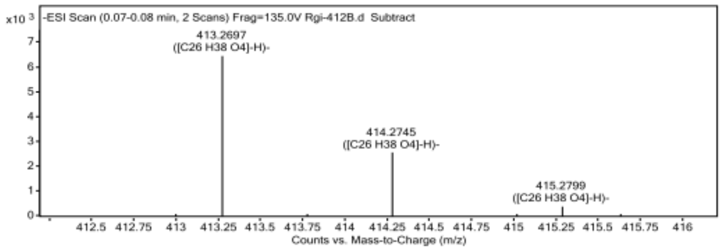

Peak List

m/z	z	Abund	Formula	Ion
112.9857	1	6354.8		
413.2697	1	6477.96	C26 H38 O4	(M-H)-
414.2745	1	2578.76	C26 H38 O4	(M-H)-
481.2556	1	2091.98		
498.2496	1	2649.99		
501.303	1	5278.32		
849.5287	1	1410.83		
912.5106	1	1426.56		
1033.9879	1	3979.42		
1280.7755	1	1496.23		

Formula Calculator Element Limits

Element	Min	Max
C	3	50
H	0	100
O	0	20

Formula Calculator Results

Formula	CalculatedMass	CalculatedMz	Mz	Diff. (mDa)	Diff. (ppm)	DBE
C26 H38 O4	414.2770	413.2697	413.2697	0.00	0.00	8.0000

--- End Of Report ---

HRESI-MS Spectrum of Compound **9**

User Spectra

Fragmentor Voltage	Collision Energy	Ionization Mode
135	0	ESI

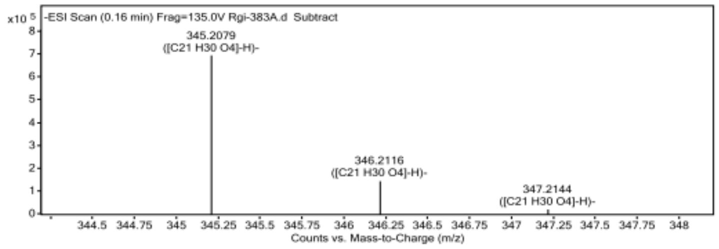

Peak List

m/z	z	Abund	Formula	Ion
112.9858	1	42211.42		
345.2079	1	696295.88	C21 H30 O4	(M-H)-
346.2116	1	149032.83	C21 H30 O4	(M-H)-
347.2144	1	22935.55	C21 H30 O4	(M-H)-
361.2029	1	106853.18		
362.2066	1	30465.74		
396.2476	1	30999.77		
413.2713	1	131723.22		
414.2763	1	83919.92		
415.2794	1	25366.8		

Formula Calculator Element Limits

Element	Min	Max
C	3	50
H	0	100
O	0	20

Formula Calculator Results

Formula	CalculatedMass	CalculatedMz	Mz	Diff. (mDa)	Diff. (ppm)	DBE
C21 H30 O4	346.2144	345.2071	345.2079	-0.80	-2.32	7.0000

--- End Of Report ---

HRESI-MS Spectrum of Compound **10**

User Spectra

Fragmentor Voltage	Collision Energy	Ionization Mode
135	0	ESI

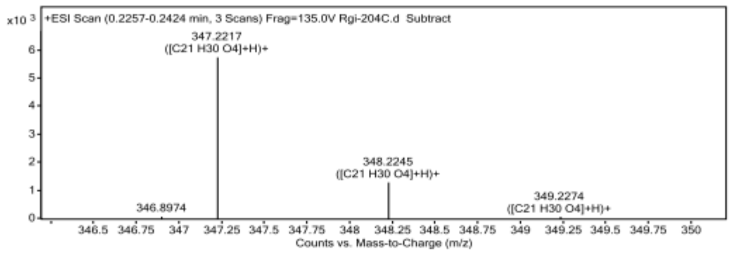

Peak List

m/z	z	Abund	Formula	Ion
130.1223	1	1141.86		
172.0946	1	2757.8		
223.0966	1	1435.07		
266.1291	1	13564.79		
267.1329	1	2461.11		
347.2217	1	5763.36	C21 H30 O4	(M+H)+
348.2245	1	1321.65	C21 H30 O4	(M+H)+
369.203	1	2033.48		
370.2088	1	674.16		
385.1807	1	681.05		

Formula Calculator Element Limits

Element	Min	Max
C	3	60
H	0	100
O	0	20

Formula Calculator Results

Formula	CalculatedMass	CalculatedMz	Mz	Diff. (mDa)	Diff. (ppm)	DBE
C21 H30 O4	346.2144	347.2217	347.2217	0.00	0.00	7.0000

--- End Of Report ---

HRESI-MS Spectrum of Compound **11**

User Spectra

Fragmentor Voltage	Collision Energy	Ionization Mode
200	0	ESI

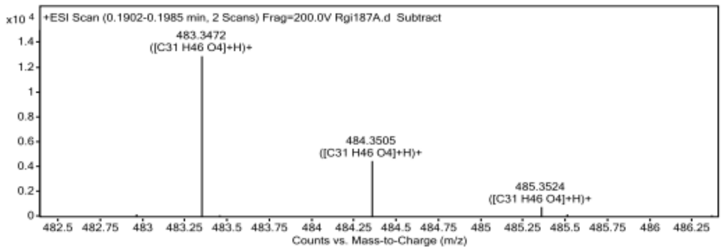

Peak List

m/z	z	Abund	Formula	Ion
274.2742	1	10466.03		
275.1644	1	4642.86		
291.159	1	10964.81		
338.3422	1	6666.12		
347.2216	1	7148.18		
415.2842	1	8570.22		
483.3472	1	12937.95	C31 H46 O4	(M+H)+
505.3287	1	5334.01		
719.4515	1	11247.83		
720.4546	1	4900.15		

Formula Calculator Element Limits

Element	Min	Max
C	3	60
H	0	100
O	0	20

Formula Calculator Results

Formula	CalculatedMass	CalculatedMz	Mz	Diff. (mDa)	Diff. (ppm)	DBE
C31 H46 O4	482.3396	483.3469	483.3472	-0.30	-0.62	9.0000

--- End Of Report ---

HRESI-MS Spectrum of Compound **13**

User Spectra

Fragmentor Voltage	Collision Energy	Ionization Mode
135	0	ESI

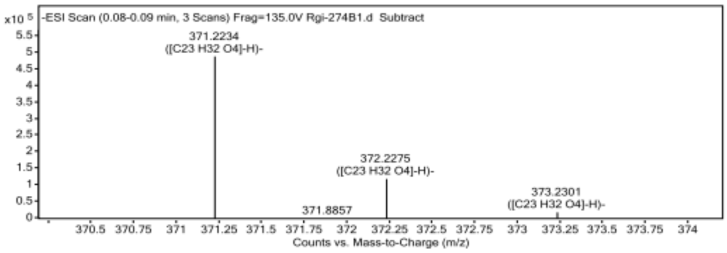

Peak List

m/z	z	Abund	Formula	Ion
289.1448	1	22346.91		
290.1492	1	5047.02		
371.2234	1	488432.34	C23 H32 O4	(M-H)-
372.2275	1	119680.37	C23 H32 O4	(M-H)-
373.2301	1	18643.07	C23 H32 O4	(M-H)-
374.2324	1	2681.46	C23 H32 O4	(M-H)-
387.2176	1	2667.04		
388.223	1	3126.08		
439.2862	1	5513.19		
765.4341	1	2255.9		

Formula Calculator Element Limits

Element	Min	Max
C	3	50
H	0	100
O	0	20

Formula Calculator Results

Formula	CalculatedMass	CalculatedMz	Mz	Diff. (mDa)	Diff. (ppm)	DBE
C23 H32 O4	372.2301	371.2228	371.2234	-0.60	-1.62	8.0000

--- End Of Report ---

HRESI-MS Spectrum of Compound **14**

User Spectra

Fragmentor Voltage	Collision Energy	Ionization Mode
135	0	ESI

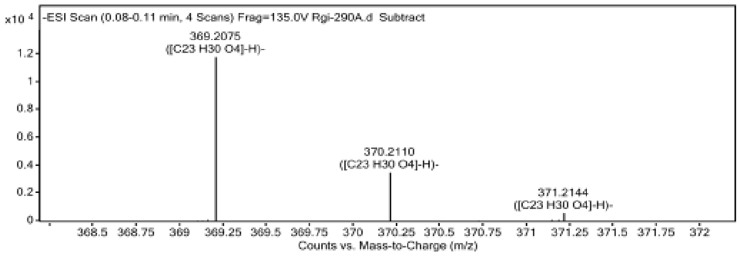

Peak List

m/z	z	Abund	Formula	Ion
61.9884	1	4126.02		
112.9855	1	8366.27		
301.1451	1	10139.2		
369.2075	1	11796.43	C23 H30 O4	(M-H)-
401.1989	1	4646.45		
761.4048	1	5524.3		
966.0017	1	4425.58		
1033.9884	1	12375.27		
1131.6029	1	4105.11		
1163.5583	1	4615.86		

Formula Calculator Element Limits

Element	Min	Max
C	3	50
H	0	100
O	0	20

Formula Calculator Results

Formula	CalculatedMass	CalculatedMz	Mz	Diff. (mDa)	Diff. (ppm)	DBE
C23 H30 O4	370.2144	369.2071	369.2075	-0.40	-1.08	9.0000

--- End Of Report ---

7. UV and IR Spectra of Compounds 1 and 2

UV Spectrum of Compound 1

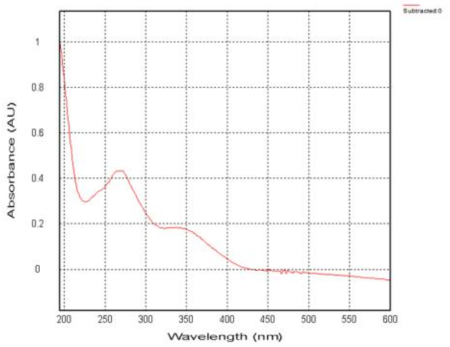

UV Spectrum of Compound 2

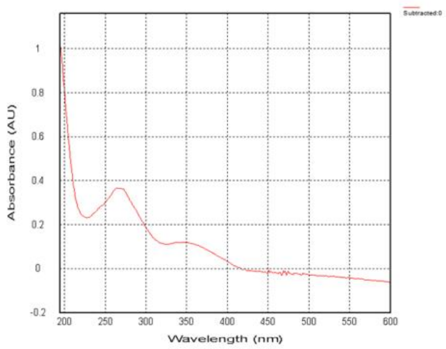

IR Spectrum of Compound **1**

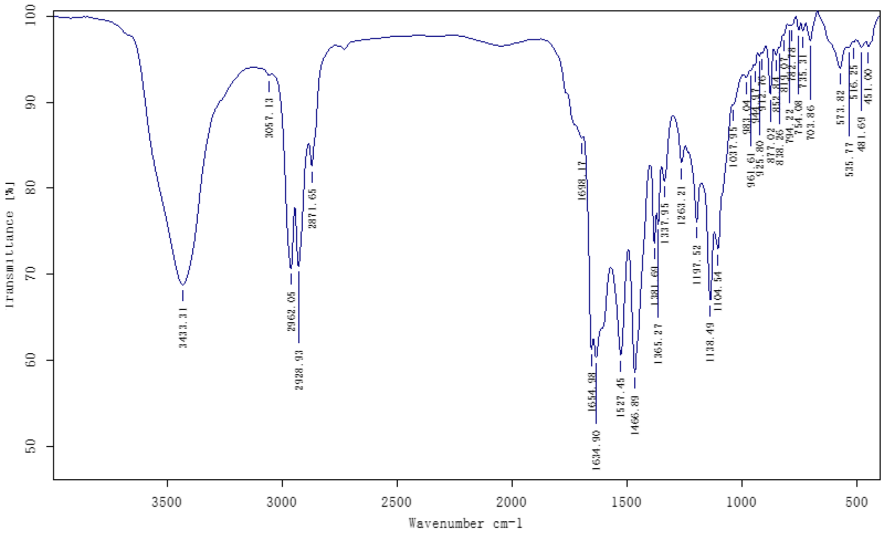

IR Spectrum of Compound **2**

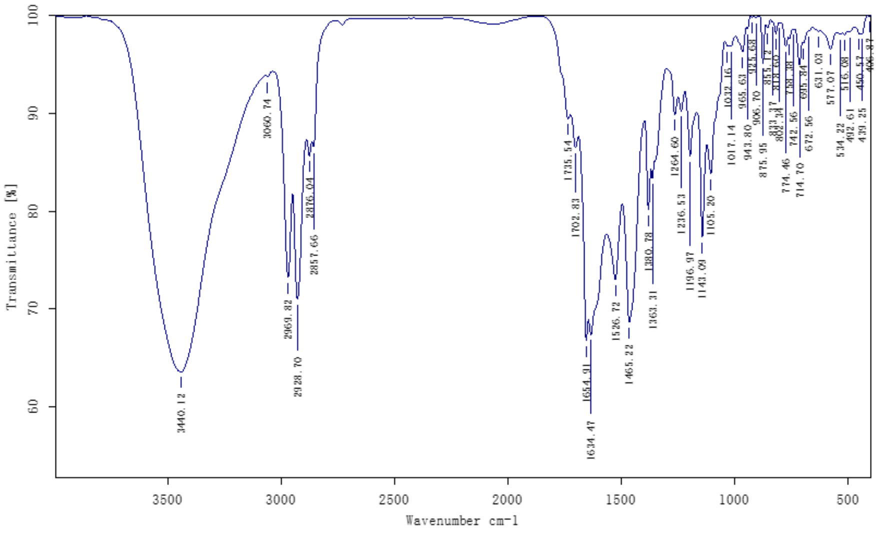

8. Primary Activity Screening

compound **1**

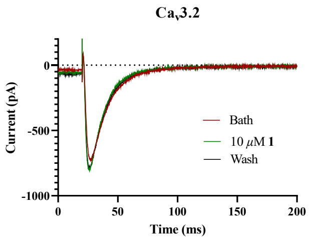

compound **2**

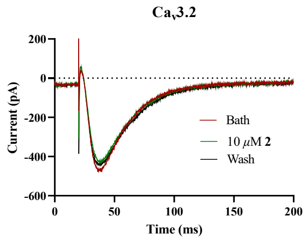

compound **8**

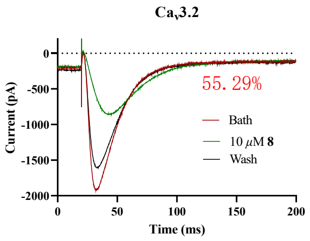

compound **9**

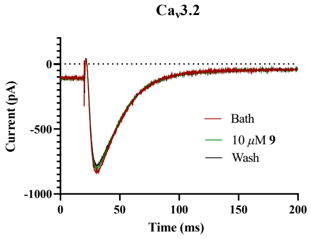

compound **10**

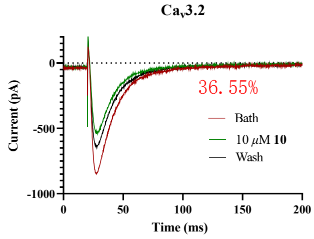

compound **11**

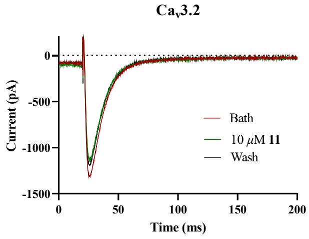

compound **14**

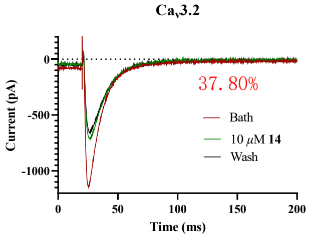

compound **6a**

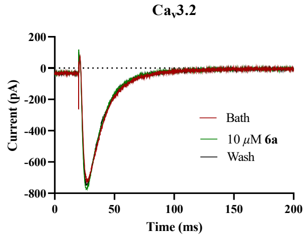

Supplement: Supplementary file 1 — Additional file1 (PDF 6092 KB) [file 13659_2026_626_MOESM1_ESM.pdf]
